# Synthesis of chiral cyclohexane-linked bisimidazolines

**DOI:** 10.3762/bjoc.21.140

**Published:** 2025-09-04

**Authors:** Changmeng Xi, Qingshan Sun, Jiaxi Xu

**Affiliations:** 1 State Key Laboratory of Chemical Resource Engineering, Department of Organic Chemistry, College of Chemistry, Beijing University of Chemical Technology, Beijing 100029, People’s Republic of China

**Keywords:** bisimidazoline, cyclohexane, cyclohexane-1,2-dicarboxylic acid, 1,2-diphenylethane-1,2-diamine

## Abstract

Both chiral bisoxazolines and bisimidazolines are efficient chiral ligands in metal-catalyzed asymmetric organic transformations. Chiral cyclohexane-linked bisimidazolines were prepared from optically active cyclohexane-1,2-dicarboxylic acid and 1,2-diphenylethane-1,2-diamines via the monosulfonylation of 1,2-diphenylethane-1,2-diamines, condensation of *N*-sulfonylated 1,2-diphenylethane-1,2-diamines and cyclohexane-1,2-dicarboxylic acid, and the final cyclization with the in situ generated Hendrickson reagent.

## Introduction

**Figure 1:**
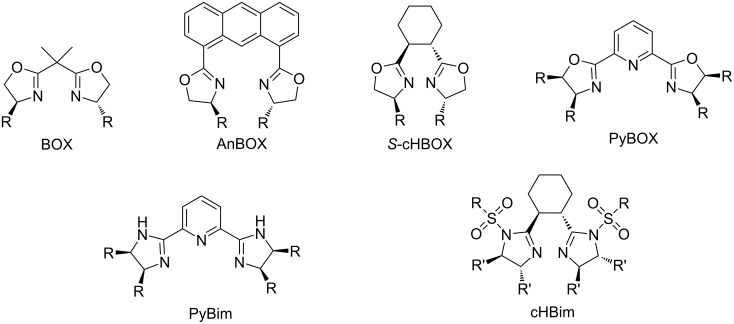
Bisoxazoline and bisimidazoline ligands.

Chiral bisoxazolines [1–9] and bisimidazolines [10–15] are efficient chiral ligands and have been widely applied in various metal-catalyzed asymmetric organic transformations. Various chiral bisoxazoline ligands have been prepared from diacids and enantiopure vicinal amino alcohols and utilized in different metal-catalyzed asymmetric organic reactions [1–9]. In comparison with bisoxazoline ligands, relatively less attention has been paid to bisimidazoline ligands [10–15]. Some well investigated bisimidazoline ligands are pyridine-linked bisimidazoline (PyBim) ligands derived from pyridine-2,6-dicarbonitrile or pyridine-2,6-dicarboxylic acid and vicinal diamines, as analogues of pyridine-linked bisoxazoline (PyBOX) ligands [16–17]. They exhibited excellent performance in metal-catalyzed asymmetric organic reactions. Chiral rigid backbone-linked bisoxazoline ligands, such as anthracene-1,8-linked bisoxazolines (AnBOX) [18–20] showed excellent enantioselectivities for certain substrates due to their ability to fix transition states in asymmetric reactions, realizing excellent stereoselectivities. However, they also presented some limitations to the substrate scope due to their complete rigidity. Cyclohexane-1,2-linked bisoxazolines (cHBOX) are a class of bisoxazoline ligands with the more flexible cyclohexane as linker [21–22]. Chiral cyclohexane-1,2-linked bisoxazolines fix transition states in catalytic asymmetric reactions, in whch the transition states can be regulated depending upon the structures of substrates, achieving excellent stereoselectivities among various substrates in the catalytic asymmetric aziridination of α,β-unsaturated ketones [21]. The steric effect of cHBOX ligands can be changed easily by the use of different chiral vicinal amino alcohols as starting materials in their synthesis. However, it is difficult to tune the electronic effects of cHBOX ligands. Chiral cyclohexane-1,2-linked bisimidazolines possess similar structural features as cHBOX ligands and their electronic effect can be tuned by the introduction of different substituents on the NH moiety of the imidazoline ring. Herein, we designed and synthesized several sulfonylated cyclohexane-1,2-linked bisimidazoline (cHBim) ligands (Figure 1).

## Results and Discussion

**Scheme 1:**
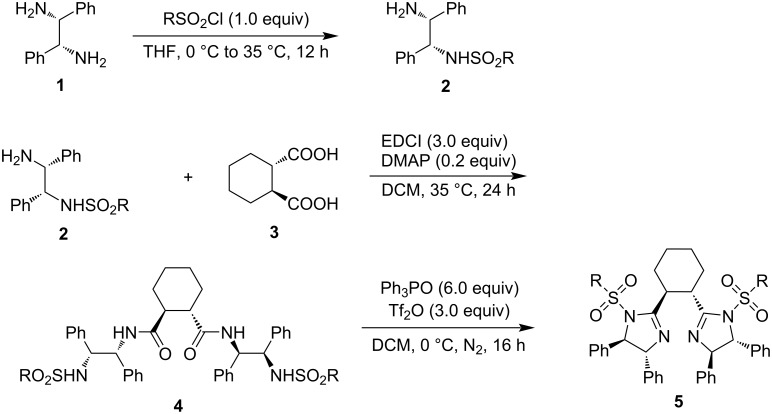
Synthesis of chiral cyclohexane-linked bisimidazoline ligands.

The synthesis of the chiral cyclohexane-linked bisimidazoline ligands started from enantiopure (1*S*,2*S*)-cyclohexane-1,2-dicarboxylic acid (**3**) and (1*R*,2*R*)-1,2-diphenylethane-1,2-diamine (**1**), hoping that the phenyl groups in the designed bisimidazoline ligands stemming from the vicinal diamine play an important role in the stereocontrol of catalytic asymmetric organic reactions [23–26] (Scheme 1). The *C*_2_-symmetric (1*R*,2*R*)-1,2-diphenylethane-1,2-diamine (**1**) was selected as a suitable chiral vicinal diamine to prevent the generation of different isomeric sulfonamides in the reaction with sulfonyl chlorides. Diamine **1** was first reacted with different sulfonyl chlorides to prepare *N*-[(1*R*,2*R*)-2-amino-1,2-diphenylethyl]sulfonamides **2** in 61–74% yields. The sulfonamides **2** were then further reacted with (1*S*,2*S*)-cyclohexane-1,2-dicarboxylic acid (**3**) in the presence of EDCI (3-ethyl-1-(3-dimethylaminopropyl)carbodiimide hydrochloride) as a coupling reagent under the catalysis of DMAP (4-dimethylaminopyridine), affording (1*S*,2*S*)-*N*^1^,*N*^2^-bis((1*R*,2*R*)-2-(sulfonamido)-1,2-diphenylethyl)cyclohexane-1,2-dicarboxamides **4** in 32–87% yields. Electron-deficient sulfonamides **2a**, **2e** and **2f** with both electron-donating methyl and strong electron-withdrawing trifluoromethyl and 4-nitrophenyl groups showed low reactivity in the formation of cyclohexane-1,2-dicarboxamides **4** due to their poor nucleophilicity. Finally, (1*S*,2*S*)-*N*^1^,*N*^2^-bis((1*R*,2*R*)-2-(sulfonamido)-1,2-diphenylethyl)cyclohexane-1,2-dicarboxamides **4** were cyclized with the Hendrickson reagent (triphenylphosphonium anhydride triflate in situ generated from triphenylphosphine and triflic anhydride) as an activating reagent [27], giving rise to (1*S*,2*S*)-1,2-bis((4*R*,5*R*)-1-(sulfonyl)-4,5-diphenyl-4,5-dihydro-1*H*-imidazol-2-yl)cyclohexanes **5** in 30–70% yields. 1,2-Cyclohexane-1,2-dicarboxamide **4e** with two strong electron-poor 4-nitrophenyl groups generated the corresponding cyclized product **5e** in a low yield of 30% due to its poor nucleophilicity. However, 1,2-cyclohexane-1,2-dicarboxamide **4f** with two very strong electron-poor trifluoromethyl groups did not undergo cyclization due to its very poor nucleophilicity. The results are collected in Table 1.

**Table 1:** Synthesis of chiral cyclohexane-linked bisimidazoline ligands.

Entry	Compd	R	Configuration of diamine **1**	Yield of isolated product/%

1	**2a**	Me	*R,R*	67
2	**2b**	Ph	*R,R*	74
3	**2c**	4-MeC_6_H_4_	*R,R*	61
4	**2d**	4-MeOC_6_H_4_	*R,R*	64
5	**2e**	4-O_2_NC_6_H_4_	*R,R*	72
6	**2f**	F_3_C	*R,R*	61
7	**2g**	4-MeC_6_H_4_	*S,S*	61
8	**4a**	Me	*R,R*	38
9	**4b**	Ph	*R,R*	71
10	**4c**	4-MeC_6_H_4_	*R,R*	73
11	**4d**	4-MeOC_6_H_4_	*R,R*	87
12	**4e**	4-O_2_NC_6_H_4_	*R,R*	52
13	**4f**	F_3_C	*R,R*	32
14	**4g**	4-MeC_6_H_4_	*S,S*	70
15	**5a**	Me	*R,R*	51
16	**5b**	Ph	*R,R*	57
17	**5c**	4-MeC_6_H_4_	*R,R*	36
18	**5d**	4-MeOC_6_H_4_	*R,R*	70
19	**5e**	4-O_2_NC_6_H_4_	*R,R*	30
20	**5g**	4-MeC_6_H_4_	*S,S*	36

Following the similar strategy, (1*S*,2*S*)-1,2-bis((4*S*,5*S*)-1-(4-methylphenylsulfonyl)-4,5-diphenyl-4,5-dihydro-1*H*-imidazol-2-yl)cyclohexane (**5g**) was also prepared from enantiopure (1*S*,2*S*)-cyclohexane-1,2-dicarboxylic acid (**3**) and (1*S*,2*S*)-1,2-diphenylethane-1,2-diamine ((*S*,*S*)-**1**) to study the influence of different configurations of bisimidazoline ligands **5** on the stereocontrol in catalytic asymmetric reactions (Table 1).

**Scheme 2:**
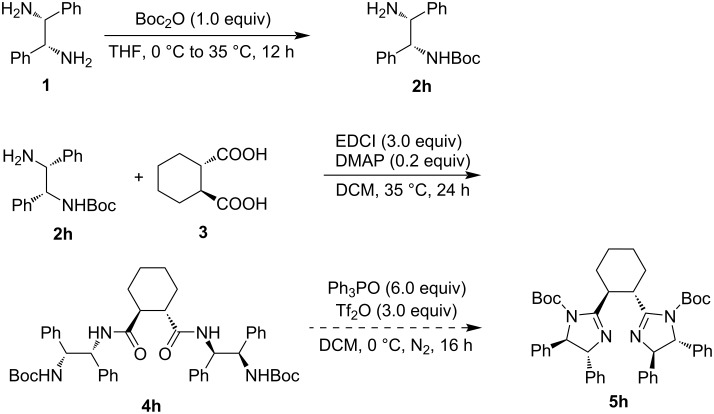
Attempted synthesis of chiral cyclohexane-linked bisimidazoline **5h**.

To improve the synthetic efficiency, a different strategy for the synthesis of a nonsulfonylated cyclohexane-linked bisimidazoline with subsequent sulfonylation with different sulfonyl chlorides was also considered and attempted (Scheme 2). For this purpose, *tert*-butyl *N*-[(1*R*,2*R*)-2-amino-1,2-diphenylethyl]carbamate (**2h**) with an acid-sensitive Boc (*tert*-butoxycarbonyl) protecting group was prepared in 70% yield from *C*_2_-symmetric (1*R*,2*R*)-1,2-diphenylethane-1,2-diamine (**1**) and Boc_2_O. Compound **2h** was then converted to (1*S*,2*S*)-*N*^1^,*N*^2^-bis((1*R*,2*R*)-2-(*tert*-butoxycarbonyl)-1,2-diphenylethyl)cyclohexane-1,2-dicarboxamide (**4h**) in 65% yield through the reaction with (1*S*,2*S*)-cyclohexane-1,2-dicarboxylic acid (**3**). However, compound **4h** did not undergo cyclization possibly due to weak nucleophilicity and steric hindrance of the Boc-protected amino group.

**Scheme 3:**
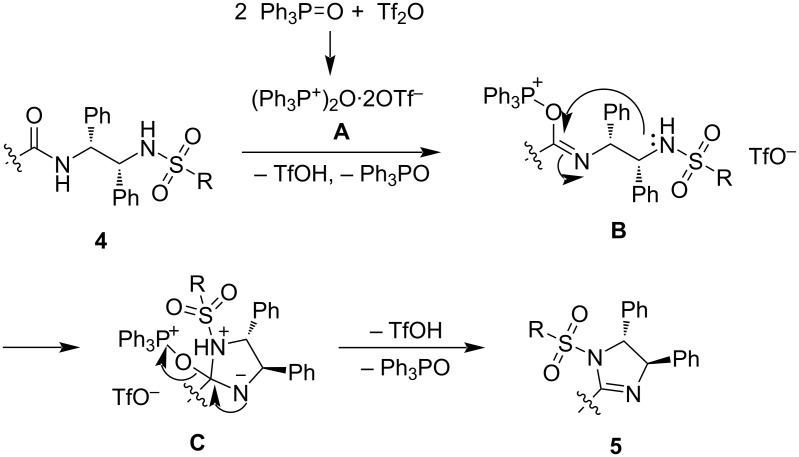
Proposed reaction mechanism.

On the basis of the previous report [28], a possible reaction mechanism is presented in Scheme 3. The reaction of triphenylphosphine oxide and triflic anhydride first generates an activating agent, the Hendrickson reagent (**A**). The amide in cyclohexane-1,2-dicarboxamides **4** nucleophilically attacks the phosphonium in **A** to generate intermediate **B** by loss of triphenylphosphine oxide and triflic acid. The nucleophilic sulfonamide in **B** intramolecularily attacks the generated imine moiety in **B** to form intermediate **C**, in which triflic acid may protonate the imine moiety in **B** to assist the nucleophilic attack. Intermediate **C** further transforms to imidazoline product **5** by loss of triphenylphosphine oxide and triflic acid.

## Conclusion

Both chiral bisoxazolines and bisimidazolines are efficient and widely applied chiral ligands in metal-catalyzed asymmetric organic reactions. Several chiral cyclohexane-linked bisimidazolines were prepared from enantiopure (1*S*,2*S*)-cyclohexane-1,2-dicarboxylic acid and (1*R*,2*R*)- and (1*S*,2*S*)-1,2-diphenylethane-1,2-diamines via the monosulfonylation of 1,2-diphenylethane-1,2-diamines, condensation of *N*-sulfonylated 1,2-diphenylethane-1,2-diamines with (1*S*,2*S*)-cyclohexane-1,2-dicarboxylic acid followed by the Hendrickson reagent-mediated final cyclization.

## Supporting Information

File 1 Analytical data and copies of ^1^H and ^13^C NMR spectra of compounds **2** and **4**, copies of HRMS spectra of unknown compounds **4** and **5**.

## Data Availability

All data that supports the findings of this study is available in the published article and the supporting information of this article.
