# Supplementary material for: Synthesis of chiral cyclohexane-linked bisimidazolines
Source: Beilstein J Org Chem. 2025 Sep 4;21:1786–90. doi: 10.3762/bjoc.21.140 (PMC12415896; doi:10.3762/bjoc.21.140)

BEILSTEIN JOURNAL OF ORGANIC CHEMISTRY

# Supporting Information

for

# Synthesis of chiral cyclohexane-linked bisimidazolines

Changmeng Xi, Qingshan Sun and Jiaxi Xu

*Beilstein J. Org. Chem.* **2025,** *21,* 1786–1790. doi:10.3762/bjoc.21.140

## Analytical data and copies of $^{1}H$ and $^{13}C$ NMR spectra of compounds 2 and 4, copies of HRMS spectra of unknown compounds 4 and 5

License and Terms: This is a supporting information file under the terms of the Creative Commons Attribution License (https://creativecommons.org/licenses/by/4.0). Please note that the reuse, redistribution and reproduction in particular requires that the author(s) and source are credited and that individual graphics may be subject to special legal provisions.
The license is subject to the *Beilstein Journal of Organic Chemistry* terms and conditions: (https://www.beilstein-journals.org/bjoc/terms)

## Table of contents

1. General information ........................................................................................ S2

2. General procedure for the synthesis of *N*-[(1*R*,2*R*)-2-amino-1,2-diphenylethyl]-sulfonamides **2**................................................................................................ S2

3 General procedure for the synthesis of (1*S*,2*S*)-*N*[1],*N*[2]-bis((1*R*,2*R*)-2-(sulfon amido)-1,2-diphenylethyl)cyclohexane-1,2-dicarboxamides **4**........................................ S4

4. General procedure for the synthesis of (1*S*,2*S*)-1,2-bis((4*R*,5*R*)-1-(sulfonyl)-4,5-diphenyl-4,5-dihydro-1*H*-imidazol-2-yl)cyclohexanes **5**.................................................. S7

5. References ........................................................................................................ S10

6. Copies of $^1$H and $^{13}$C NMR spectra of compounds **2** and **4** and HRMS spectra of products **4** and **5** ........................................................................................ S11

# 1. General information

Unless otherwise noted, all materials were purchased from commercial suppliers. THF was refluxed over sodium with benzophenone as an indicator and freshly distilled prior to use. DCM (dichloromethane) was refluxed over $CaH_2$ and freshly distilled prior to use. Column chromatography was performed on silica gel (normal phase, 200–300 mesh) from Anhui Liangchen Silicon Material Co., Ltd. Petroleum ether (PE, 60–90 °C fraction) and ethyl acetate (EA) were used as eluent. Reactions were monitored by thin-layer chromatography (TLC) on GF254 silica gel plates (0.2 mm) from Anhui Liangchen Silicon Material Co., Ltd. The plates were visualized by UV light. $^1$H NMR (400 MHz) and $^{13}$C NMR (101 MHz) spectra were recorded on a Bruker 400 NMR spectrometer (Billerica, MA, USA), usually with TMS as an internal standard for $^1$H NMR and the middle peak of $CDCl_3$ as an internal standard (77.16) for $^{13}$C NMR in $CDCl_3$ solution or with DMSO as an internal standard (2.50) for $^1$H NMR and the middle peak of DMSO-$d_6$ as an internal standard (39.5) for $^{13}$C NMR in DMSO-$d_6$ solution. The chemical shifts (δ) were reported in parts per million (ppm) relative to tetramethylsilane (TMS). Melting points were obtained on a melting point apparatus. HRMS measurements were carried out on an LC/MSD TOF mass spectrometer. Specific rotations were measured on an Anton Paar MCP500 polarimeter (Singapore) and reported as follows: $[\alpha]_\lambda^T$ (*c*: g/100 mL, in solvent). The enantiomeric excesses were determined using chiral HPLC analysis using an Agilent 1260 LC instrument (Santa Clara, CA, USA) with a Daicel Chiralcel AD-H column (Hyderabad, India) with a mixture of isopropyl alcohol and hexane as eluents.

# 2. General procedure for the synthesis of *N*-[(1*R*,2*R*)-2-amino-1,2-diphenylethyl]sulfonamides 2

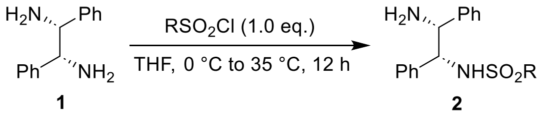


(1*R*,2*R*)-1,2-Diphenylethane-1,2-diamine (**1**, 1.06 g, 5 mmol) and $Et_3N$ (1.4 mL, 10 mmol) were dissolved in anhydrous THF (20 mL) at 0 °C. After 10 min, a solution of sulfonyl chloride (5 mmol) in anhydrous THF (10 mL) was added dropwise under stirring. The reaction temperature was allowed to warm to 35 °C. The reaction mixture was stirred for 12 h. After concentration under reduced pressure, the residue was subjected to silica gel column chromatography with DCM/MeOH 20:1(*v*/*v*) as eluent to afford colorless crystals **2**.

### *N*-[(1*R*,2*R*)-2-Amino-1,2-diphenylethyl]methanesulfonamide (2a)[1]

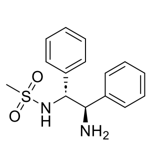


Colorless crystals, 0.88 g, yield: 67%, M.p. 109.7–110.6 °C; $R_f$ = 0.28 (DCM/MeOH 40:1, *v*/*v*), $[\alpha]_D^{25}$ = +8.50 (*c* = 2.0, $CHCl_3$). **$^1$H NMR** (400 MHz, $CDCl_3$) *δ*: 7.37–7.29 (m, 8H, ArH), 7.29–7.22 (m, 2H, ArH), 4.56 (d, *J* = 5.1 Hz, 1H, CH), 4.28 (d, *J* = 5.1 Hz, 1H, CH), 2.26 (s, 3H, $CH_3$). **$^{13}$C NMR** (101 MHz, $CDCl_3$) *δ*: 142.0, 139.8, 128.80, 128.75, 127.98, 127.94, 127.0, 126.8, 63.6, 60.3, 40.8.

**$N$-[(1$R$,2$R$)-2-Amino-1,2-diphenylethyl]benzenesulfonamide (2b)[2]**

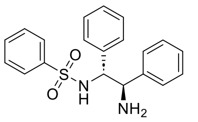

Colorless crystals, 1.30 g, yield: 74%, M.p. 139.7–142.0 °C; $R_f$ = 0.30 (DCM/MeOH 40:1, *v*/*v*), $[\alpha]_D^{25}$ = –8.50 ($c$ = 2.0, $CHCl_3$). **$^1$H NMR** (400 MHz, $CDCl_3$) $\delta$: 7.47–7.41 (m, 2H, ArH), 7.34 (t, $J$ = 7.4 Hz, 1H, ArH), 7.22–7.08 (m, 12H, ArH), 4.41 (d, $J$ = 5.3 Hz, 1H, CH), 4.13 (d, $J$ = 5.3 Hz, 1H, CH). **$^{13}$C NMR** (101 MHz, $CDCl_3$) $\delta$: 141.5, 140.3, 139.2, 132.0, 128.63, 128.57, 128.4, 127.7, 127.6, 127.1, 126.9, 126.6, 63.4, 60.6.

**(1$R$,2$R$)-$N$-$p$-Toluenesulfonyl-1,2-diphenylethylenediamine (2c)[1]**

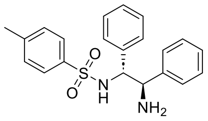

Colorless crystals, 1.12 g, yield: 61%, M.p.122.4–124.1 °C; $R_f$ = 0.33 (DCM/MeOH 40:1, *v*/*v*), $[\alpha]_D^{25}$ = –25.250 ($c$ = 2.0, $CHCl_3$); Lit.[3] $[\alpha]_D^{25}$ = – 17.4 ($c$ 1.3, $CHCl_3$). **$^1$H NMR** (400 MHz, $CDCl_3$) $\delta$: 7.31 (d, $J$ = 8.3 Hz, 2H, ArH), 7.20–7.04 (m, 10H, ArH), 6.96 (d, $J$ = 8.0 Hz, 2H, ArH), 4.39 (d, $J$ = 5.5 Hz, 1H, CH), 4.15 (d, $J$ = 5.5 Hz, 1H, CH), 2.31 (s, 3H, $CH_3$). **$^{13}$C NMR** (101 MHz, $CDCl_3$) $\delta$: 142.6, 141.4, 139.3, 137.3, 129.2, 128.5, 128.3, 127.6, 127.5, 127.2, 127.0, 126.7, 63.4, 60.6, 21.5.

**$N$-[(1$R$,2$R$)-2-Amino-1,2-diphenylethyl]-4-methoxybenzenesulfonamide (2d)[1]**

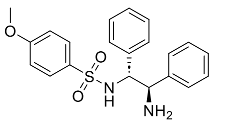

Colorless crystals, 1.20 g, yield: 64%, M.p. 127.5–131.4 °C; $R_f$ = 0.17 (DCM/MeOH 40:1, *v*/*v*), $[\alpha]_D^{25}$ = –22.65 ($c$ = 2.0, $CHCl_3$). **$^1$H NMR** (400 MHz, $CDCl_3$) $\delta$: 7.40–7.31 (m, 2H, ArH), 7.23–7.07 (m, 10H, ArH), 6.68–6.60 (m, 2H, ArH), 4.36 (d, $J$ = 5.4 Hz, 1H, CH), 4.12 (d, $J$ = 5.4 Hz, 1H, CH), 3.79 (s, 3H, $CH_3$). **$^{13}$C NMR** (101 MHz, $CDCl_3$) $\delta$: 162.4, 141.6, 139.4, 132.0, 129.1, 128.6, 128.4, 127.7, 127.5, 127.2, 126.7, 113.8, 63.3, 60.7, 55.6.

**$N$-[(1$R$,2$R$)-2-Amino-1,2-diphenylethyl]-4-nitrobenzenesulfonamide (2e)[4]**

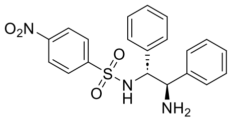

Colorless crystalsc, 1.43 g, yield: 72%, M.p. 196.7–198.1 °C; $R_f$ = 0.30 (DCM/MeOH 40:1, *v*/*v*), $[\alpha]_D^{25}$ = –7.65 ($c$ = 2.0, $CHCl_3$). **$^1$H NMR** (400 MHz, $CDCl_3$) $\delta$: 7.96 (d, $J$ = 8.9 Hz, 2H, ArH), 7.53 (d, $J$ = 8.8 Hz, 2H, ArH), 7.24–7.18 (m, 5H, ArH), 7.18–7.11 (m, 5H, ArH), 4.49 (d, $J$ = 4.5 Hz, 1H, CH), 4.22 (d, $J$ = 4.5 Hz, 1H, CH). **$^{13}$C NMR** (101 MHz, DMSO-$d_6$) $\delta$: 148.7, 146.8, 142.4, 139.5, 127.7, 127.6, 127.5, 127.3, 126.58, 126.55, 123.8, 65.0, 60.4.

**$N$-[(1$R$,2$R$)-2-Amino-1,2-diphenylethyl]-1,1,1-trifluoromethanesulfonamide (2f)[5]**

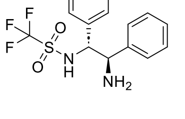

Colorless crystals, 1.15 g, yield: 61%, M.p. 147.5–148.3 °C; $R_f$ = 0.35 (DCM/MeOH 40:1, *v*/*v*), $[\alpha]_D^{25}$ = –3.95 ($c$ = 2.0, $CHCl_3$). **$^1$H NMR** (400 MHz, $CDCl_3$) $\delta$: 7.44–7.30 (m, 10H, ArH), 4.71 (d, $J$ = 3.3 Hz, 1H, CH), 4.40 (d, $J$ = 3.3 Hz, 1H, CH). **$^{13}$C NMR** (101 MHz, $CDCl_3$) $\delta$: 140.4, 139.3, 129.0, 128.9, 128.4, 128.3, 126.5, 126.2, 64.3, 60.3. **$^{19}$F NMR** (376 MHz, $CDCl_3$) $\delta$: -77.87.

**(1*S*,2*S*)-*N*-*p*-Toluenesulfonyl-1,2-diphenylethylenediamine (2g)**[6]

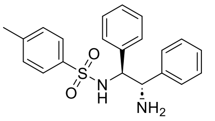

Colorless crystals, 1.12 g, yield: 61%, M.p.122.3–124.2 °C; $R_f$ = 0.33 (DCM/MeOH 40:1, *v*/*v*), $[\alpha]_D^{25}$ = +26.90 (*c* = 2.0, $CHCl_3$); Lit.[7] $[\alpha]_D^{22}$ = +30.0 (c = 1.0, $CHCl_3$). **$^1$H NMR** (400 MHz, $CDCl_3$) *δ*: 7.31 (d, *J* = 8.3 Hz, 2H, ArH), 7.21–7.06 (m, 10H, ArH), 6.97 (d, *J* = 8.0 Hz, 2H, ArH), 4.37 (d, *J* = 5.3 Hz, 1H, CH), 4.12 (d, *J* = 5.3 Hz, 1H, CH), 2.31 (s, 3H, $CH_3$); **$^{13}$C NMR** (101 MHz, $CDCl_3$) *δ*: 142.6, 141.6, 139.4, 137.3, 129.2, 128.5, 128.4, 127.54, 127.47, 127.1, 127.0, 126.7, 63.3, 60.6, 21.5.

**1,1-Dimethylethyl *N*-[(1*R*,2*R*)-2-amino-1,2-diphenylethyl]carbamate (2h)**[8]

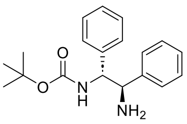

Yellow crystals, 2.17 g, yield: 70%, M.p. 106.4–107.7 °C; $R_f$ = 0.50 (DCM/MeOH 20:1, *v*/*v*), $[\alpha]_D^{25}$ = +13.30 (*c* = 2.0, $CHCl_3$); Lit.[9] $[\alpha]_D^{22}$ = +29.15 (c = 0.5, $CHCl_3$). **$^1$H NMR** (400 MHz, $CDCl_3$) *δ*: 7.43–7.18 (m, 10H, ArH), 5.84 (d, *J* = 8.5 Hz, 1H, NH), 4.89 (s, 1H, CH), 4.37 (d, *J* = 4.1 Hz, 1H, CH), 1.36 (s, 9H, $3CH_3$); **$^{13}$C NMR** (101 MHz, $CDCl_3$) *δ*: 155.8, 142.4, 141.1, 128.6, 128.5, 127.5, 127.3, 126.9, 126.6, 79.4, 60.1, 28.4.

# 3 General procedure for the synthesis of (1*S*,2*S*)-*N*$^1$,*N*$^2$-bis((1*R*,2*R*)-2-(sulfonamido)-1,2-diphenylethyl)cyclohexane-1,2-dicarboxamides 4

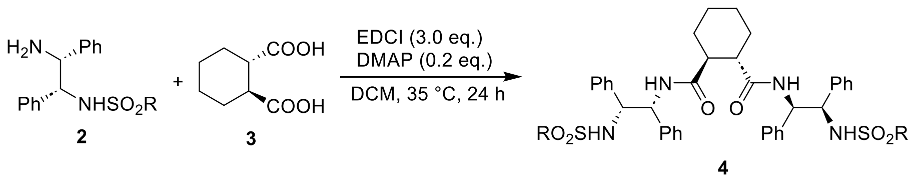


(*S*,*S*)-Cyclohexane-1,2-dicarboxylic acid (**3**, 120 mg, 0.7 mmol), EDCI (403 mg, 2.1 mmol), and DMAP (17 mg, 0.14 mmol) were dissolved in anhydrous DCM (5 mL). The mixture was stirred at 25 °C for 50 min. A solution of product **2** (1.4 mmol) in dry DCM (5 mL) was added dropwise at the same temperature. The reaction temperature was allowed to warm to 35 °C. The reaction mixture was stirred for 24 h. Colorless precipitates appeared. Petroleum ether (10 mL) was added to assist formation of precipitates. After filtration, the precipitates were washed with petroleum ether and recrystallized from methanol to give colorless crystals **4**.

**(1*S*,2*S*)-*N*$^1$,*N*$^2$-Bis((1*R*,2*R*)-2-(methylsulfonamido)-1,2-diphenylethyl)cyclohexane-1,2-dicarboxamide (4a)**

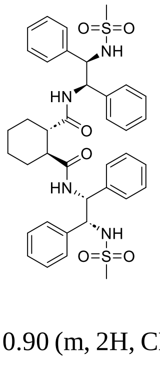

Colorless crystals, 336 mg, yield: 38%, M.p. 283.5–285.2 °C; $R_f$ = 0.25 (DCM/MeOH 20:1, *v*/*v*). $[\alpha]_D^{25}$ = –13.55 (*c* = 2.0, DMSO). **$^1$H NMR** (400 MHz, DMSO-*d*$_6$) *δ*: 7.92 (d, *J* = 9.7 Hz, 2H, 2NH), 7.64 (d, *J* = 9.9 Hz, 2H, 2NH), 7.38 (d, *J* = 7.2 Hz, 4H, ArH), 7.28 (t, *J* = 7.5 Hz, 4H, ArH), 7.24–7.15(m, 6H, ArH), 7.05–6.95 (m, 6H, ArH), 5.20 (dd, *J* = 9.6, 4.7 Hz, 2H, 2CH), 4.78 (dd, *J* = 10.1, 4.8 Hz, 2H, 2CH), 2.37–2.30 (m, 2H, 2CH), 2.07 (s, 6H, $2CH_3$), 1.62 (d, *J* = 8.5 Hz, 2H in $CH_2$), 1.53 (d, *J* = 12.6 Hz, 2H in $CH_2$), 1.20–1.09 (m, 2H, $CH_2$), 1.05–0.90 (m, 2H, $CH_2$). **$^{13}$C NMR** (101 MHz, DMSO-*d*$_6$) *δ*: 173.8, 140.3, 139.8, 127.8, 127.7, 127.03, 126.98, 126.7,

62.0, 56.3, 45.6, 40.8, 29.3, 25.1. **HRMS (ESI)**: *m/z* calcd for $C_{38}H_{45}N_4O_6S_2^+$ $[M+H]^+$: 717.2775, found: 717.2778.

**(1*S*,2*S*)-*N*[1],*N*[2]-Bis((1*R*,2*R*)-2-(phenylsulfonamido)-1,2-diphenylethyl)cyclohexane-1,2-dicarboxamide (4b)**

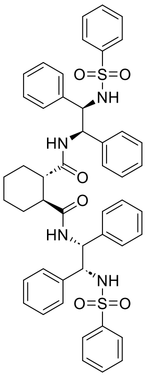


Colorless crystals, 434 mg, yield: 71%, M.p. 256.5–259.3 °C; $R_f$ = 0.25 (DCM/MeOH 20:1, *v*/*v*). $[\alpha]_D^{25}$ = –30.80 (*c* = 2.0, DMSO). **$^{1}$H NMR** (400 MHz, DMSO-$d_6$) $\delta$: 7.51–7.42 (m, 4H, 4NH), 7.34 (d, *J* = 7.5 Hz, 2H, ArH), 7.18 (t, *J* = 7.9 Hz, 4H, ArH), 7.15–7.00 (m, 6H, ArH), 7.02–6.94 (m, 2H, ArH), 6.91 (t, *J* = 7.3 Hz, 4H, ArH), 6.88–6.81 (m, 4H, ArH), 6.77 (d, *J* = 7.8 Hz, 2H, ArH), 6.73–6.66 (m, 4H, ArH), 6.34 (d, *J* = 7.6 Hz, 2H, ArH), 4.95 (dd, *J* = 10.0, 7.7 Hz, 2H, 2CH), 4.59 (dd, *J* = 10.0, 7.7 Hz, 2H, 2CH), 2.58–2.51 (m, 2H in $CH_2$), 2.09–1.97 (m, 4H, $CH_2CH_2$), 1.84 (d, *J* = 9.0 Hz, 2H, $CH_2$), 1.45–1.38 (m, 2H, $CH_2$). **$^{13}$C NMR** (101 MHz, DMSO-$d_6$) $\delta$: 173.8, 141.2, 139.2, 139.0, 131.6, 128.5, 127.5, 127.3, 127.0, 126.8, 126.6, 126.4, 125.9, 61.8, 56.68, 54.98, 45.6, 29.3, 25.1. **HRMS (ESI)**: *m/z* calcd for $C_{48}H_{49}N_4O_6S_2^+$ $[M+H]^+$: 841.3088, found: 841.3098.

**(1*S*,2*S*)-*N*[1],*N*[2]-Bis((1*R*,2*R*)-2-(4-methylphenylsulfonamido)-1,2-diphenylethyl)cyclohexane-1,2-dicarboxamide (4c)**

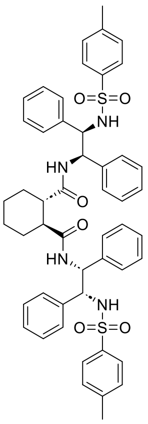


Colorless crystals, 245 mg, yield: 73%, M.p. 251.3–252.7 °C; $R_f$ = 0.23 (DCM/MeOH 20:1, *v*/*v*). $[\alpha]_D^{25}$ = –35.20 (*c* = 2.0, DMSO). **$^{1}$H NMR** (400 MHz, DMSO-$d_6$) $\delta$: 7.36 (d, *J* = 8.3 Hz, 4H, 4NH), 7.04 (d, *J* = 7.1 Hz, 6H, ArH), 7.00–6.96 (m, 6H, ArH), 6.92 (t, *J* = 7.5 Hz, 4H, ArH), 6.87–6.81 (m, 4H, ArH), 6.76 (d, *J* = 7.8 Hz, 2H, ArH), 6.73–6.67 (m, 4H, ArH), 6.25 (d, *J* = 7.7 Hz, 2H, ArH), 4.94 (dd, *J* = 9.9, 7.8 Hz, 2H, 2CH), 4.56 (dd, *J* = 9.9, 7.6 Hz, 2H, 2CH), 2.53 (d, *J* = 9.9 Hz, 2H, 2CH), 2.28 (s, 6H, $2CH_3$), 2.02 (d, *J* = 12.7 Hz, 2H in $2CH_2$), 1.81 (d, *J* = 8.7 Hz, 2H in $2CH_2$), 1.54 (m, 2H, $CH_2$), 1.39–1.28 (m, 2H, $CH_2$). **$^{13}$C NMR** (101 MHz, DMSO-$d_6$) $\delta$: 173.7, 141.7, 139.3, 139.2, 138.3, 128.9, 127.5, 127.3, 127.0, 126.7, 126.5, 126.3, 126.0, 61.7, 56.6, 45.5, 29.3, 25.1, 20.8. **HRMS (ESI)**: *m/z* calcd for $C_{50}H_{53}N_4O_6S_2^+$ $[M+H]^+$: 869.3401, found: 869.3409.

**(1*S*,2*S*)-*N*[1],*N*[2]-Bis((1*R*,2*R*)-2-(4-methoxyphenylsulfonamido)-1,2-diphenylethyl)cyclohexane-1,2-dicarboxamide (4d)**

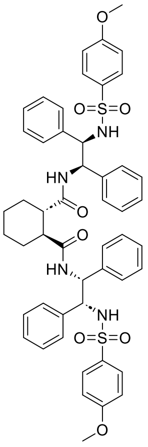

Colorless crystals, 511 mg, yield: 87%, M.p. 256.3–258.7 °C; $R_f$ = 0.20 (DCM/MeOH 20:1, *v*/*v*). $[\alpha]_D^{25}$ = –13.05 (*c* = 2.0, DMSO). **$^1$H NMR** (400 MHz, DMSO-*d*$_6$) *δ*: 7.88 (t, *J* = 11.0 Hz, 1H, 4H, 4NH), 7.18 (d, *J* = 8.5 Hz, 4H, ArH), 7.12–7.05 (m, 14H, ArH), 7.04 (d, *J* = 8.1 Hz, 2H, ArH), 6.87 (t, *J* = 7.5 Hz, 4H, ArH), 6.68 (d, *J* = 8.5 Hz, 4H, ArH), 5.05 (dd, *J* = 9.8, 4.9 Hz, 2H, CH), 4.74 (dd, *J* = 10.0, 4.8 Hz, 2H, 2CH), 3.72 (s, 6H, 2CH$_3$), 2.29 (s, 2H, 2CH), 1.61 (s, 2H in CH$_2$), 1.53 (d, *J* = 13.4 Hz, 2H in CH$_2$), 1.10 (s, 2H in CH$_2$), 0.92 (d, *J* = 12.6 Hz, 2H in CH$_2$). **$^{13}$C NMR** (101 MHz, DMSO-*d*$_6$) *δ*: 173.7, 161.5, 139.2, 132.9, 128.1, 127.5, 127.3, 127.0, 126.7, 126.5, 126.3, 113.6, 61.6, 56.7, 55.5, 45.5, 29.2, 25.1. **HRMS (ESI)**: *m*/*z* calcd for $C_{50}H_{53}N_4O_8S_2^+$ [M+H]$^+$: 901.3300, found: 901.3299.

**(1*S*,2*S*)-*N*[1],*N*[2]-Bis((1*R*,2*R*)-2-(4-nitrophenylsulfonamido)-1,2-diphenylethyl)cyclohexane-1,2-dicarboxamide (4e)**

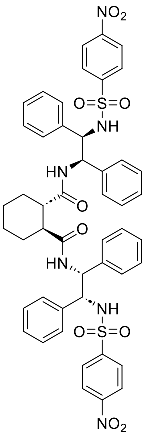

Colorless crystals, 112 mg, yield: 52%, M.p. >300 °C; $R_f$ = 0.20 (DCM/MeOH 20:1, *v*/*v*). $[\alpha]_D^{25}$ = –13.70 (*c* = 1.0, DMSO). **$^1$H NMR** (400 MHz, DMSO-*d*$_6$) *δ*: 8.47 (d, *J* = 10.0 Hz, 2H, 2NH), 8.01–7.96 (m, 4H, ArH), 7.93 (d, *J* = 9.8 Hz, 2H, 2NH), 7.53–7.47 (m, 4H, ArH), 7.12–6.95 (m, 14H, ArH), 6.91–6.78 (m, 6H, ArH), 5.08 (dd, *J* = 9.7, 5.4 Hz, 2H, 2CH), 4.77 (dd, *J* = 10.0, 5.4 Hz, 2H, CH), 2.35–2.25 (m, 2H, CH), 1.69–1.57 (m, 4H, CH$_2$CH$_2$), 1.17–1.07 (m, 2H, CH$_2$), 1.03–0.97 (m, 2H, CH$_2$). **$^{13}$C NMR** (101 MHz, DMSO-*d*$_6$) *δ:* 173.7, 148.7, 146.5, 139.0, 138.7, 127.6, 127.49, 127.45, 127.1, 126.8, 126.7, 126.4, 123.8, 62.2, 56.3, 45.6, 29.3, 25.1. **HRMS (ESI)**: *m*/*z* calcd for $C_{48}H_{47}N_6O_{10}S_2^+$ [M+H]$^+$: 931.2790, found: 931.2781.

**(1*S*,2*S*)-*N*[1],*N*[2]-Bis((1*R*,2*R*)-2-(trifluoromethylsulfonamido)-1,2-diphenylethyl)cyclohexane-1,2-dicarboxamide (4f)**

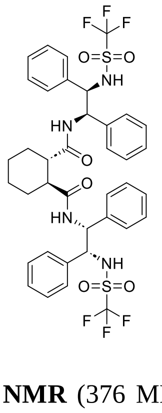

Colorless crystals, 294 mg, yield: 32%, M.p. 262.5–264.3 °C; $R_f$ = 0.23 (DCM/MeOH 20:1, *v*/*v*). $[\alpha]_D^{25}$ = –32.90 (*c* = 1.0, DMSO). **$^1$H NMR** (400 MHz, DMSO-*d*$_6$) *δ*: 9.73 (d, *J* = 9.6 Hz, 2H, 2NH), 7.96 (d, *J* = 9.8 Hz, 2H, 2NH), 7.32 (d, *J* = 7.1 Hz, 4H, ArH), 7.26 (t, *J* = 7.4 Hz, 4H, ArH), 7.20 (d, *J* = 7.0 Hz, 2H, ArH), 7.18–7.12 (m, 4H, ArH), 7.06–6.99 (m, 6H, ArH), 5.20 (dd, *J* = 9.8, 5.6 Hz, 2H, 2CH), 4.86 (dd, *J* = 9.7, 5.6 Hz, 2H, 2CH), 2.37–2.30 (m, 2H, 2CH), 1.70–1.56 (m, 4H, 2CH$_2$), 1.20–0.96 (m, 4H, 2CH$_2$). **$^{13}$C NMR** (101 MHz, DMSO-*d*$_6$) *δ*: 173.6, 138.4, 127.9, 127.6, 127.4, 127.0, 126.83, 126.75, 63.4, 56.7, 45.6, 29.2, 25.0. **$^{19}$F NMR** (376 MHz, DMSO-*d*$_6$) *δ*: –77.97. **HRMS (ESI)**: *m*/*z* calcd for $C_{38}H_{39}F_6N_4O_6S_2^+$ [M+H]$^+$: 825.2210, found: 825.2209.

**(1*S*,2*S*)-*N*[1],*N*[2]-Bis((1*S*,2*S*)-2-(4-methylphenylsulfonamido)-1,2-diphenylethyl)cyclohexane-1,2-dicarboxamide (4g)**

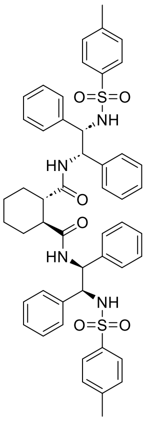

Colorless crystals, 235 mg, yield: 70%, M.p. 251.2–252.7 °C; $R_f$ = 0.23 (DCM/MeOH 20:1, *v*/*v*). $[\alpha]_D^{25}$ = –21.45 (*c* = 2.0, DMSO). **$^1$H NMR** (400 MHz, DMSO-$d_6$) $\delta$: 8.06–8.01 (m, 2H, 2NH), 7.96 (d, *J* = 8.5 Hz, 2H, 2NH), 7.25–7.17 (m, 4H, ArH), 7.16–6.94 (m, 24H, ArH), 5.02 (dd, *J* = 8.4, 6.4 Hz, 2H, 2CH), 4.66–4.58 (d, *J* = 5.1 Hz, 2H, 2CH), 2.52–2.43 (m, 2H, 2CH), 2.26 (s, 6H, $CH_3$), 1.83–1.73 (m, 2H in $2CH_2$), 1.71–1.58 (m, 2H in $2CH_2$), 1.22–1.05 (m, 4H, $2CH_2$). **$^{13}$C NMR** (101 MHz, DMSO-$d_6$) $\delta$: 173.9, 141.7, 140.4, 138.9, 138.3, 128.9, 127.6, 127.5, 127.4, 127.0, 126.6, 126.5, 126.1, 61.9, 57.6, 44.8, 29.5, 25.1, 20.9. **HRMS (ESI)**: *m*/*z* calcd for $C_{50}H_{53}N_4O_6S_2^+$ $[M+H]^+$: 869.3401, found: 869.3409.

**(1*S*,2*S*)-*N*[1],*N*[2]-Bis((1*R*,2*R*)-2-(*t*-butyloxy carbonyl)-1,2-diphenylethyl)cyclohexane-1,2-dicarboxamide (4h)**

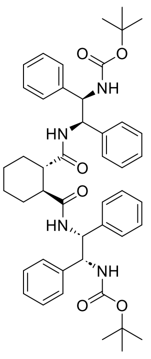

Colorless crystals, 760 mg, yield: 65%, M.p. 256.5–259.2 °C. $R_f$ = 0.32 (DCM/MeOH 20:1, *v*/*v*). $[\alpha]_D^{25}$ = –27.42 (*c* = 2.0, DMSO). **$^1$H NMR** (400 MHz, DMSO-$d_6$) $\delta$: 8.03 (d, *J* = 9.7 Hz, 2H, 2NH), 7.31–7.20 (m, 10H, 2H in 2NH & 8H in ArH), 7.19–7.13 (m, 6H, ArH), 7.01–6.90 (m, 6H, ArH), 5.23 (dd, *J* = 9.8, 4.7 Hz, 2H, 2CH), 5.11 (dd, *J* = 9.9, 4.6 Hz, 2H, 2CH), 2.29 (d, *J* = 10.2 Hz, 2H, 2CH), 1.59–1.52 (m, 2H, $CH_2$), 1.36 (d, *J* = 12.8 Hz, 2H, $CH_2$), 1.22 (s, 18H, $6CH_3$), 1.05–1.01 (m, 2H, $CH_2$), 0.90–0.78 (m, 2H, $CH_2$). **$^{13}$C NMR** (101 MHz, DMSO-$d_6$) $\delta$: 173.9, 155.0, 141.1, 139.8, 127.7, 127.4, 126.8, 126.7, 126.5, 126.2, 77.9, 57.8, 55.8, 45.5, 29.3, 28.1, 27.6, 25.1. **HRMS (ESI)**: *m*/*z* calcd for $C_{46}H_{57}N_4O_6^+$ $[M+H]^+$: 761.4273, found: 761.4276.

# 4. General procedure for the synthesis of (1*S*,2*S*)-1,2-bis((4*R*,5*R*)-1-(sulfonyl)-4,5-diphenyl-4,5-dihydro-1*H*-imidazol-2-yl)cyclohexanes 5

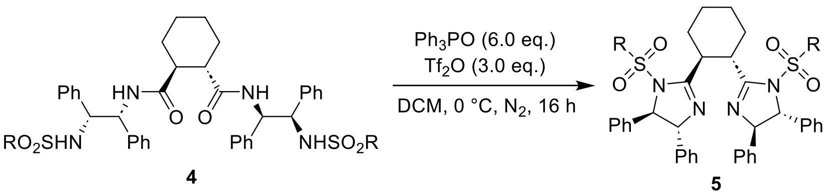


Triphenylphosphine oxide (1.67 g, 6 mmol) and triflic anhydride (0.5 mL, 3 mmol) were dissolved in dry DCM (0.5 mmol/mL) at 0 °C. The mixture was stirred at the same temperature for 30 min. The above prepared cyclohexane-1,2-dicarboxamide (1 mmol) was added portionwise. The resulting mixture was allowed to warm to room temperature and stirred at room temperature for 16 h. After addition of saturated aqueous sodium bicarbonate, the organic phase was separated and the aqueous phase was extracted with DCM (10 mL × 2). The combined organic phase was dried over sodium sulfate. After removal of the solvent, the residue was subjected to silica gel column chromatography with petroleum ether/EtOAc 10:1 (*v*/*v*) as eluent to afford colorless crystals **5**.

**(1*S*,2*S*)-1,2-Bis((4*R*,5*R*)-1-(methylsulfonyl)-4,5-diphenyl-4,5-dihydro-1*H*-imidazol-2-yl)cyclohexane (5a)**

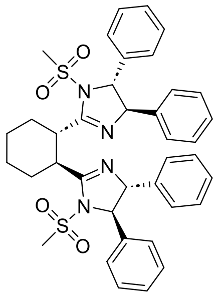

Colorless crystals, 350 mg, yield: 51%, M.p. 186.2–189.4 °C; $R_f$ = 0.15 (PE/EA 10:1, *v*/*v*). $[\alpha]_D^{25}$ = +133.15 (*c* = 2.0, $CHCl_3$). **$^1$H NMR** (400 MHz, $CDCl_3$) *δ*: 7.37 (dd, *J* = 8.1, 6.5 Hz, 4H, ArH), 7.33–7.27 (m, 5H, ArH), 7.27–7.22 (m, 7H, ArH), 7.21–7.16 (m, 4H, ArH), 4.88 (s, 4H, 4CH), 3.87–3.61 (m, 2H, 2CH), 2.87 (s, 6H, $2CH_3$), 2.73–2.65 (m, 2H, 2H in $2CH_2$), 1.98–1.92 (m, 2H, 2H in $2CH_2$), 1.61–1.53 (m, 4H, $2CH_2$). **$^{13}$C NMR** (101 MHz, $CDCl_3$) *δ*: 163.6, 142.6, 140.6, 129.2, 128.9, 128.3, 128.2, 126.9, 126.3, 78.4, 71.3, 40.9, 39.6, 33.0, 25.8. **HRMS (ESI)**: *m*/*z* calcd for $C_{38}H_{41}N_4O_4S_2^+$ [M+H]$^+$: 681.2564, found: 681.2565.

**(1*S*,2*S*)-1,2-Bis((4*R*,5*R*)-4,5-diphenyl-1-(phenylsulfonyl)-4,5-dihydro-1*H*-imidazol-2-yl)cyclohexane (5b)**

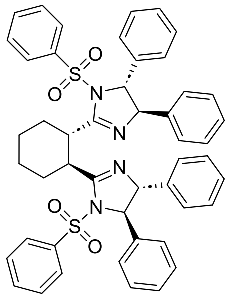

Colorless crystals, 458 mg, yield: 57%, M.p. 267.9–269.3 °C; $R_f$ = 0.25 (PE/EA 10:1, *v*/*v*). $[\alpha]_D^{25}$ = –19.60 (*c* = 2.0, $CHCl_3$). **$^1$H NMR** (400 MHz, $CDCl_3$) *δ*: 7.94–7.87 (m, 4H, ArH), 7.42–7.36 (m, 4H, ArH), 7.35–7.29 (m, 2H, ArH), 7.24–7.21 (m, 2H, ArH), 7.21–7.17 (m, 4H, ArH), 7.09 (tt, *J* = 7.5, 1.2 Hz, 2H, ArH), 7.01 (t, *J* = 7.7 Hz, 4H, ArH), 6.63–6.51 (m, 4H, ArH), 6.49–6.38 (m, 4H, ArH), 4.84 (d, *J* = 6.2 Hz, 2H, 2CH), 4.24 (d, *J* = 6.2 Hz, 2H, 2CH), 4.21–4.14 (m, 2H, 2CH), 3.04–2.90 (m, 2H, 2H in $2CH_2$), 2.10–1.94 (m, 2H, 2H in $2CH_2$), 1.64 (m, 4H, $2CH_2$). **$^{13}$C NMR** (101 MHz, $CDCl_3$) *δ*: 164.1, 142.8, 141.8, 136.6, 133.0, 129.1, 128.9, 128.6, 128.1, 127.6, 127.3, 126.2, 80.2, 71.4, 41.6, 34.7, 26.1. **HRMS (ESI)**: *m*/*z* calcd for $C_{48}H_{45}N_4O_4S_2^+$ [M+H]$^+$: 805.2877, found: 805.2874.

**(1*S*,2*S*)-1,2-Bis((4*R*,5*R*)-4,5-diphenyl-1-tosyl-4,5-dihydro-1*H*-imidazol-2-yl)cyclohexane (5c)**

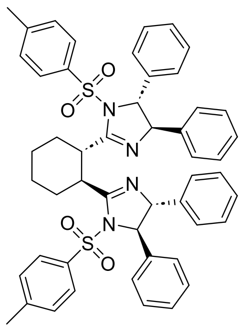

Colorless crystals, 300 mg, yield: 36%, M.p. 273.6–274.5 °C; $R_f$ = 0.25 (PE/EA 10:1, *v*/*v*). $[\alpha]_D^{25}$ = –78.22 (*c* = 0.9, $CHCl_3$). **$^1$H NMR** (400 MHz, $CDCl_3$) *δ*: 7.82 (d, *J* = 8.3 Hz, 4H, ArH), 7.42–7.35 (m, 4H, ArH), 7.35–7.24 (m, 4H, ArH), 7.21–7.15 (m, 4H, ArH), 7.01 (d, *J* = 7.7 Hz, 2H, ArH), 6.99 (d, *J* = 7.7 Hz, 2H, ArH), 6.38 (d, *J* = 8.0 Hz, 4H, ArH), 6.31 (d, *J* = 8.0 Hz, 4H, ArH), 4.83 (d, *J* = 6.1 Hz, 2H, 2CH), 4.17 (d, *J* = 6.1 Hz, 4H, 4CH), 3.08–2.56 (m, 2H, 2H in $2CH_2$), 2.04 (s, 6H, $2CH_3$), 2.02 (s, 2H, 2H in $2CH_2$), 1.73–1.58 (m, 4H, 4H in $2CH_2$). **$^{13}$C NMR** (101 MHz, $CDCl_3$) *δ*: 164.4, 143.9, 143.0, 142.1, 133.4, 129.6, 129.1, 128.9, 128.7, 128.0, 127.6, 127.5, 126.1, 80.3, 71.4, 41.6, 34.9, 26.1, 21.4. **HRMS (ESI)**: *m*/*z* calcd for $C_{50}H_{49}N_4O_4S_2^+$ [M+H]$^+$: 833.3190, found: 833.3192.

**(1*S*,2*S*)-1,2-bis((4*R*,5*R*)-1-((4-methoxyphenyl)sulfonyl)-4,5-diphenyl-4,5-dihydro-1*H*-imidazol-2-yl)cyclohexane (5d)**

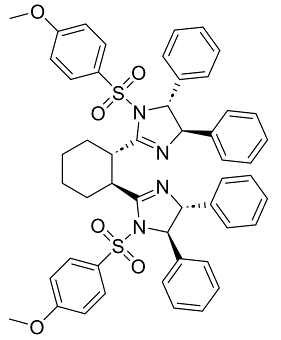

Colorless crystals, 606 mg, yield: 70%, M.p. 214.5–216.7 °C; $R_f$ = 0.13 (PE/EA 10:1, *v*/*v*). $[\alpha]_D^{25}$ = –115.45 (*c* = 2.0, $CHCl_3$). **$^1$H NMR** (400 MHz, $CDCl_3$) *δ*: 7.88–7.80 (m, 4H, ArH), 7.38 (dd, *J* = 8.1, 6.4 Hz, 4H, ArH), 7.34–7.29 (m, 2H, ArH), 7.24–7.17 (m, 6H, ArH), 6.99 (t, *J* = 7.7 Hz, 4H, ArH), 6.57–6.50 (m, 4H, ArH), 6.16–6.08 (m, 4H,ArH), 4.87 (d, *J* = 6.2 Hz, 2H, 2CH), 4.21 (d, *J* = 6.2 Hz, 2H, 2CH), 4.18–4.14 (m, 2H, 2CH), 3.51 (s, 6H, $2CH_3$), 3.03–2.93 (m, 2H, 2H in $2CH_2$), 2.00 (s, 2H, 2H in $2CH_2$), 1.72–1.60 (m, 4H, $2CH_2$). **$^{13}$C NMR** (101 MHz, $CDCl_3$) *δ*: 164.5, 163.3, 142.9, 141.9, 130.8, 129.0, 128.9, 128.5, 128.0, 127.6, 127.1, 126.3, 114.2, 80.0, 71.5, 55.7, 41.7, 34.7, 26.1. **HRMS (ESI)**: *m*/*z* calcd for $C_{50}H_{49}N_4O_6S_2^+$ $[M+H]^+$: 865.3088, found: 865.3088.

**(1*S*,2*S*)-1,2-Bis((4*R*,5*R*)-1-((4-nitrophenyl)sulfonyl)-4,5-diphenyl-4,5-dihydro-1*H*-imidazol-2-yl)cyclohexane (5e)**

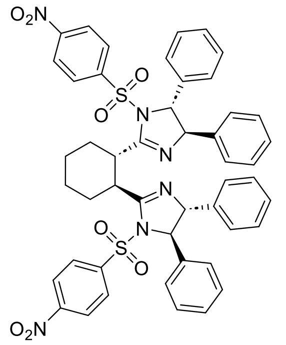

Colorless crystals, 268 mg, yield: 30%, M.p. 279.2–283.8 °C; $R_f$ = 0.30 (PE/EA 10:1, *v*/*v*). $[\alpha]_D^{25}$ = –80.00 (*c* = 1.1, $CHCl_3$). **$^1$H NMR** (400 MHz, $CDCl_3$) *δ*: 8.10–8.02 (m, 4H, ArH), 7.46–7.34 (m, 10H, ArH), 7.29–7.23 (m, 2H, ArH), 7.21–7.17 (m, 4H, ArH), 7.01 (t, *J* = 7.7 Hz, 4H, ArH), 6.54–6.47 (m, 4H, ArH), 4.91 (d, *J* = 6.3 Hz, 2H, 2CH), 4.28 (d, *J* = 6.3 Hz, 2H, 2CH), 4.16–4.09 (m, 2H, 2CH), 2.97–2.85 (m, 2H, 2H in $2CH_2$), 2.11–1.98 (m, 2H, 2H in $2CH_2$), 1.73–1.59 (m, 4H, $2CH_2$). **$^{13}$C NMR** (101 MHz, $CDCl_3$) *δ*: 163.5, 150.4, 142.2, 141.8, 140.6, 129.9, 129.3, 128.7, 128.5, 126.6, 126.2, 124.1, 79.8, 71.7, 41.7, 34.5, 26.0. **HRMS (ESI)**: *m*/*z* calcd for $C_{48}H_{43}N_6O_8S_2^+$ $[M+H]^+$: 895.2578, found: 895.2576.

**(1*S*,2*S*)-1,2-Bis((4*S*,5*S*)-4,5-diphenyl-1-tosyl-4,5-dihydro-1*H*-imidazol-2-yl)cyclohexane (5g)**

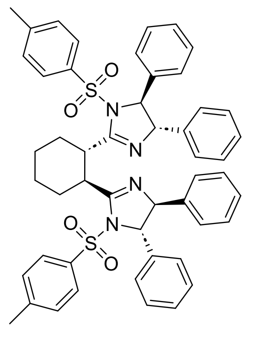

Colorless crystals, 258 mg, yield: 36%, M.p. 273.6–274.3 °C; $R_f$ = 0.25 (PE/EA 10:1, *v*/*v*). $[\alpha]_D^{25}$ = –51.33 (*c* = 2.0, $CHCl_3$). **$^1$H NMR** (400 MHz, $CDCl_3$) *δ*: 7.82 (d, *J* = 8.0 Hz, 4H, ArH), 7.46–7.23 (m, 8H, ArH), 7.19 (d, *J* = 7.3 Hz, 4H, ArH), 7.00 (t, *J* = 7.6 Hz, 4H, ArH), 6.38 (d, *J* = 7.5 Hz, 4H, ArH), 6.31 (d, *J* = 7.9 Hz, 4H, ArH), 4.82 (d, *J* = 6.1 Hz, 2H, 2CH), 4.17 (d, *J* = 6.2 Hz, 4H in 4CH), 3.03 (s, 2H in $2CH_2$), 2.04 (s, 8H, 6H in $2CH_3$ & 2H in 2CH), 1.64 (s, 4H in $2CH_2$). **$^{13}$C NMR** (101 MHz, $CDCl_3$) *δ*: 164.3, 143.9, 143.0, 142.1, 133.4, 129.6, 129.1, 128.9, 128.7, 128.0, 127.6, 127.5, 126.1, 80.3, 71.4, 41.6, 34.8, 26.1, 21.4. **HRMS (ESI)**: *m*/*z* calcd for $C_{50}H_{49}N_4O_4S_2^+$ $[M+H]^+$: 833.3190, found: 833.3192.

# 5. References


(1) Yu, F.; Sun, X.; Jin, Z.; Wen, S.; Liang, X.; Ye, J. *Chem. Commun.*, **2010**, *46*, 4589–4591.

(2) Coverdale, J. P. C.; Romero-Canelón, I.; Sanchez-Cano, C.; Clarkson, G. J.; Habtemariam, A.; Wills, M.; Sadler, P. J. *Nature Chem.*, **2018**, *10*, 347–354.

(3) Giuffredi, G. T.; Purser, S.; Sawicki, M.; Thompson, A. L.; Gouverneur, V. *Tetrahedron: Asymmetry* **2009**, *20*, 910–920.

(4) Chen, Y.-C.; Wu, T.-F.; Deng, J.-G.; Liu, H.; Cui, X.; Zhu, J.; Jiang, Y.-Z.; Choi, M. C. K.; Chan, A. S. C. *J. Org. Chem.*, **2002**, *67*, 5301–5306.

(5) Xue, D.; Chen, Y.-C.; Cui, X.; Wang, Q.-W.; Zhu, J.; Deng, J.-G. *J. Org. Chem.*, **2005**, *70*, 3584–3591.

(6) Nie, W.; Wan, Q.; Sun, J.; Chen, M.; Gao, M.; Chen, S. *Nature Commun.*, **2023**, *14*, 6671.

(7) Li, W.; Wu, W.; Yu, F.; Huang, H.; Liang, X.; Ye, J. *Org. Biomol. Chem.* **2011**, *9*, 2505–2511.

(8) Chen, J.-B.; Peng, C.; Zhou, S.-S.; Wang, Y.; Wang, Z.; Wang, X.-W. *Org. Chem. Front.*, **2022**, *9*, 3976–3989.

(9) Kochetkov, S. V.; Kucherenko, A. S.; Zlotin, S. G. *Org. Biomol. Chem.* **2018**, *16*, 6423–6429.

# 6. Copies of $^{1}$H and $^{13}$C NMR spectra of compounds 2 and 4 and HRMS spectra of products 4 and 5

**$^{1}$H NMR of *N*-[(1*R*,2*R*)-2-amino-1,2-diphenylethyl]methanesulfonamide (2a) (400 MHz, CDCl₃)**

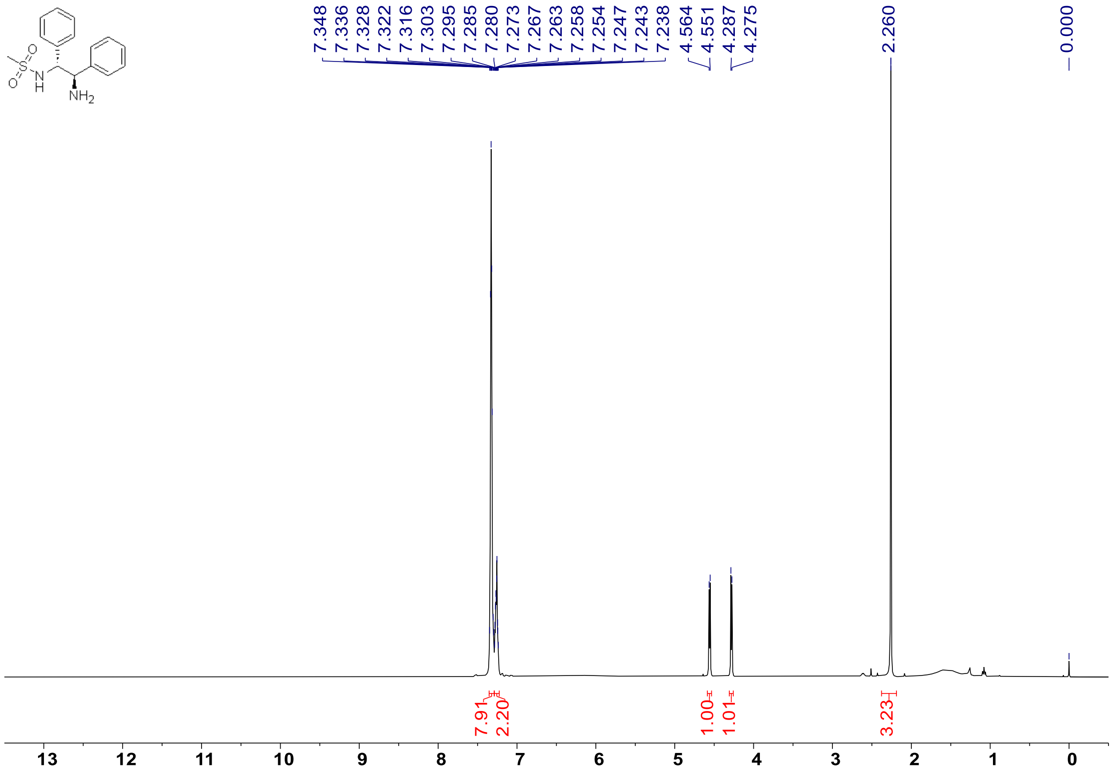


**$^{13}$C NMR of *N*-[(1*R*,2*R*)-2-amino-1,2-diphenylethyl]methanesulfonamide (2a) (101 MHz, CDCl₃)**

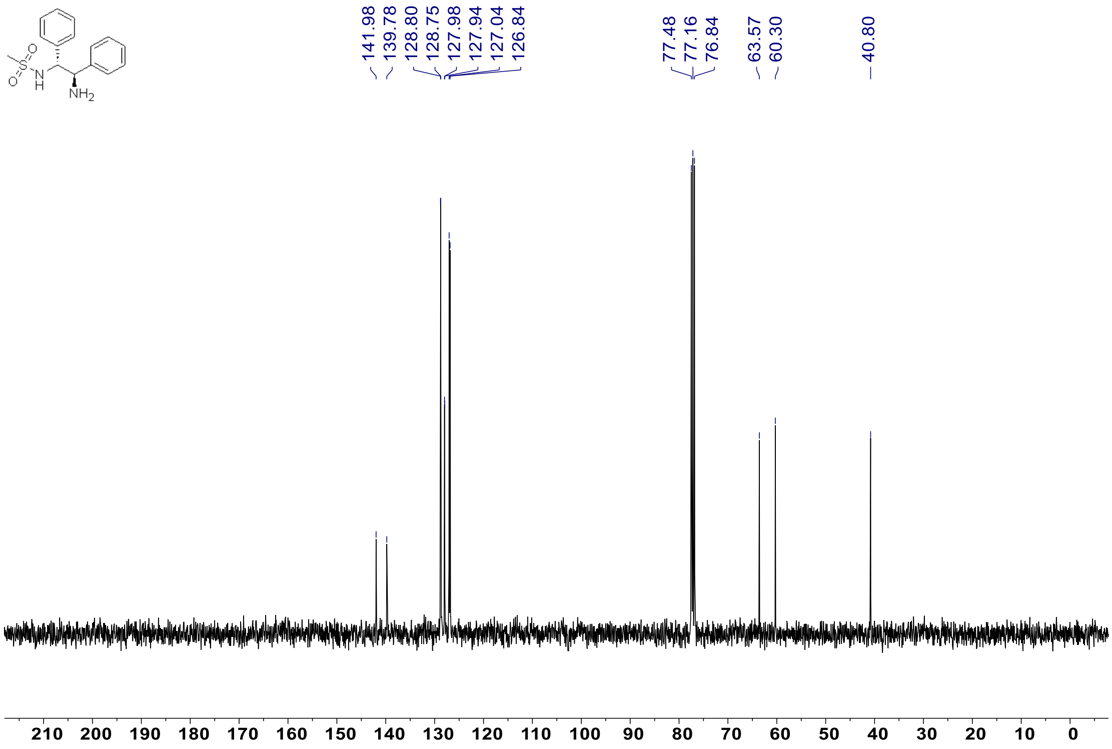

$^{1}$H NMR of *N*-[(1*R*,2*R*)-2-amino-1,2-diphenylethyl]benzenesulfonamide (2b) (400 MHz, $CDCl_3$)

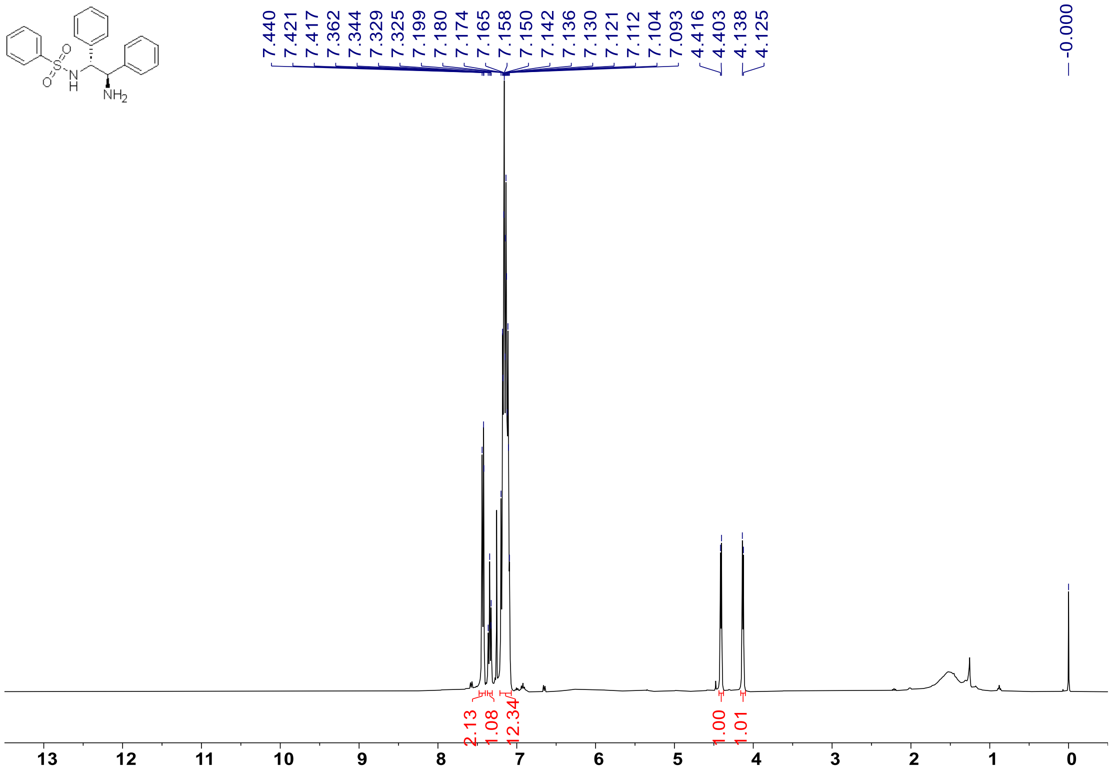

$^{13}$C NMR of *N*-[(1*R*,2*R*)-2-amino-1,2-diphenylethyl]benzenesulfonamide (2b) (101 MHz, $CDCl_3$)

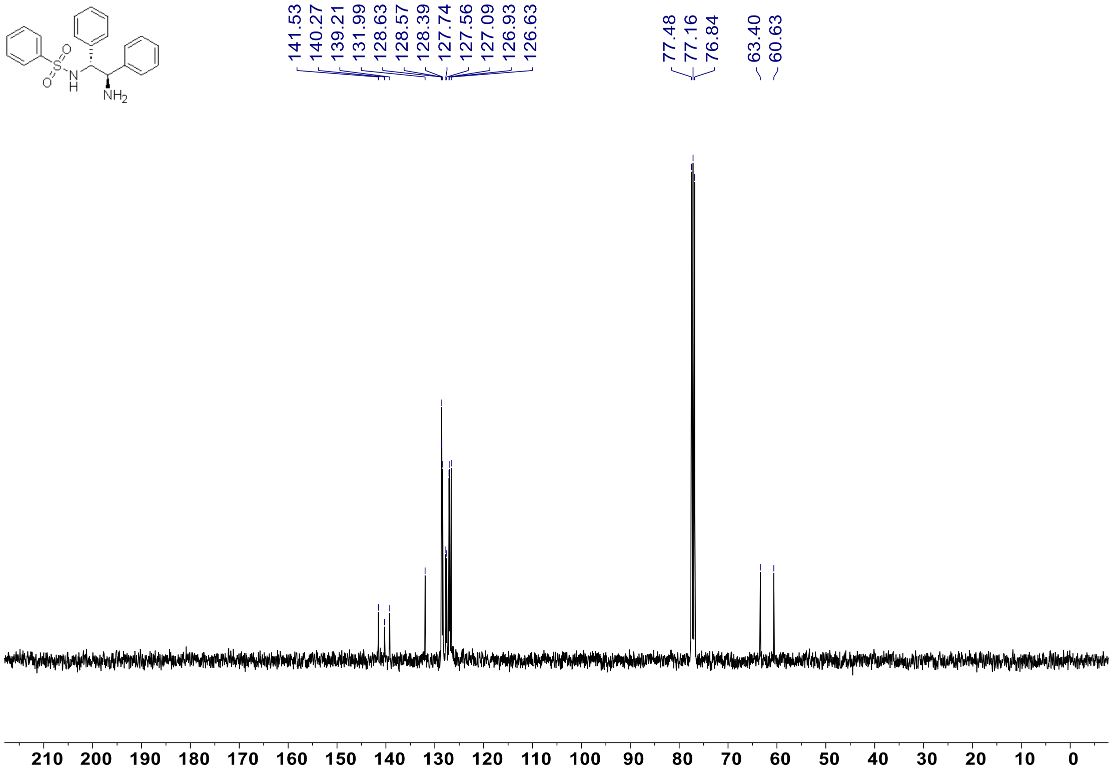

**$^{1}H$ NMR of (1*R*,2*R*)-*N*-*p*-toluenesulfonyl-1,2-diphenylethylenediamine (2c) (400 MHz, $CDCl_3$)**

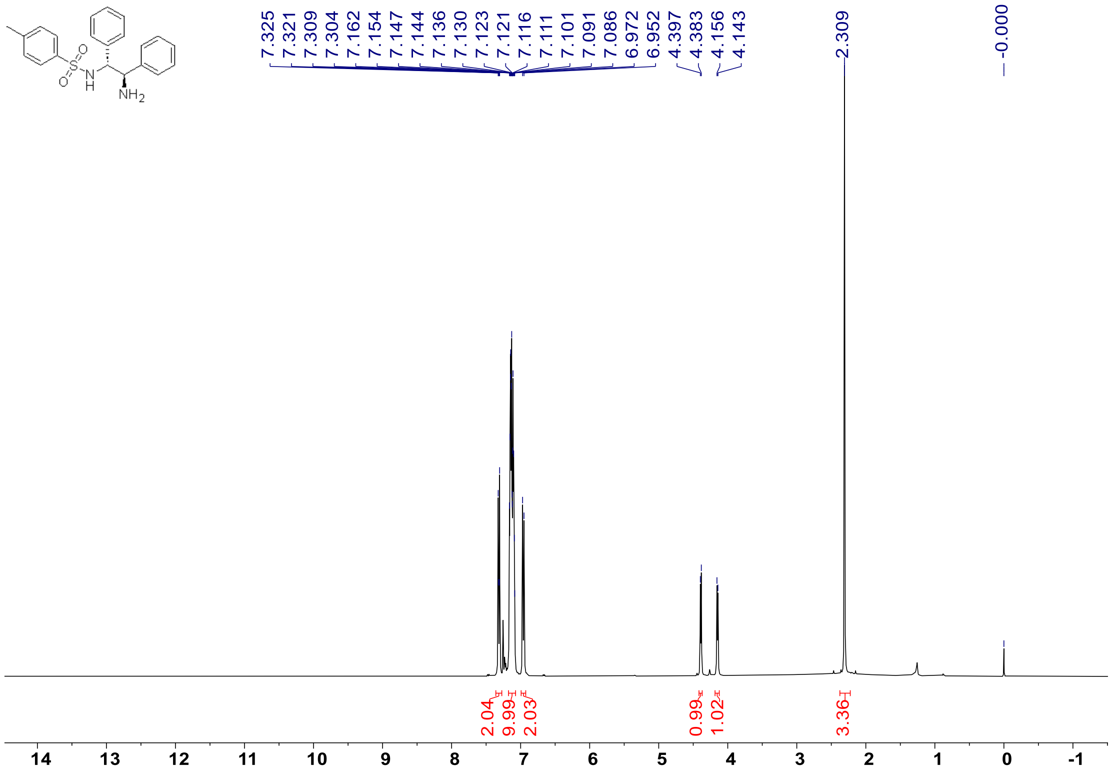


**$^{13}C$ NMR of (1*R*,2*R*)-*N*-*p*-toluenesulfonyl-1,2-diphenylethylenediamine (2c) (101 MHz, $CDCl_3$)**

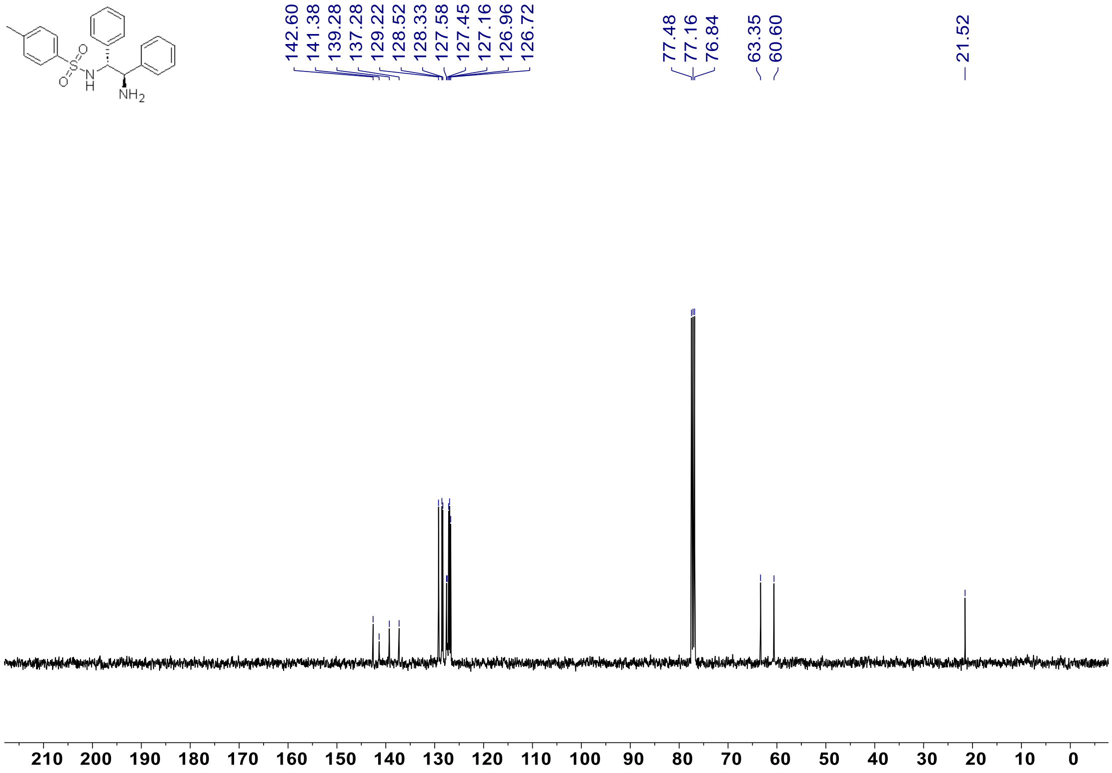

**$^{1}$H NMR of *N*-[(1*R*,2*R*)-2-amino-1,2-diphenylethyl]-4-methoxybenzenesulfonamide (2d) (400 MHz, $CDCl_3$)**

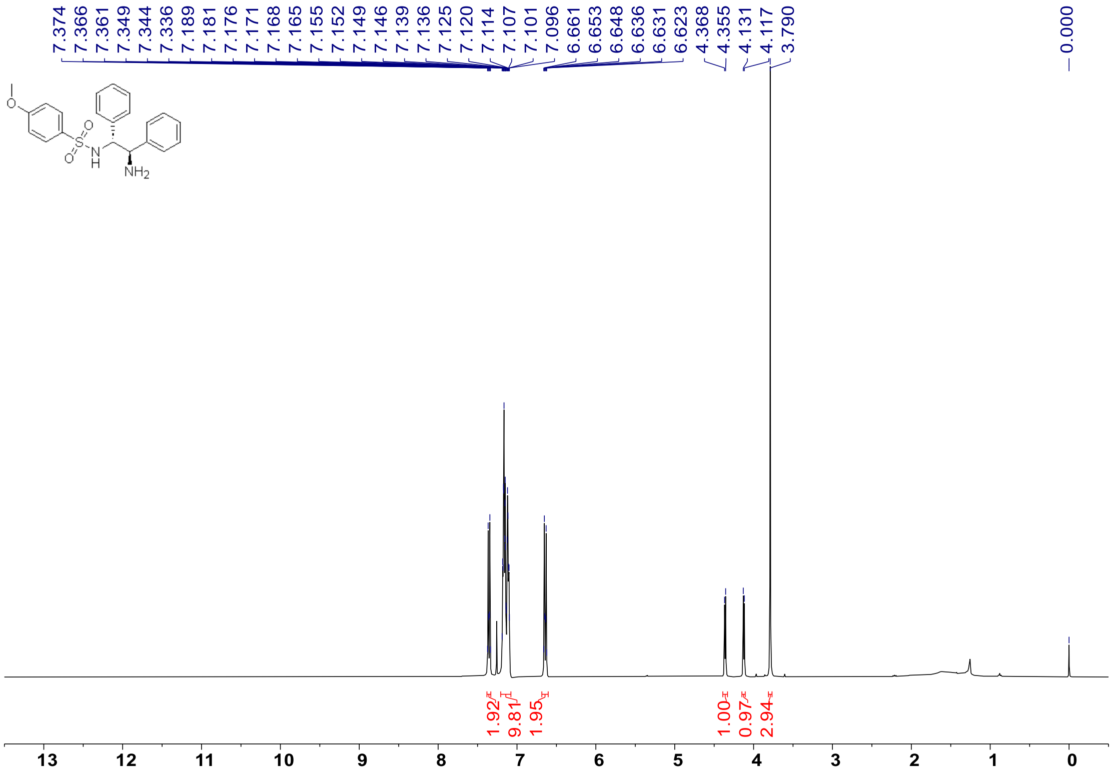

**$^{13}$C NMR of *N*-[(1*R*,2*R*)-2-amino-1,2-diphenylethyl]-4-methoxybenzenesulfonamide (2d) (101 MHz, $CDCl_3$)**

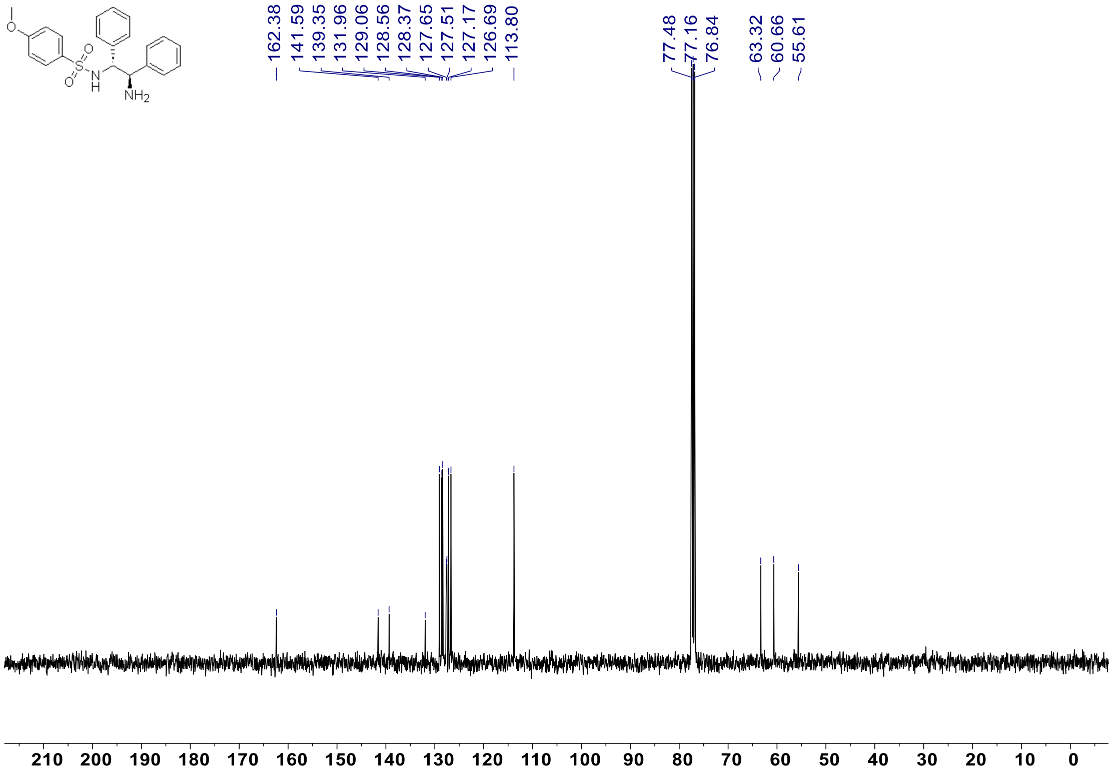

$^{1}$H NMR of *N*-[(1*R*,2*R*)-2-amino-1,2-diphenylethyl]-4-nitrobenzenesulfonamide (2e) (400 MHz, $CDCl_3$)

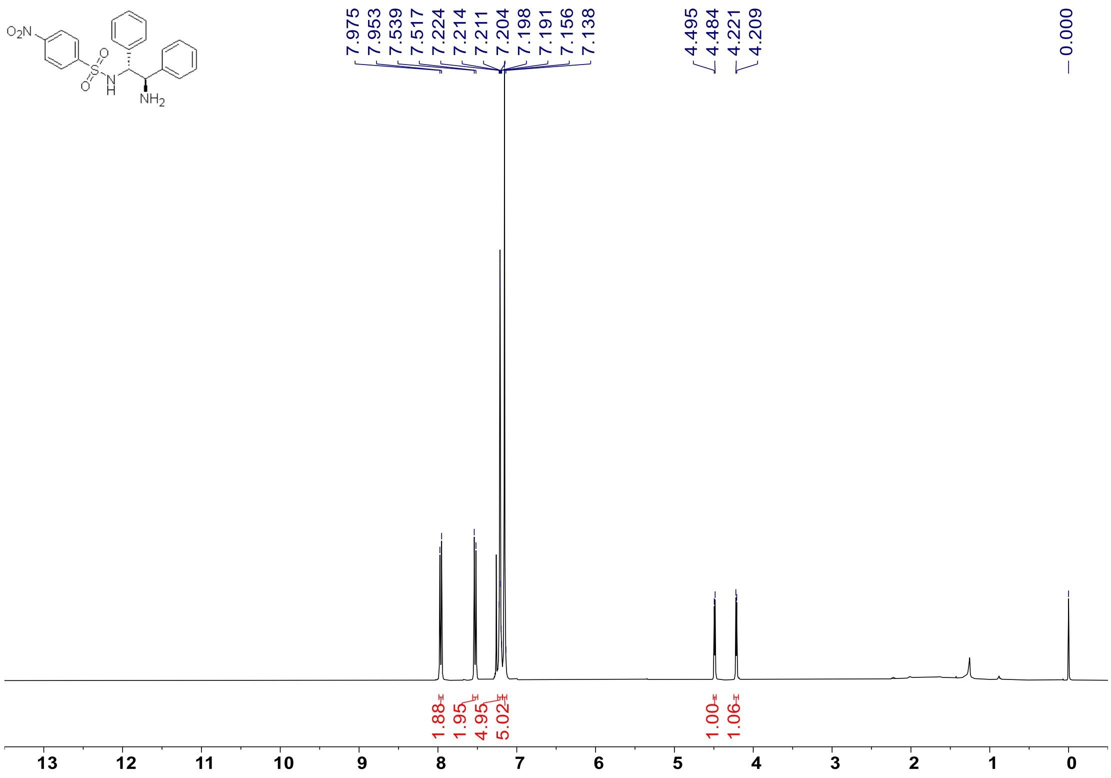


$^{13}$C NMR of *N*-[(1*R*,2*R*)-2-amino-1,2-diphenylethyl]-4-nitrobenzenesulfonamide (2e) (101 MHz, DMSO-*d*$_6$)

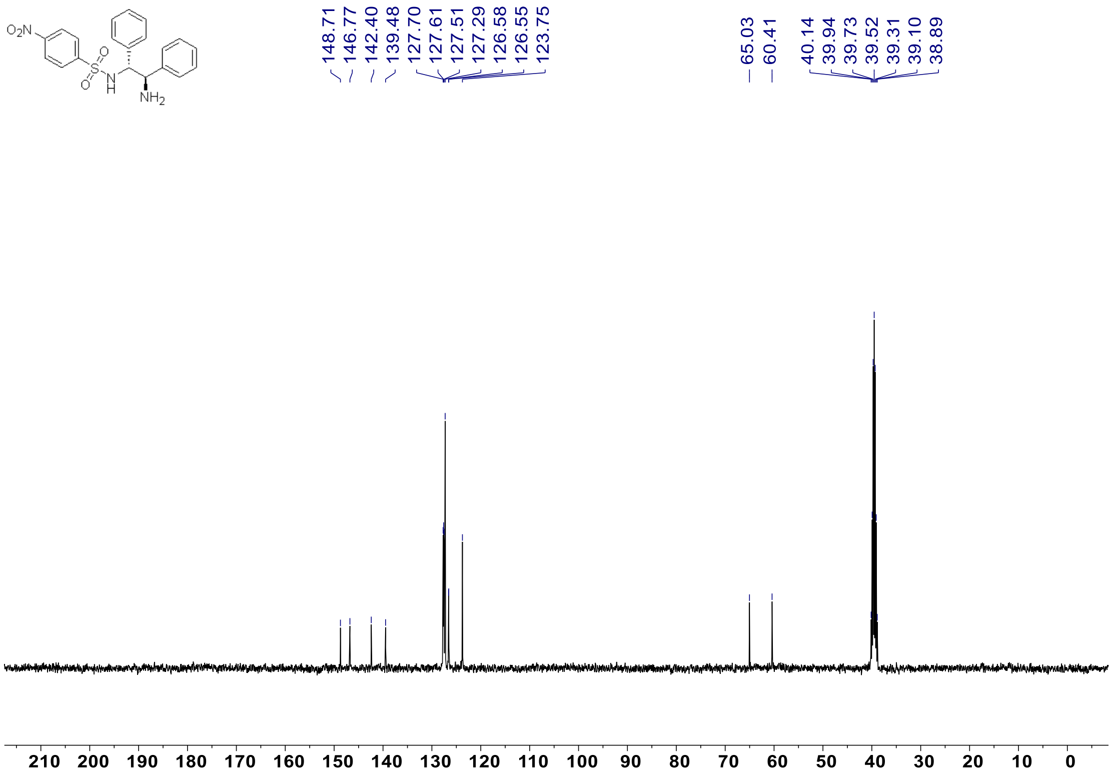

**$^{1}$H NMR of *N*-[(1*R*,2*R*)-2-amino-1,2-diphenylethyl]-1,1,1-trifluoromethanesulfonamide (2f) (400 MHz, $CDCl_3$)**

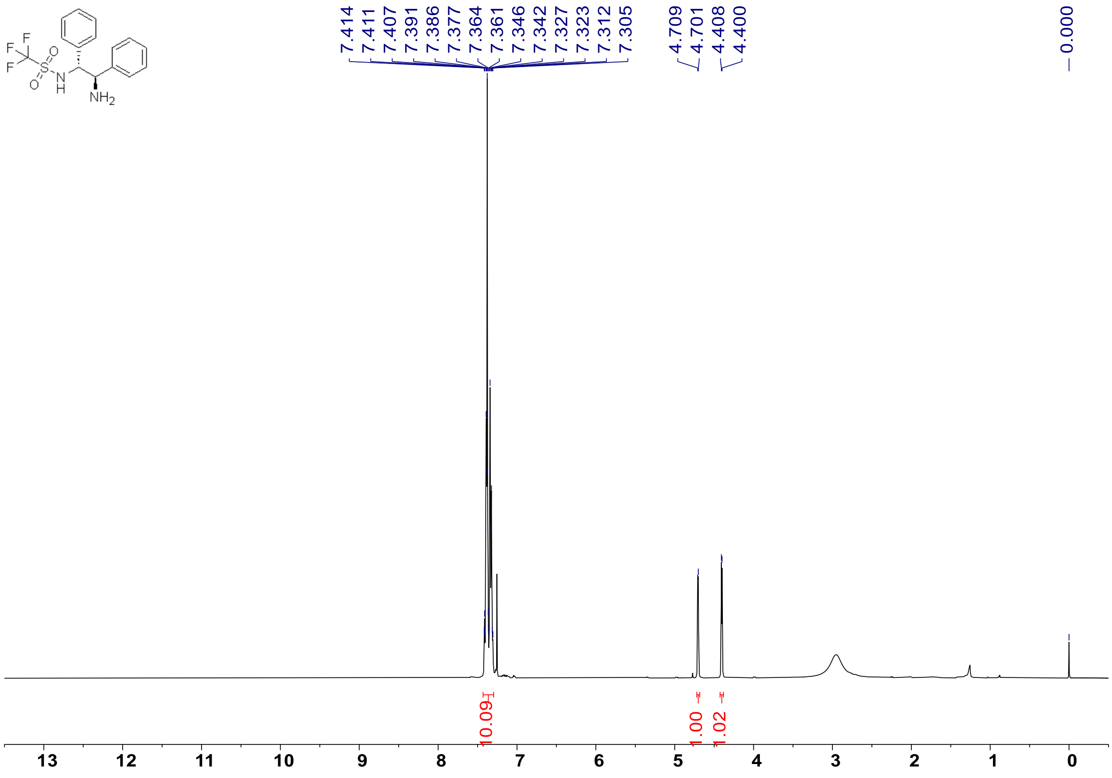


**$^{13}$C NMR of *N*-[(1*R*,2*R*)-2-amino-1,2-diphenylethyl]-1,1,1-trifluoromethanesulfonamide (2f) (101 MHz, $CDCl_3$)**

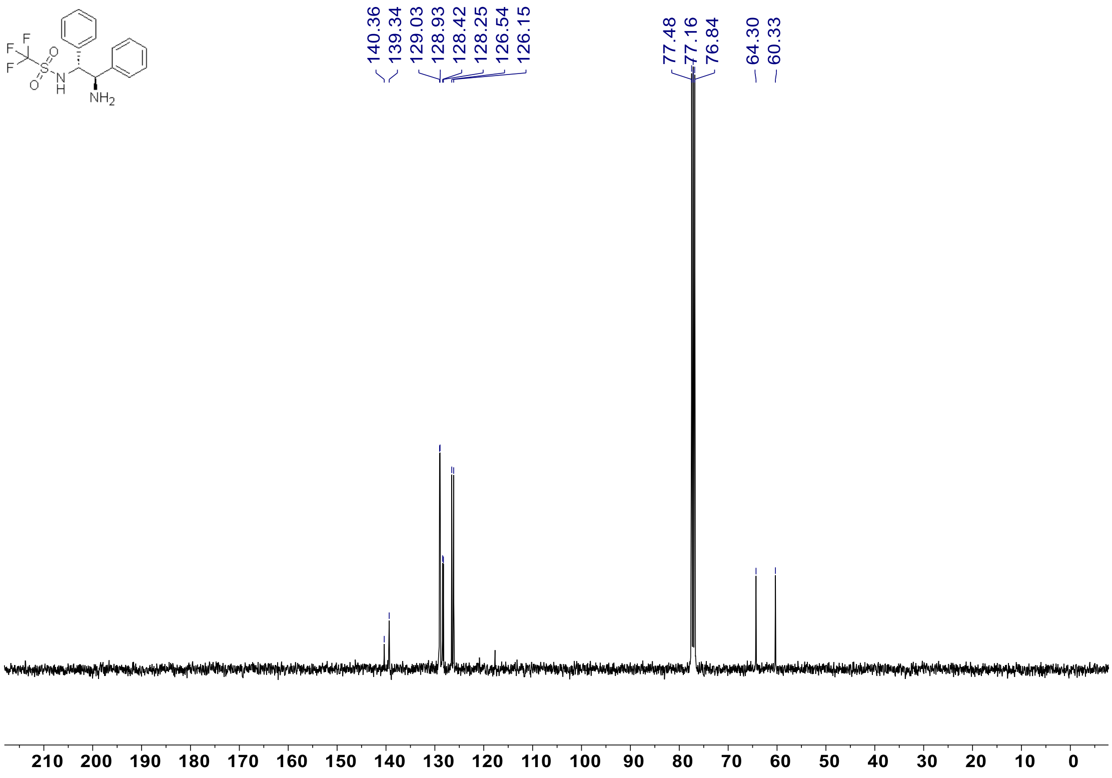

**$^{19}$F NMR of *N*-[(1*R*,2*R*)-2-amino-1,2-diphenylethyl]-1,1,1-trifluoromethanesulfonamide (2f) (376 MHz, $CDCl_3$)**

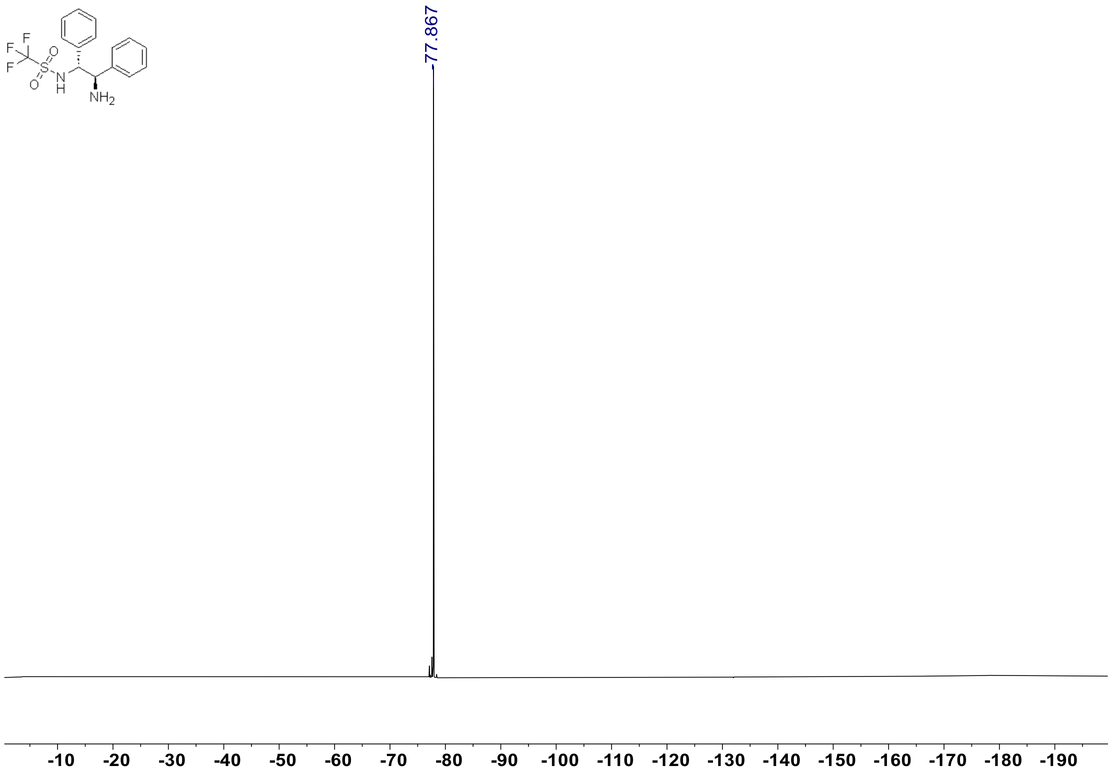

**$^{1}$H NMR of (1*S*,2*S*)-*N*-*p*-toluenesulfonyl-1,2-diphenylethylenediamine (2g) (400 MHz, CDCl$_3$)**

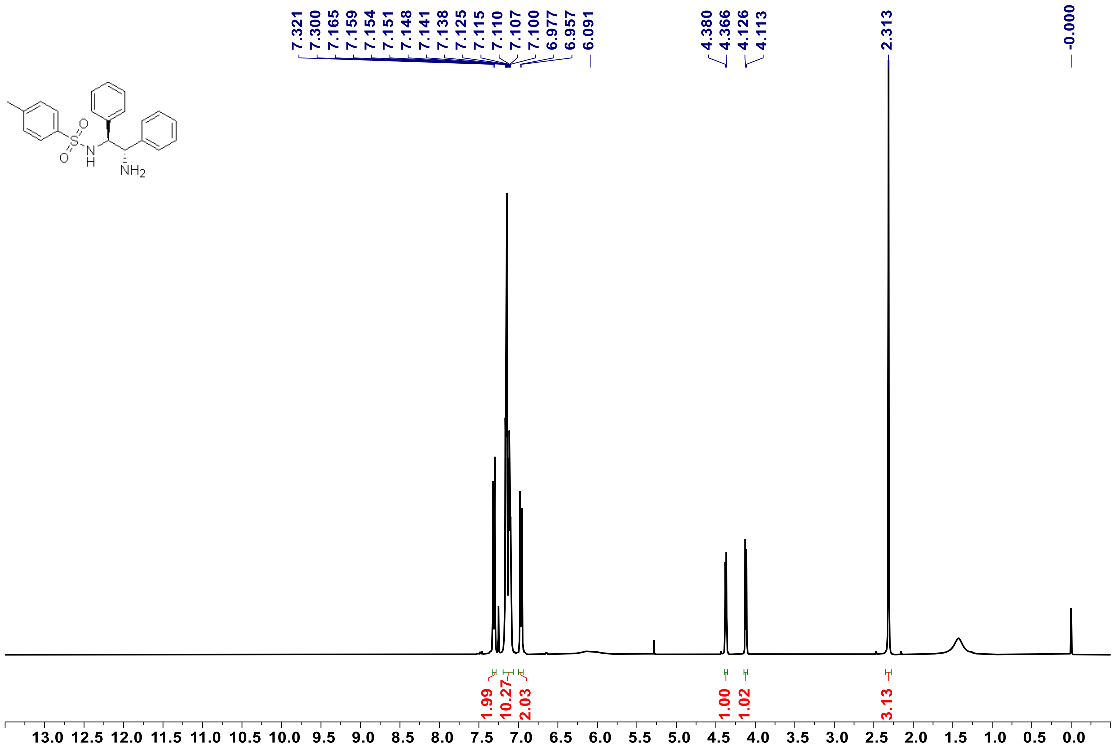


**$^{13}$C NMR of (1*S*,2*S*)-*N*-*p*-toluenesulfonyl-1,2-diphenylethylenediamine (2g) (101 MHz, CDCl$_3$)**

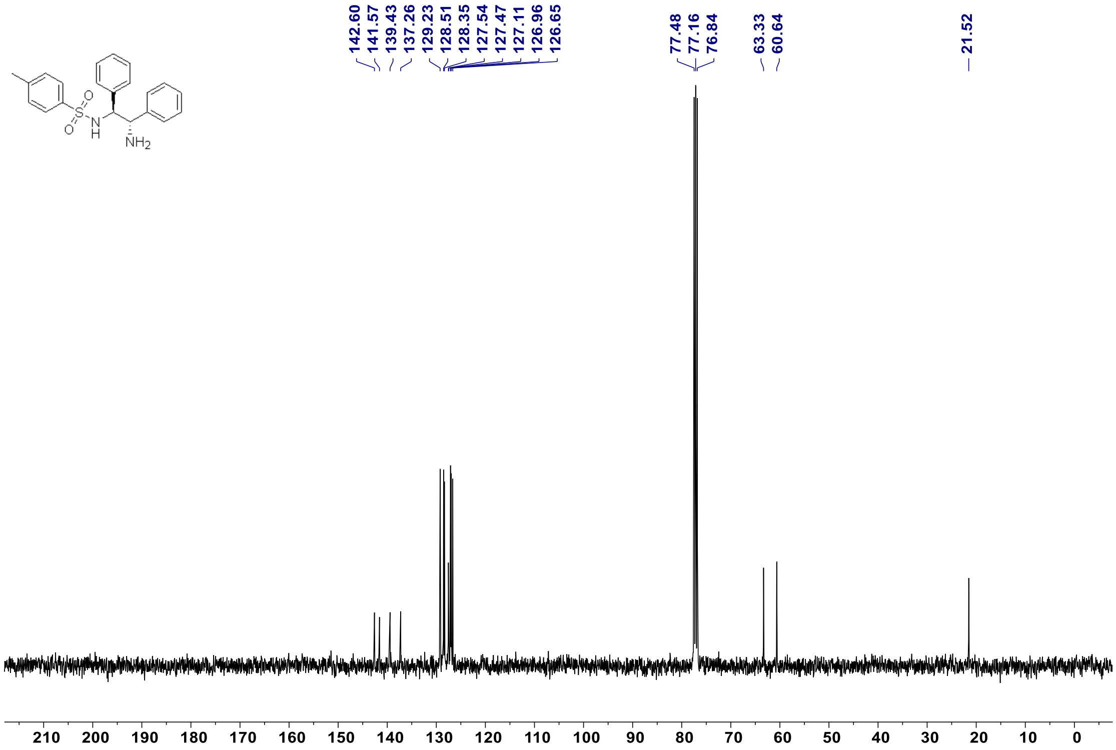

**$^{1}H$ NMR of 1,1-dimethylethyl *N*-[(1*R*,2*R*)-2-amino-1,2-diphenylethyl]carbamate (2h) (400 MHz, $CDCl_3$)**

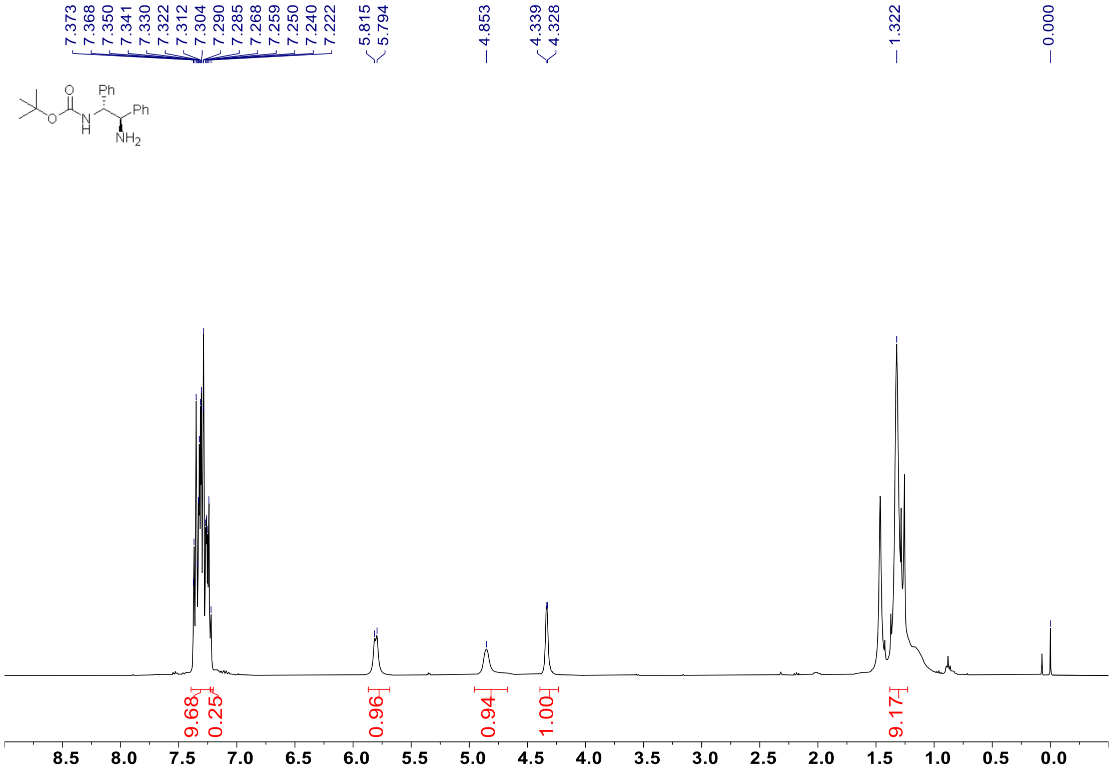


**$^{13}C$ NMR of 1,1-dimethylethyl *N*-[(1*R*,2*R*)-2-amino-1,2-diphenylethyl]carbamate (2h) (101 MHz, $CDCl_3$)**

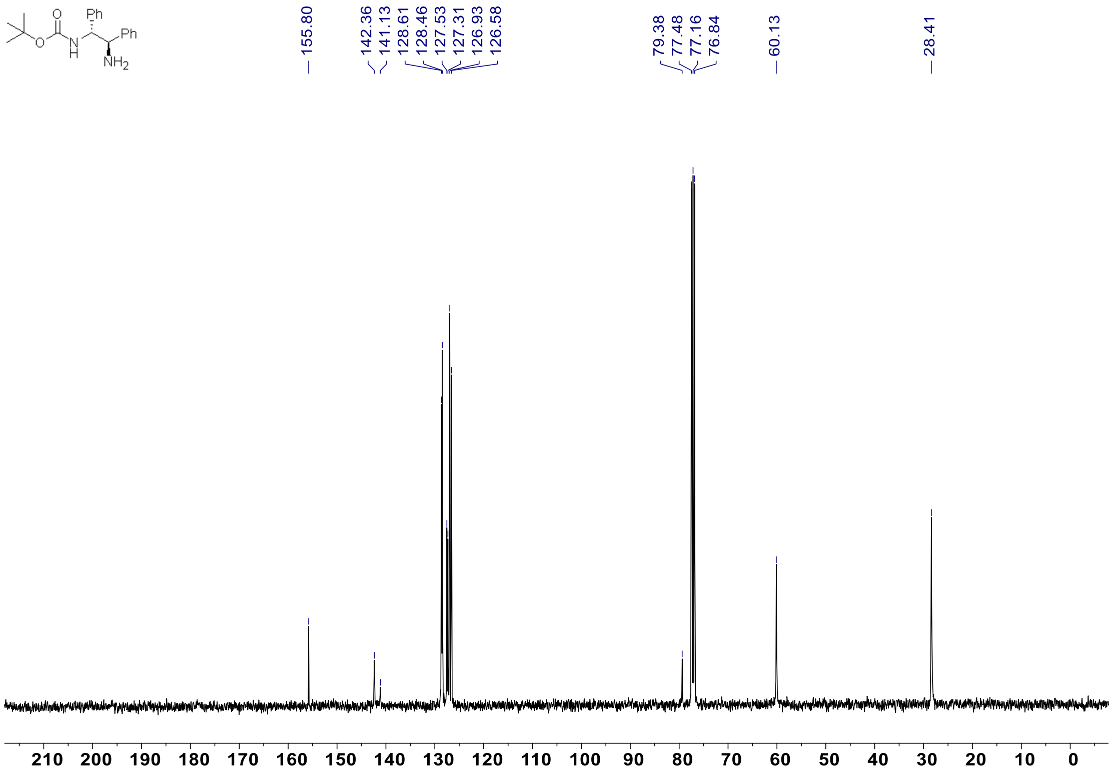

$^{1}H$ NMR of (1*S*,2*S*)-*N*[1],*N*[2]-bis((1*R*,2*R*)-2-(methylsulfonamido)-1,2-diphenylethyl)cyclohexane-1,2-dicarboxamide (4a) (400 MHz, DMSO-$d_6$)

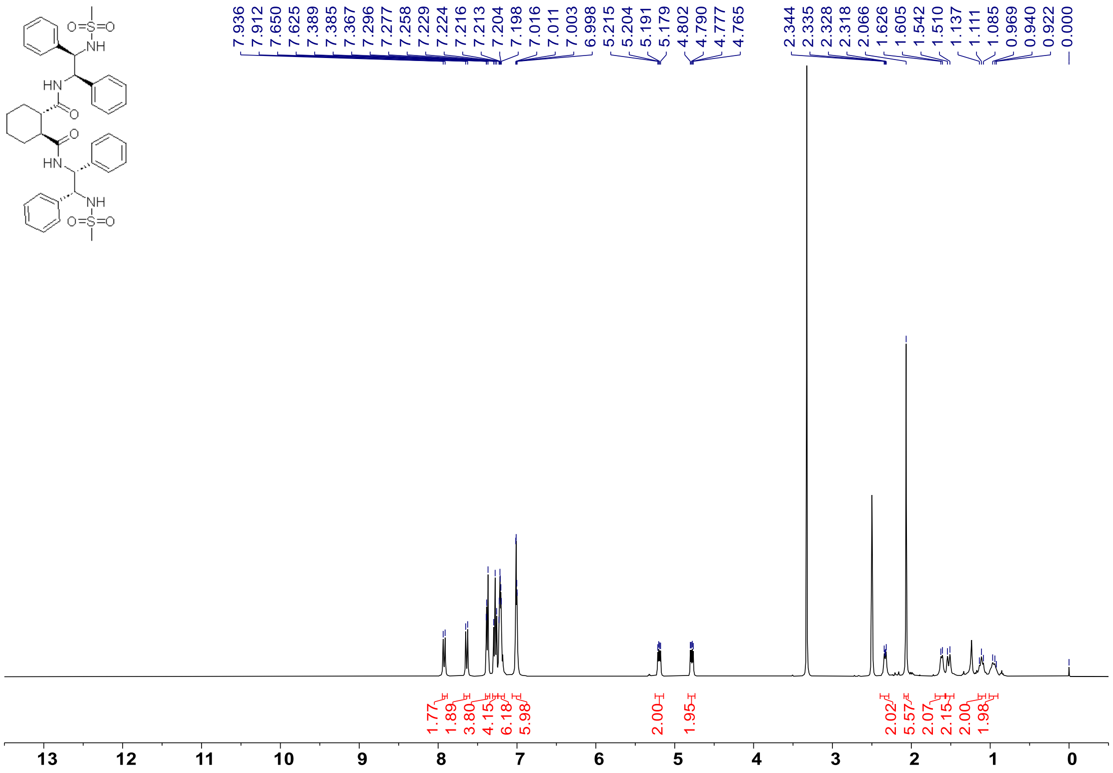


$^{13}C$ NMR of (1*S*,2*S*)-*N*[1],*N*[2]-bis((1*R*,2*R*)-2-(methylsulfonamido)-1,2-diphenylethyl)cyclohexane-1,2-dicarboxamide (4a) (101 MHz, DMSO-$d_6$)

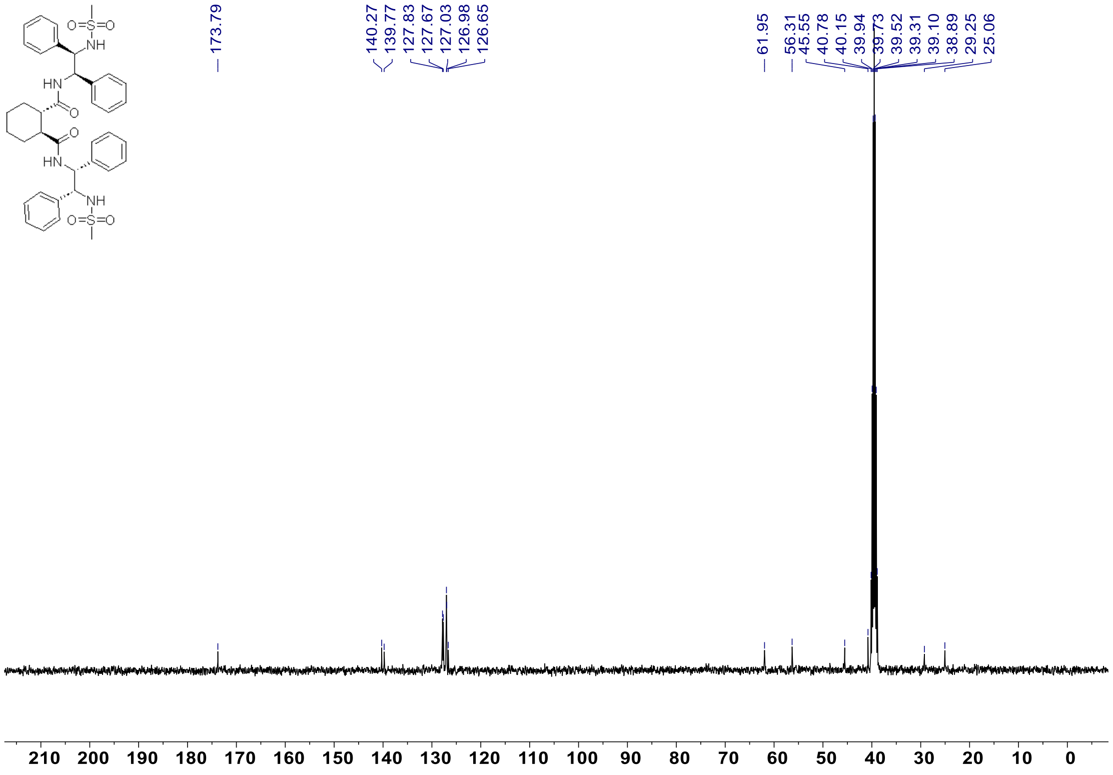

**HRMS (ESI) of (1*S*,2*S*)-*N*[1],*N*[2]-bis((1*R*,2*R*)-2-(methylsulfonamido)-1,2-diphenylethyl)cyclohexane-1,2-dicarboxamide (4a)**

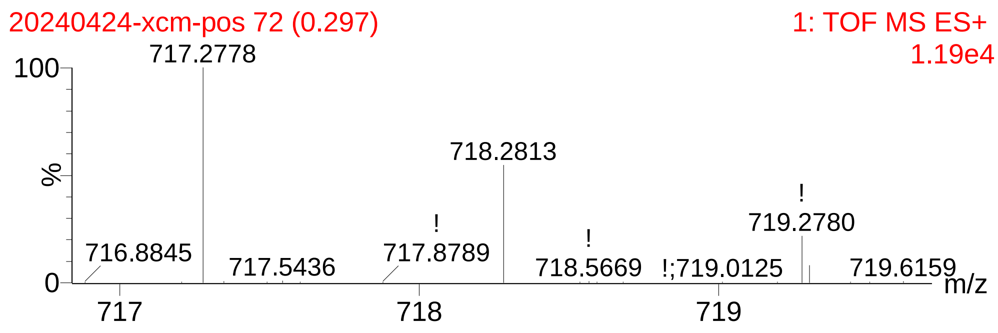

**$^{1}$H NMR of (1*S*,2*S*)-*N*$^{1}$,*N*$^{2}$-bis((1*R*,2*R*)-2-(phenylsulfonamido)-1,2-diphenylethyl)cyclohexane-1,2-dicarboxamide (4b) (400 MHz, DMSO-*d*$_6$)**

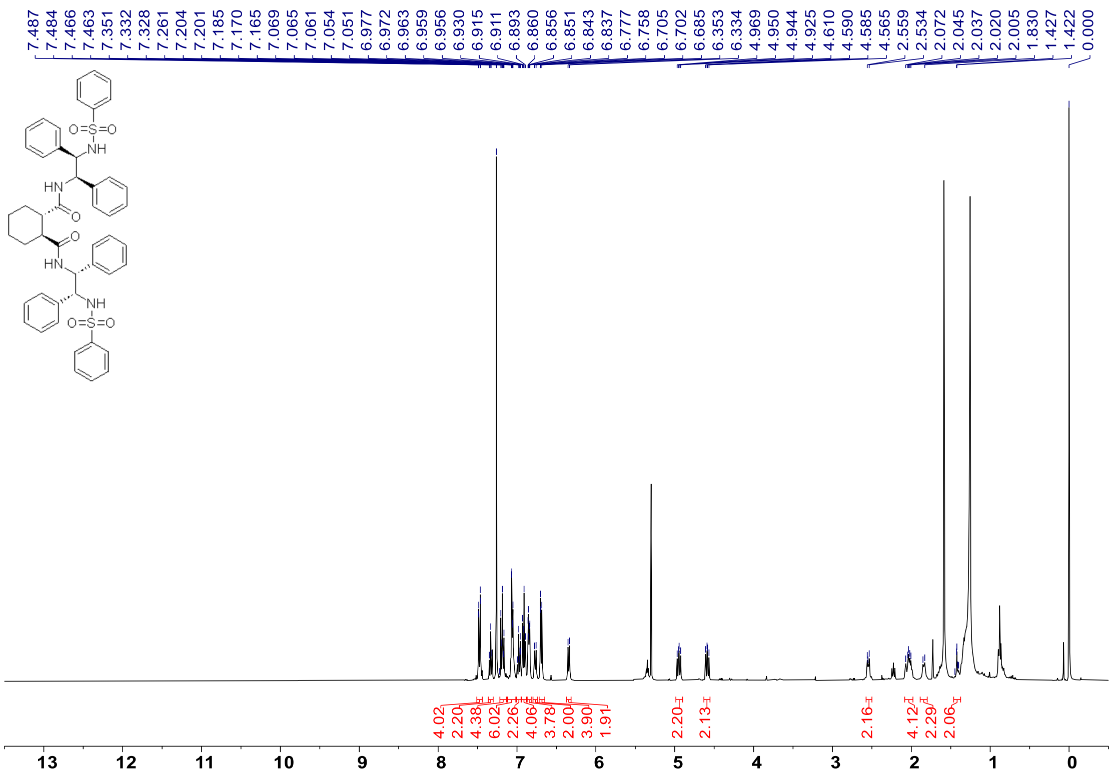


**$^{13}$C NMR of (1*S*,2*S*)-*N*$^{1}$,*N*$^{2}$-bis((1*R*,2*R*)-2-(phenylsulfonamido)-1,2-diphenylethyl)cyclohexane-1,2-dicarboxamide (4b) (101 MHz, DMSO-*d*$_6$)**

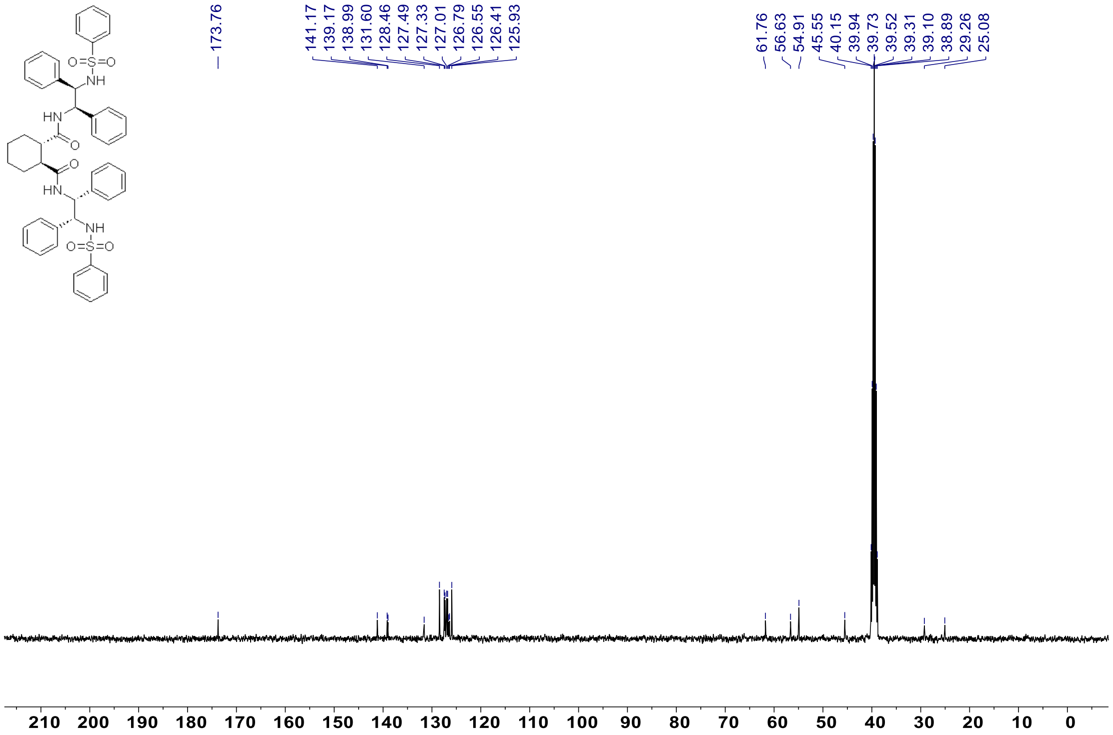

**HRMS (ESI) of (1*S*,2*S*)-*N*[1],*N*[2]-Bis((1*R*,2*R*)-2-(phenylsulfonamido)-1,2-diphenylethyl)cyclohexane-1,2-dicarboxamide (4b)**

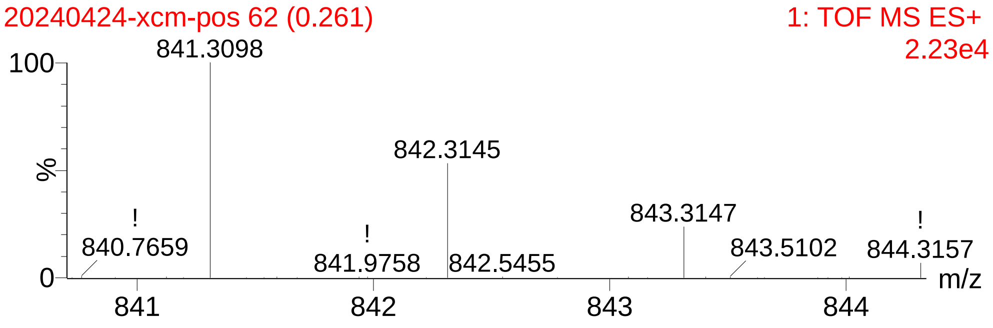

$^{1}$H NMR of (1*S*,2*S*)-*N*[1],*N*[2]-bis((1*R*,2*R*)-2-(4-methylphenylsulfonamido)-1,2-diphenylethyl)cyclohexane-1,2-dicarboxamide (4c) (400 MHz, DMSO-$d_6$)

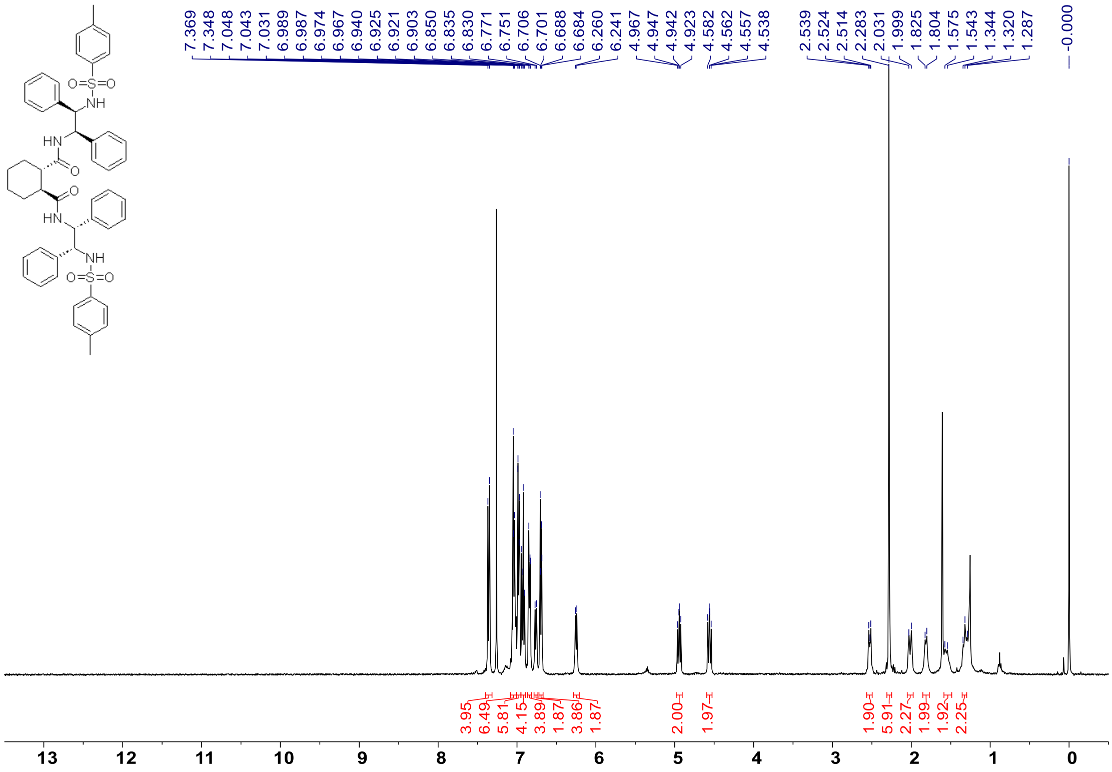


$^{13}$C NMR of (1*S*,2*S*)-*N*[1],*N*[2]-bis((1*R*,2*R*)-2-(4-methylphenylsulfonamido)-1,2-diphenylethyl)cyclohexane-1,2-dicarboxamide (4c) (101 MHz, DMSO-$d_6$)

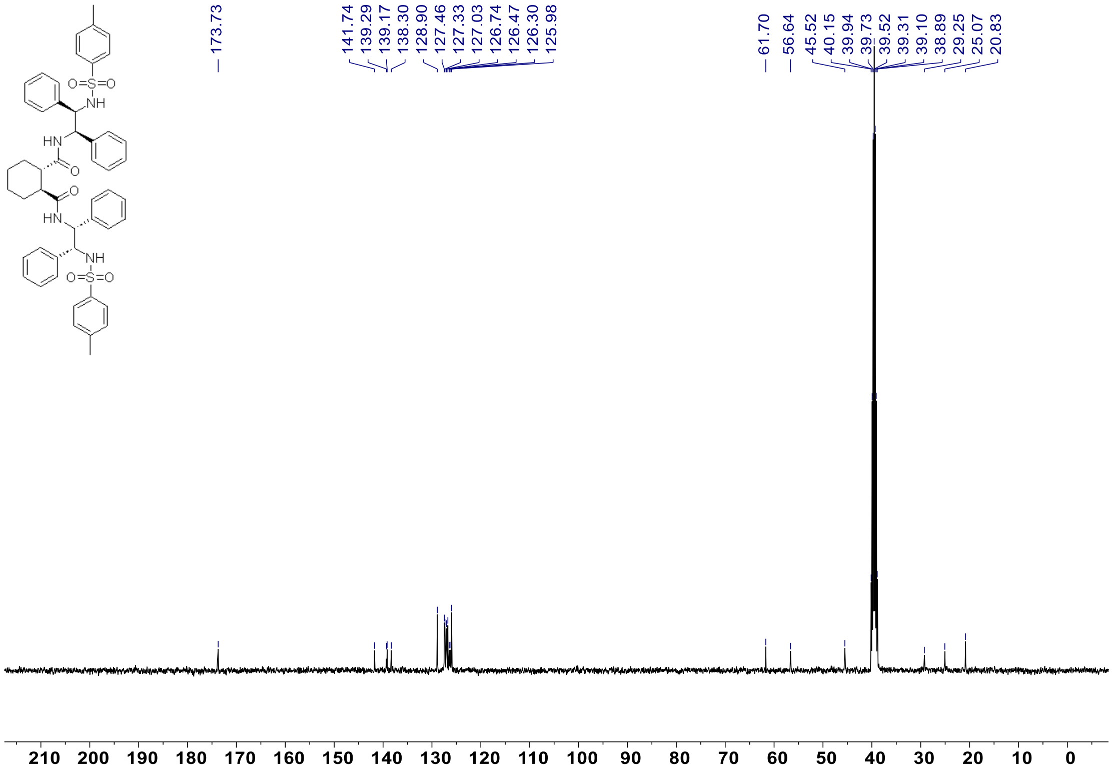

**HRMS (ESI) of (1*S*,2*S*)-*N*[1],*N*[2]-bis((1*R*,2*R*)-2-(4-methylphenylsulfonamido)-1,2-diphenylethyl)cyclohexane-1,2-dicarboxamide (4c)**

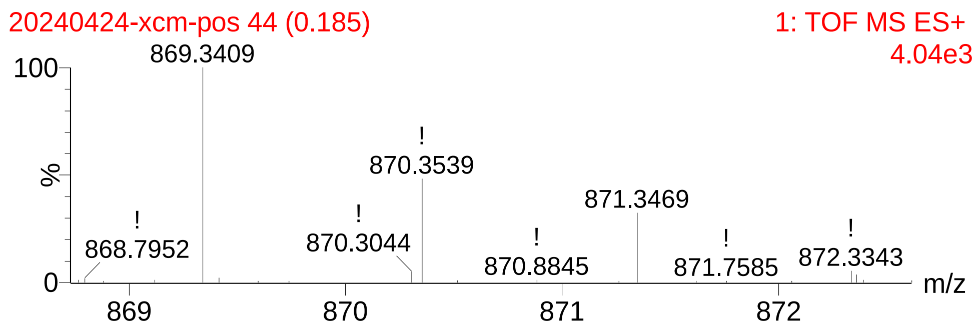

**$^{1}$H NMR of (1*S*,2*S*)-*N*$^{1}$,*N*$^{2}$-bis((1*R*,2*R*)-2-(4-methoxyphenylsulfonamido)-1,2-diphenylethyl)cyclohexane-1,2-dicarboxamide (4d) (400 MHz, DMSO-*d*$_6$)**

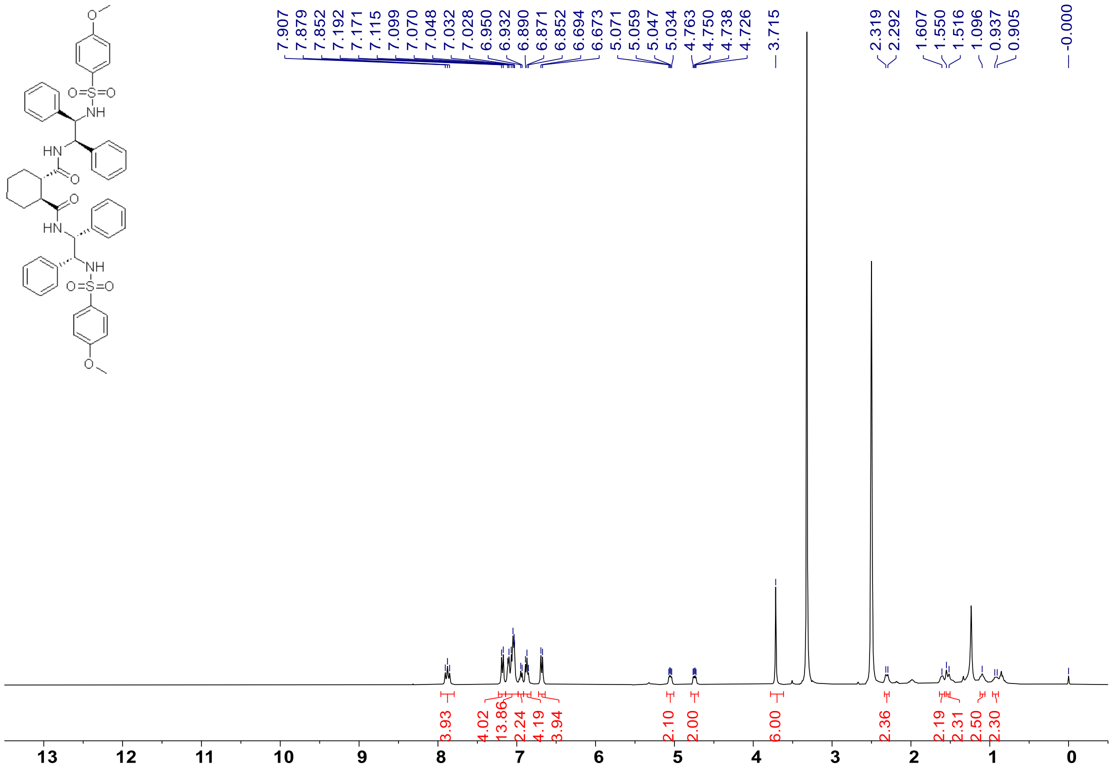


**$^{13}$C NMR of (1*S*,2*S*)-*N*$^{1}$,*N*$^{2}$-bis((1*R*,2*R*)-2-(4-methoxyphenylsulfonamido)-1,2-diphenylethyl)cyclohexane-1,2-dicarboxamide (4d) (101 MHz, DMSO-*d*$_6$)**

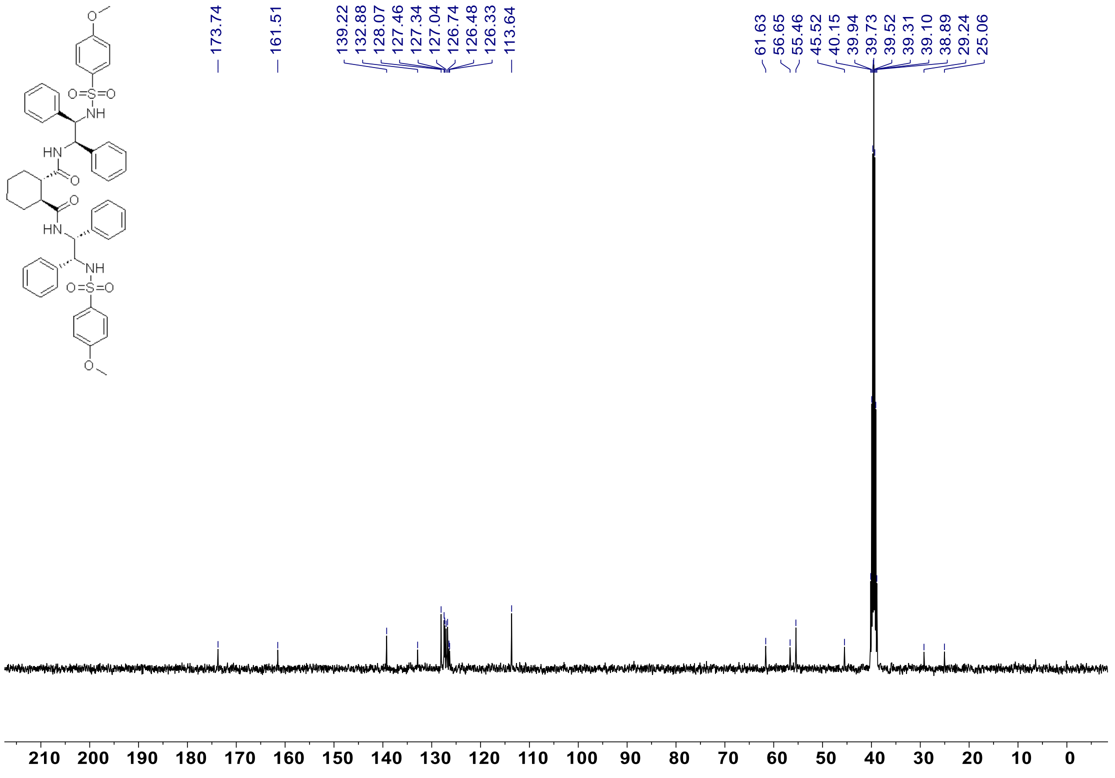

**HRMS (ESI) of (1*S*,2*S*)-*N*[1],*N*[2]-bis((1*R*,2*R*)-2-(4-methoxyphenylsulfonamido)-1,2-diphenylethyl)cyclohexane-1,2-dicarboxamide (4d)**

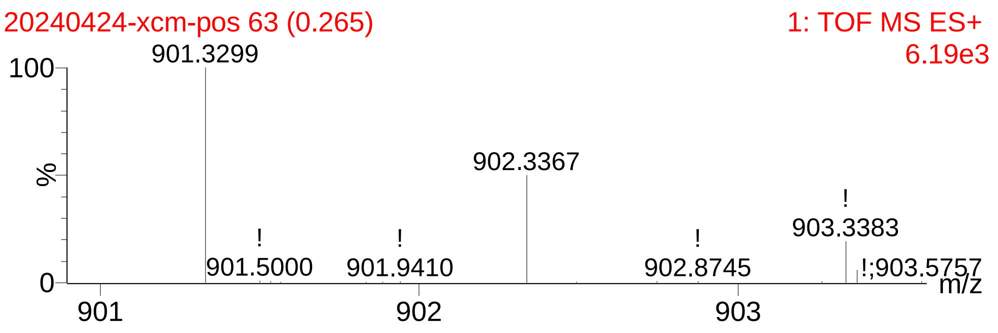

$^{1}$H NMR of (1*S*,2*S*)-*N*[1],*N*[2]-bis((1*R*,2*R*)-2-(4-nitrophenylsulfonamido)-1,2-diphenylethyl)cyclohexane-1,2-dicarboxamide (4e) (400 MHz, DMSO-$d_6$)

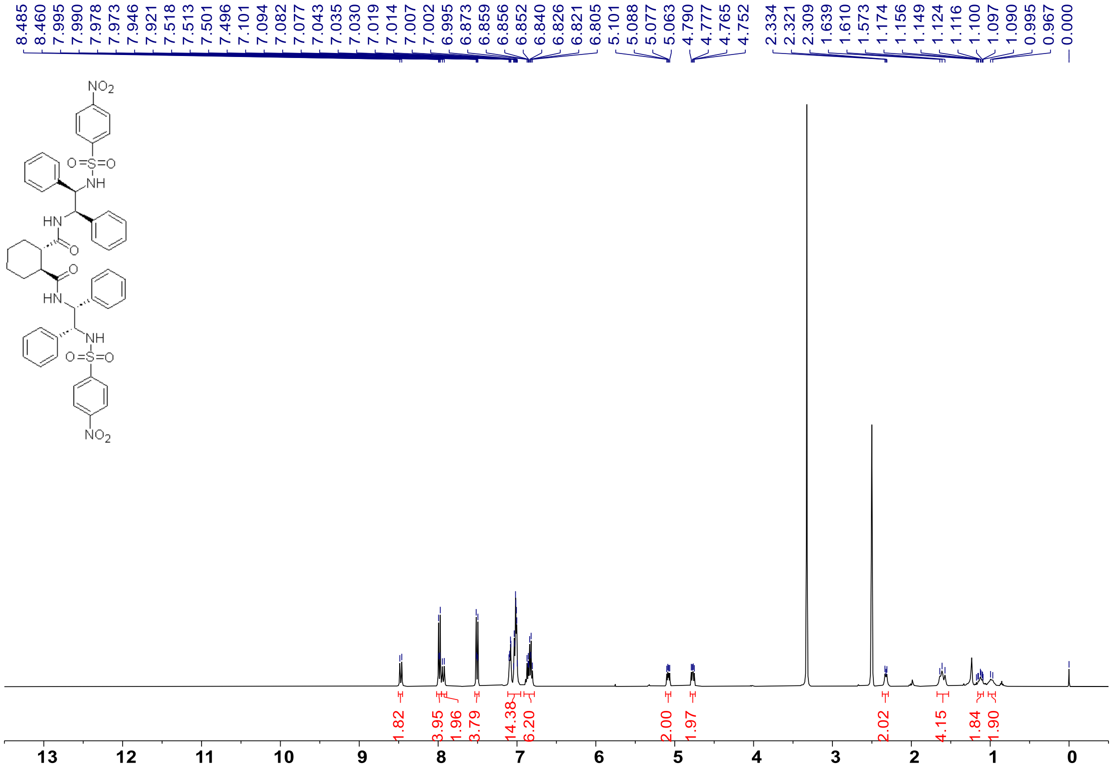


$^{13}$C NMR of (1*S*,2*S*)-*N*[1],*N*[2]-bis((1*R*,2*R*)-2-(4-nitrophenylsulfonamido)-1,2-diphenylethyl)cyclohexane-1,2-dicarboxamide (4e) (101 MHz, DMSO-$d_6$)

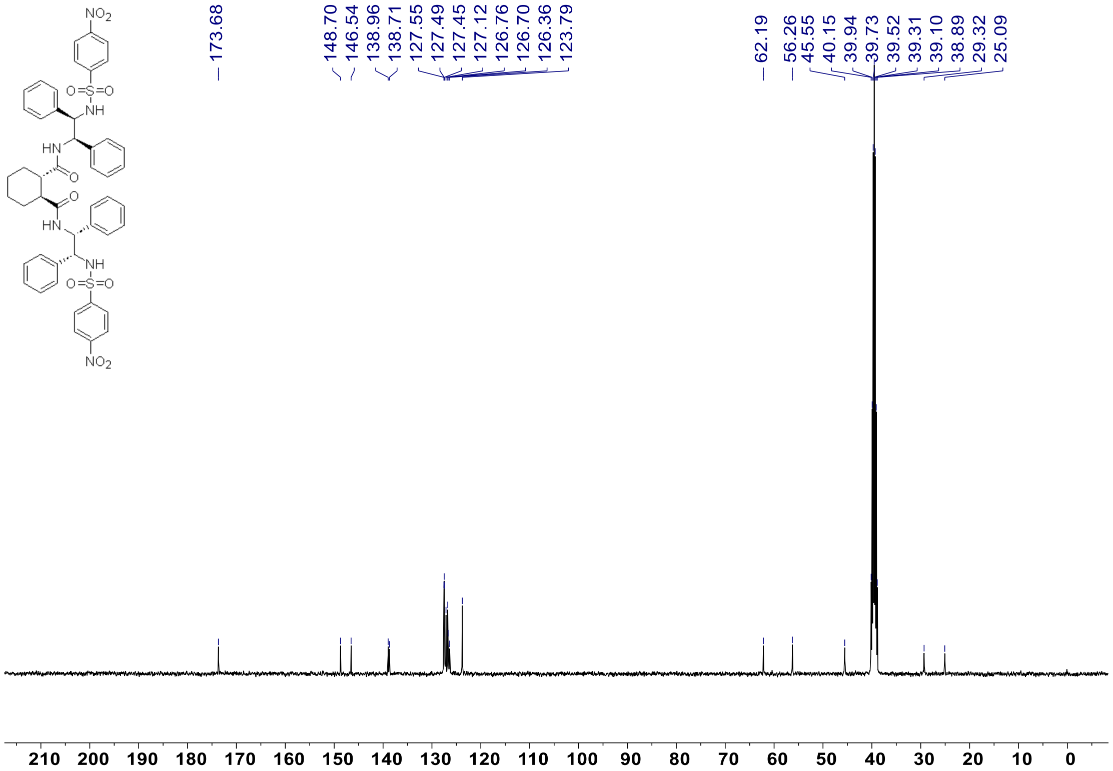

**HRMS (ESI) of (1*S*,2*S*)-*N*[1],*N*[2]-bis((1*R*,2*R*)-2-(4-nitrophenylsulfonamido)-1,2-diphenylethyl)cyclohexane-1,2-dicarboxamide (4e)**

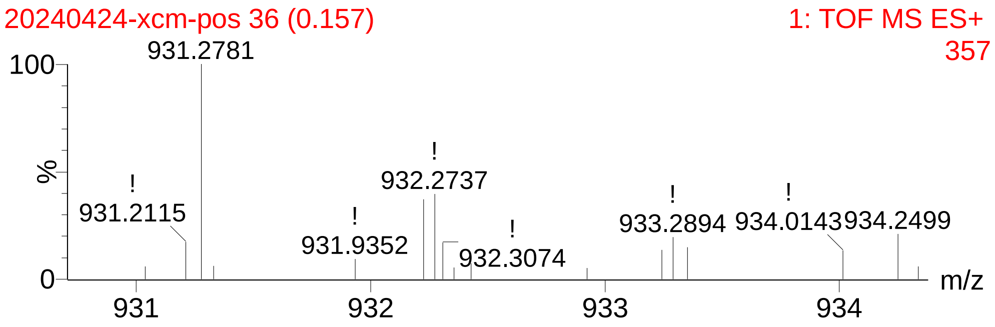

$^{1}$H NMR of (1*S*,2*S*)-*N*$^{1}$,*N*$^{2}$-bis((1*R*,2*R*)-2-(trifluoromethylsulfonamido)-1,2-diphenylethyl)cyclohexane-1,2-dicarboxamide (4f) (400 MHz, DMSO-*d*$_6$)

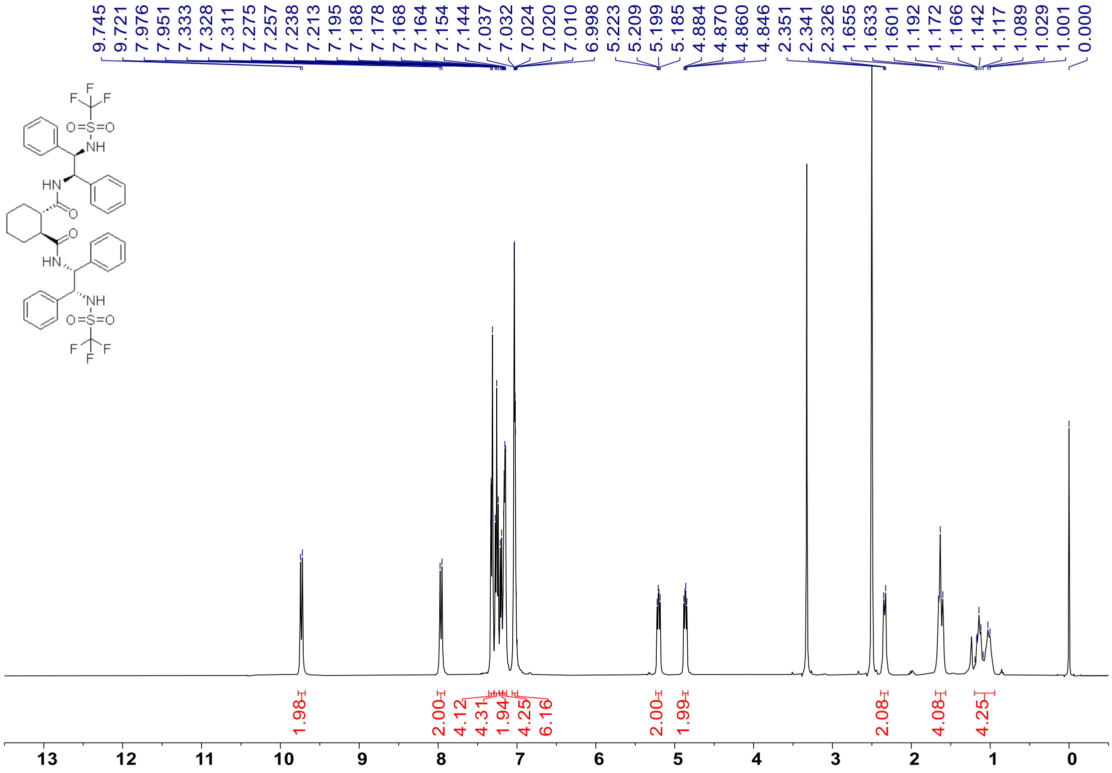

$^{13}$C NMR of (1*S*,2*S*)-*N*$^{1}$,*N*$^{2}$-bis((1*R*,2*R*)-2-(trifluoromethylsulfonamido)-1,2-diphenylethyl)cyclohexane-1,2-dicarboxamide (4f) (101 MHz, DMSO-*d*$_6$)

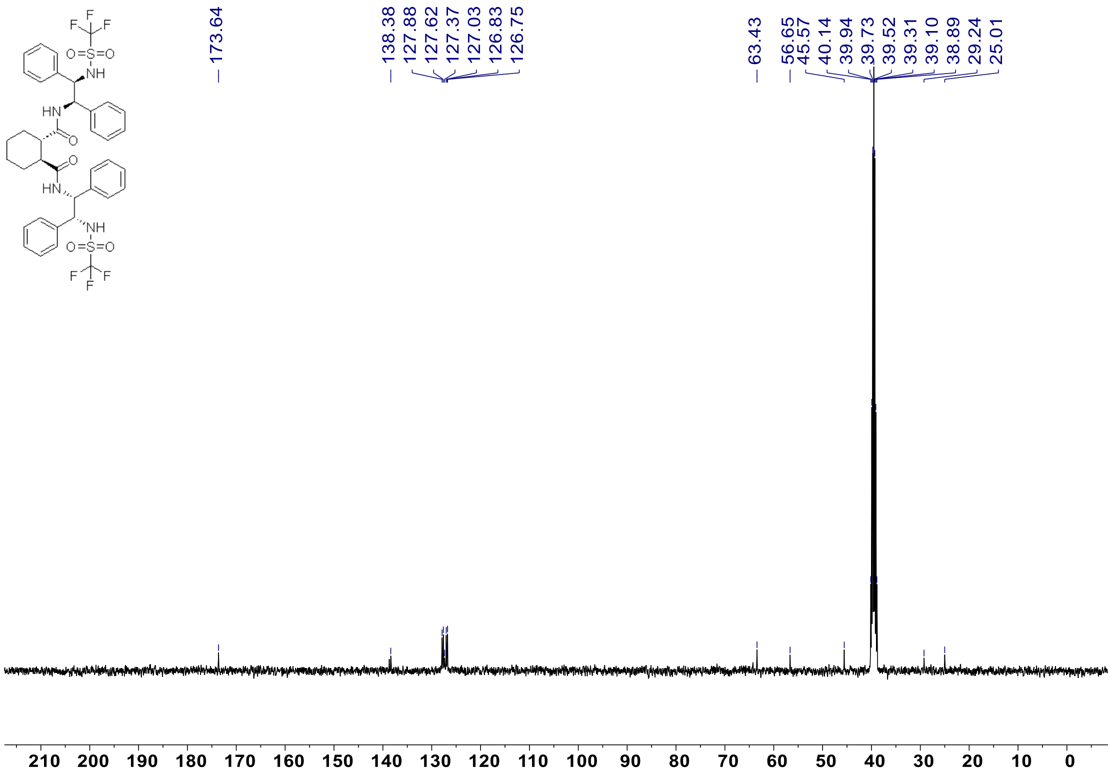

**$^{19}$F NMR of (1*S*,2*S*)-*N*[1],*N*[2]-bis((1*R*,2*R*)-2-(trifluoromethylsulfonamido)-1,2-diphenylethyl)cyclohexane-1,2-dicarboxamide (4f) (376 MHz, DMSO-*d*$_6$)**

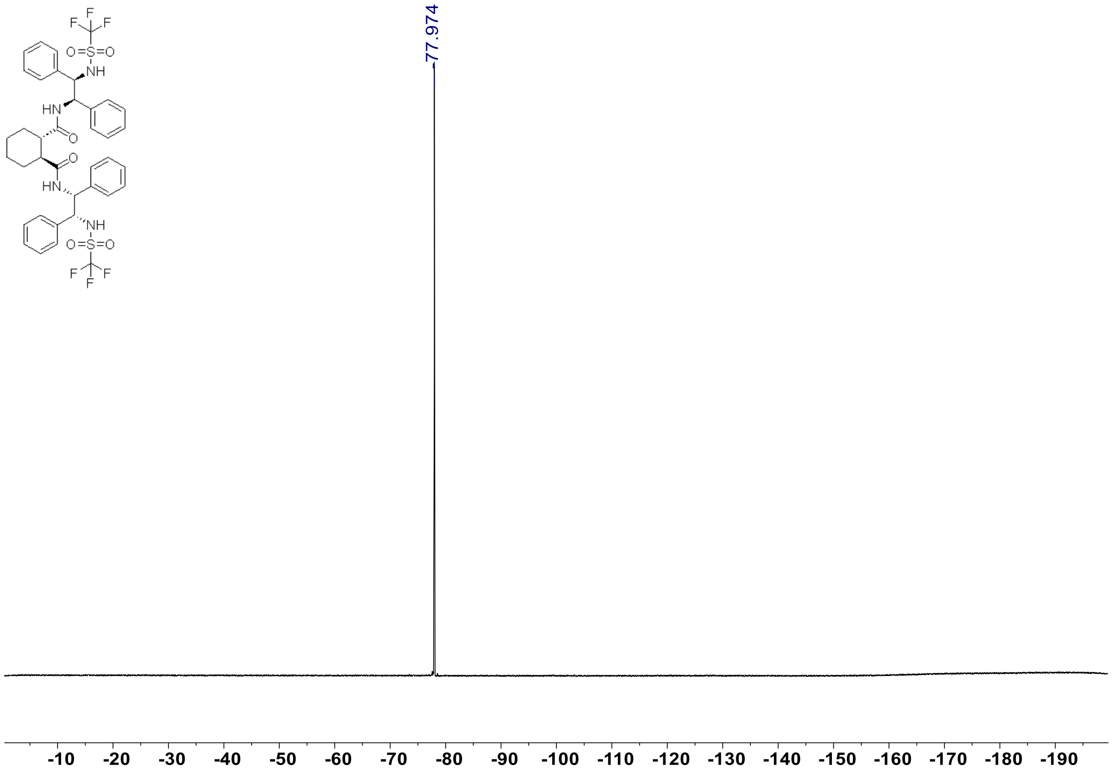


**HRMS (ESI) of (1*S*,2*S*)-*N*[1],*N*[2]-bis((1*R*,2*R*)-2-(trifluoromethylsulfonamido)-1,2-diphenylethyl)cyclohexane-1,2-dicarboxamide (4f)**

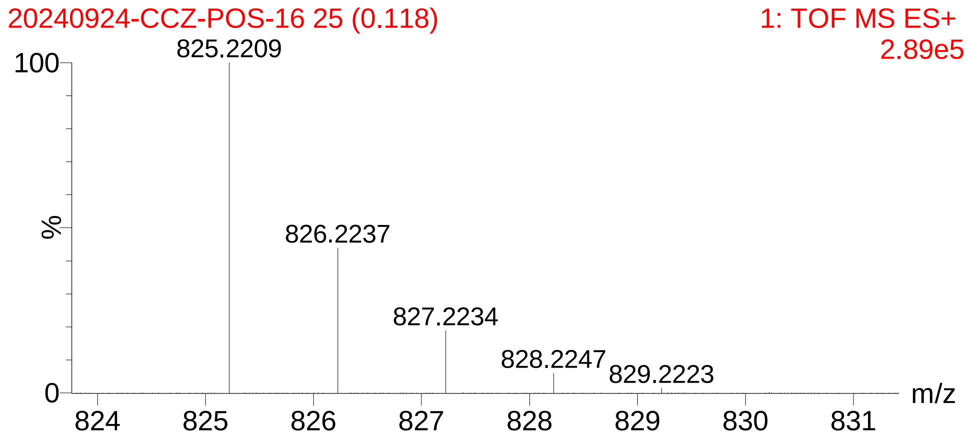

$^{1}$H NMR of (1*S*,2*S*)-*N*[1],*N*[2]-bis((1*S*,2*S*)-2-(4-methylphenylsulfonamido)-1,2-diphenylethyl)cyclohexane-1,2-dicarboxamide (4g) (400 MHz, DMSO-$d_6$)

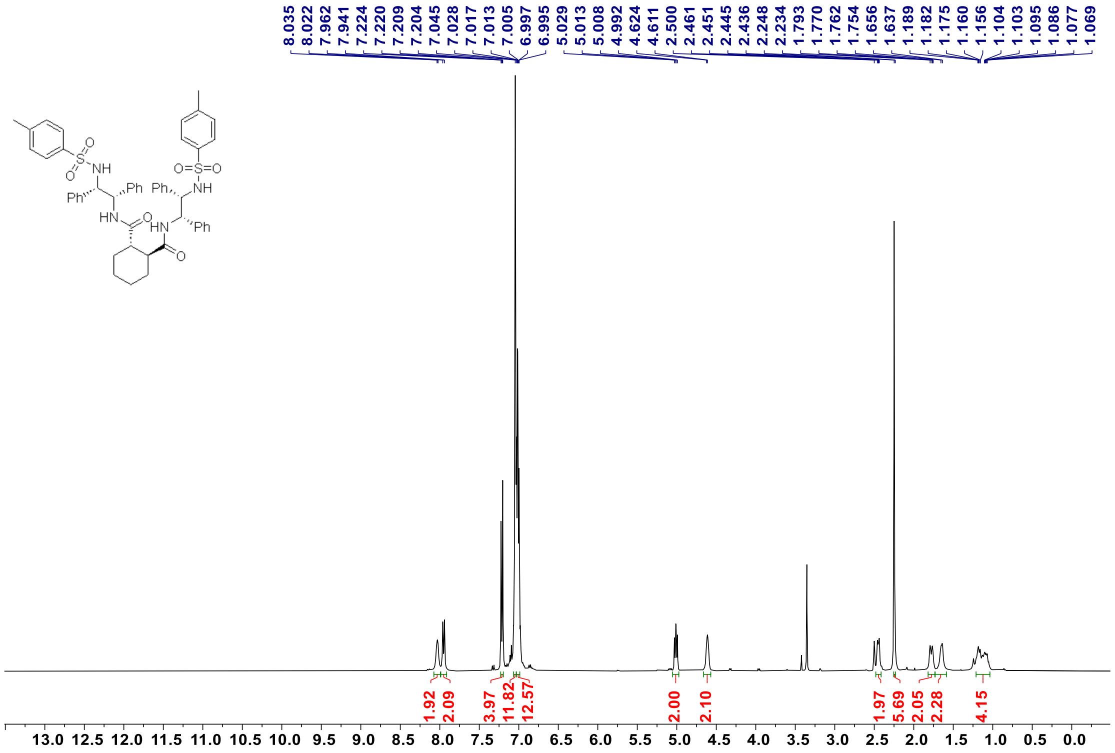


$^{13}$C NMR of (1*S*,2*S*)-*N*[1],*N*[2]-bis((1*S*,2*S*)-2-(4-methylphenylsulfonamido)-1,2-diphenylethyl)cyclohexane-1,2-dicarboxamide (4g) (101 MHz, DMSO-$d_6$)

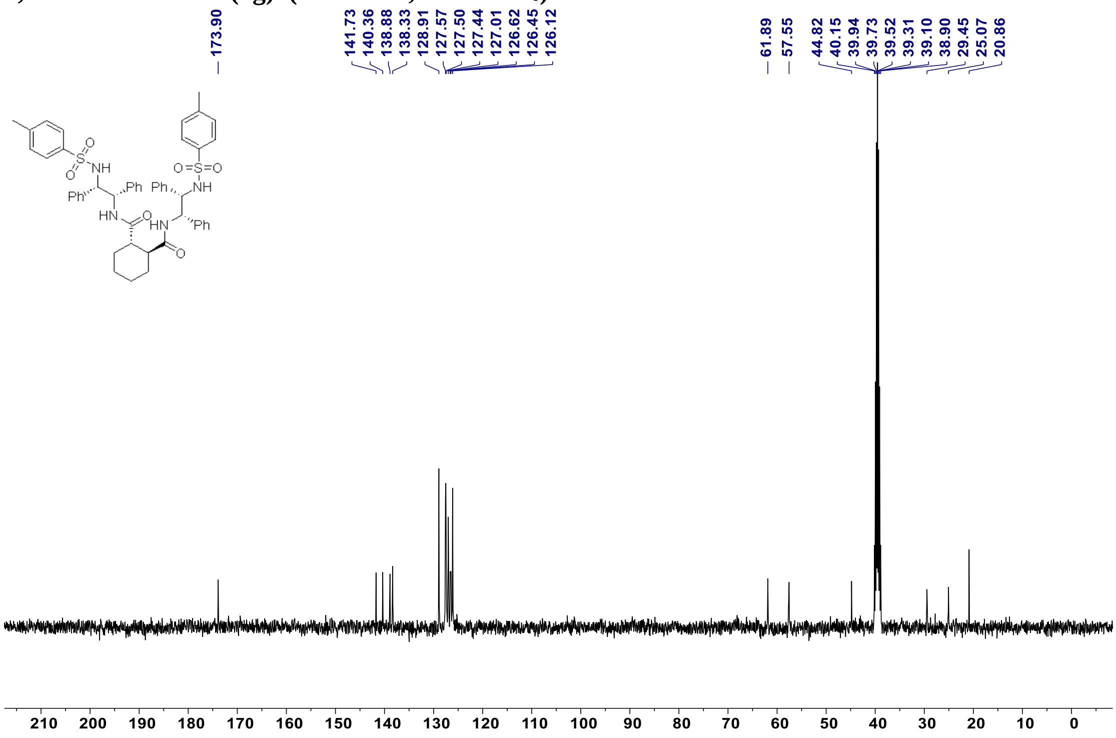

**HRMS (ESI) of (1*S*,2*S*)-*N*$^1$,*N*$^2$-bis((1*S*,2*S*)-2-(4-methylphenylsulfonamido)-1,2-diphenylethyl)cyclohexane-1,2-dicarboxamide (4g)**

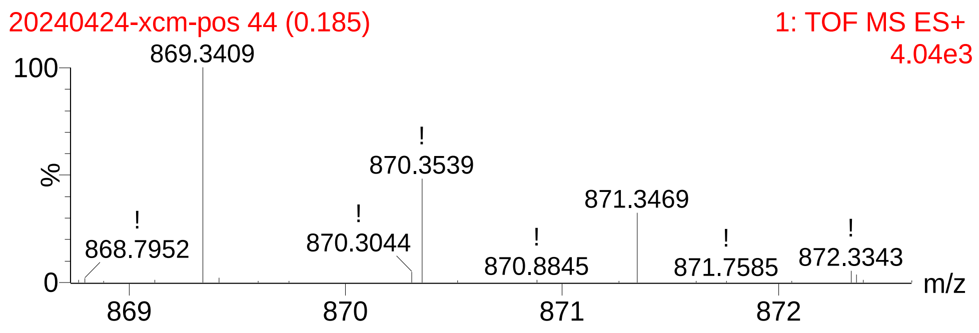

**$^{1}$H NMR of (1*S*,2*S*)-*N*$^{1}$,*N*$^{2}$-bis((1*R*,2*R*)-2-(*tert*-butyloxycarbonyl)-1,2-diphenylethyl)cyclohexane-1,2-dicarboxamide (4h) (400 MHz, DMSO-*d*$_6$)**

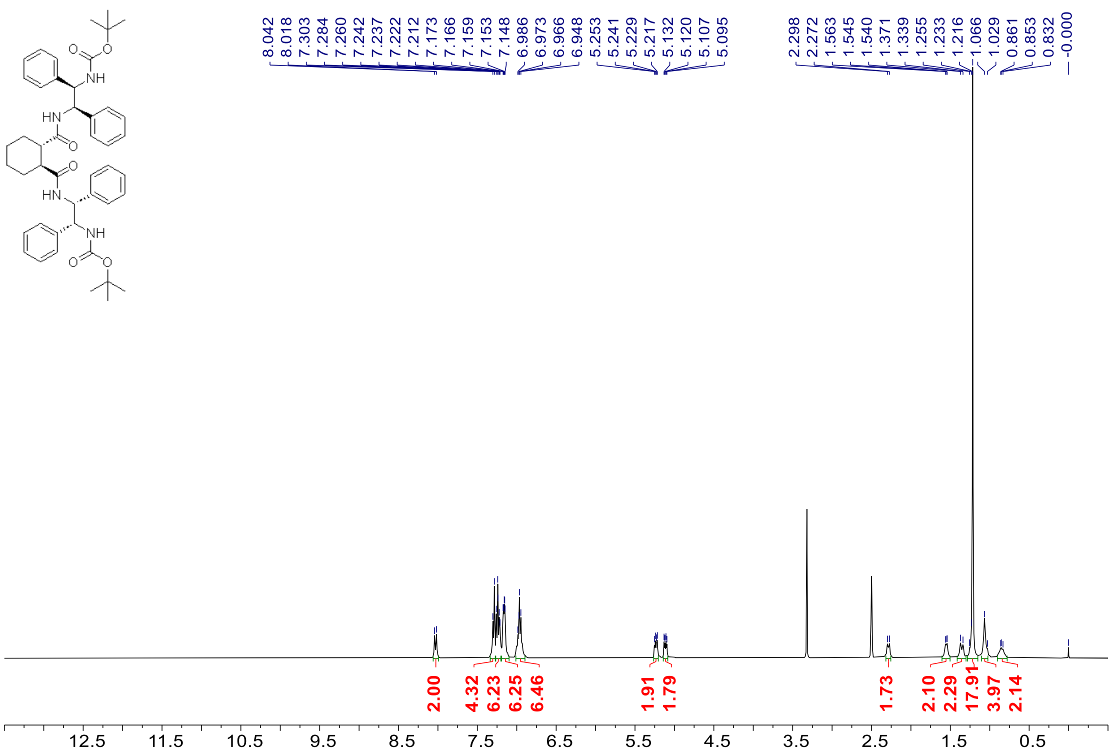


**$^{13}$C NMR of (1*S*,2*S*)-*N*$^{1}$,*N*$^{2}$-bis((1*R*,2*R*)-2-(*tert*-butyloxycarbonyl)-1,2-diphenylethyl)cyclohexane-1,2-dicarboxamide (4h) (101 MHz, DMSO-*d*$_6$)**

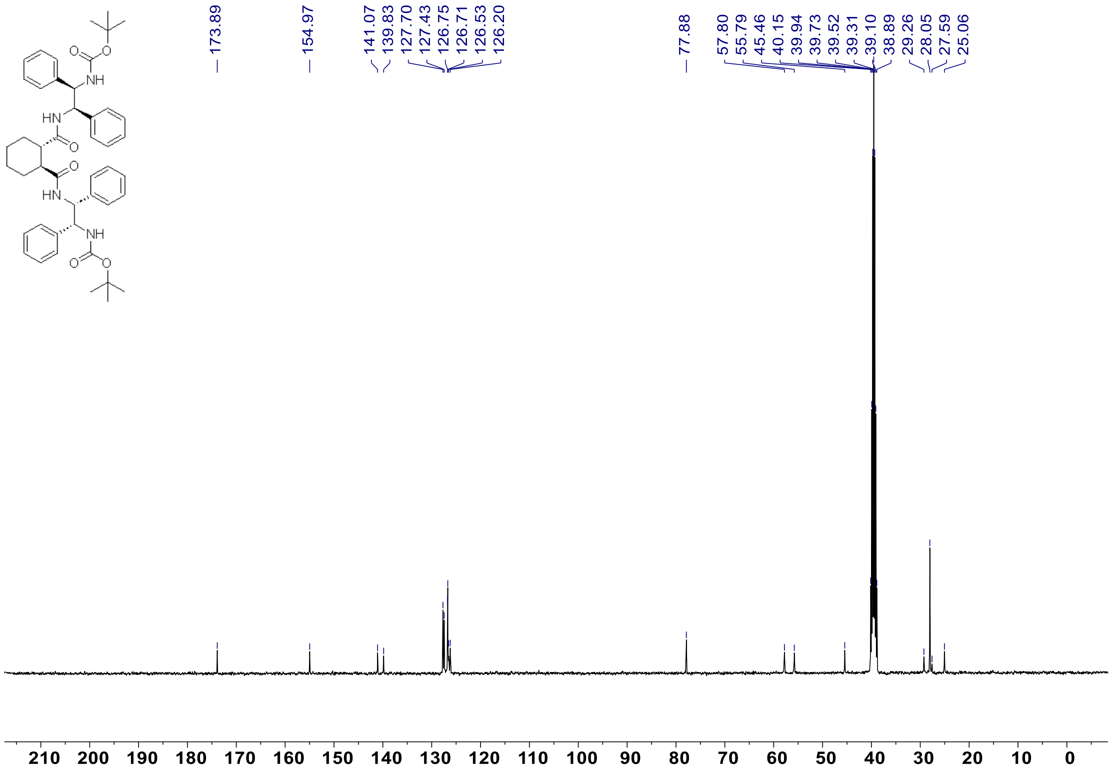

**HRMS (ESI) of (1*S*,2*S*)-*N*[1],*N*[2]-bis((1*R*,2*R*)-2-(*tert*-butyloxycarbonyl)-1,2-diphenylethyl)cyclohexane-1,2-dicarboxamide (4h)**

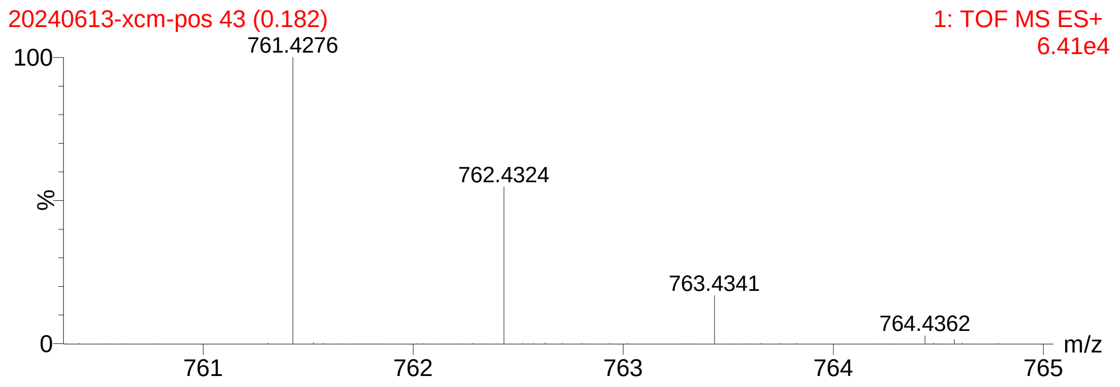

**$^{1}H$ NMR of (1*S*,2*S*)-1,2-bis((4*R*,5*R*)-1-(methylsulfonyl)-4,5-diphenyl-4,5-dihydro-1*H*-imidazol-2-yl)cyclohexane (5a) (400 MHz, $CDCl_3$)**

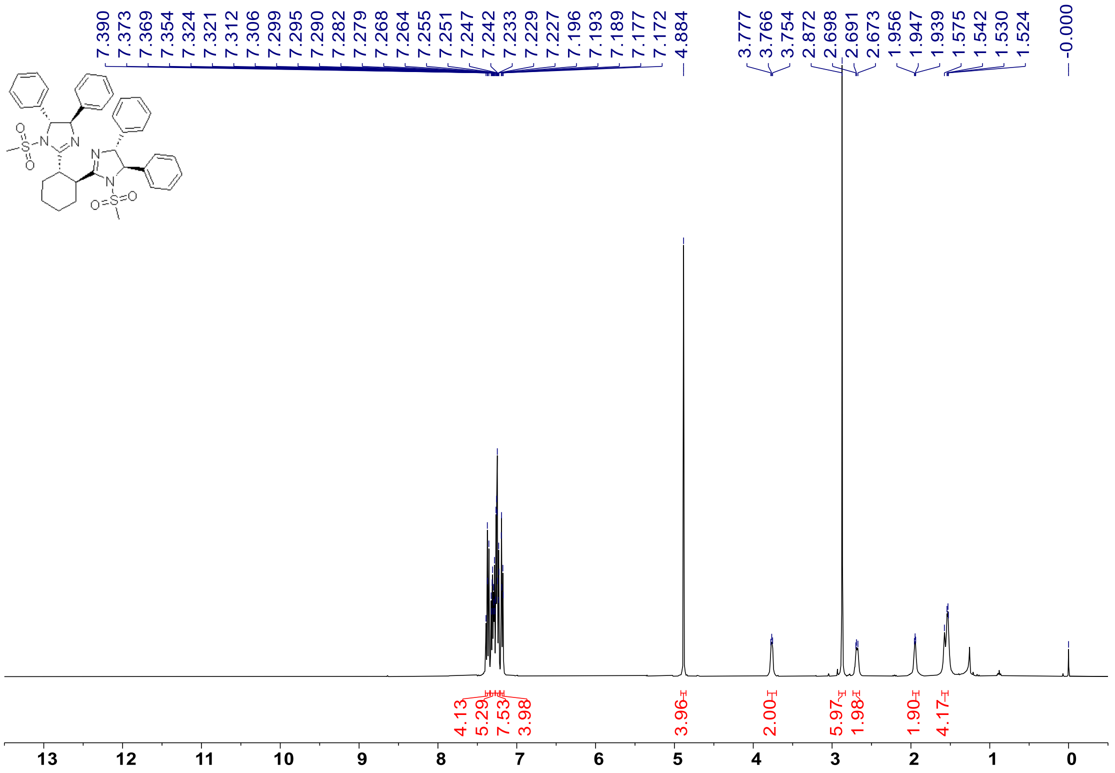


**$^{13}C$ NMR of (1*S*,2*S*)-1,2-bis((4*R*,5*R*)-1-(methylsulfonyl)-4,5-diphenyl-4,5-dihydro-1*H*-imidazol-2-yl)cyclohexane (5a) (101 MHz, $CDCl_3$)**

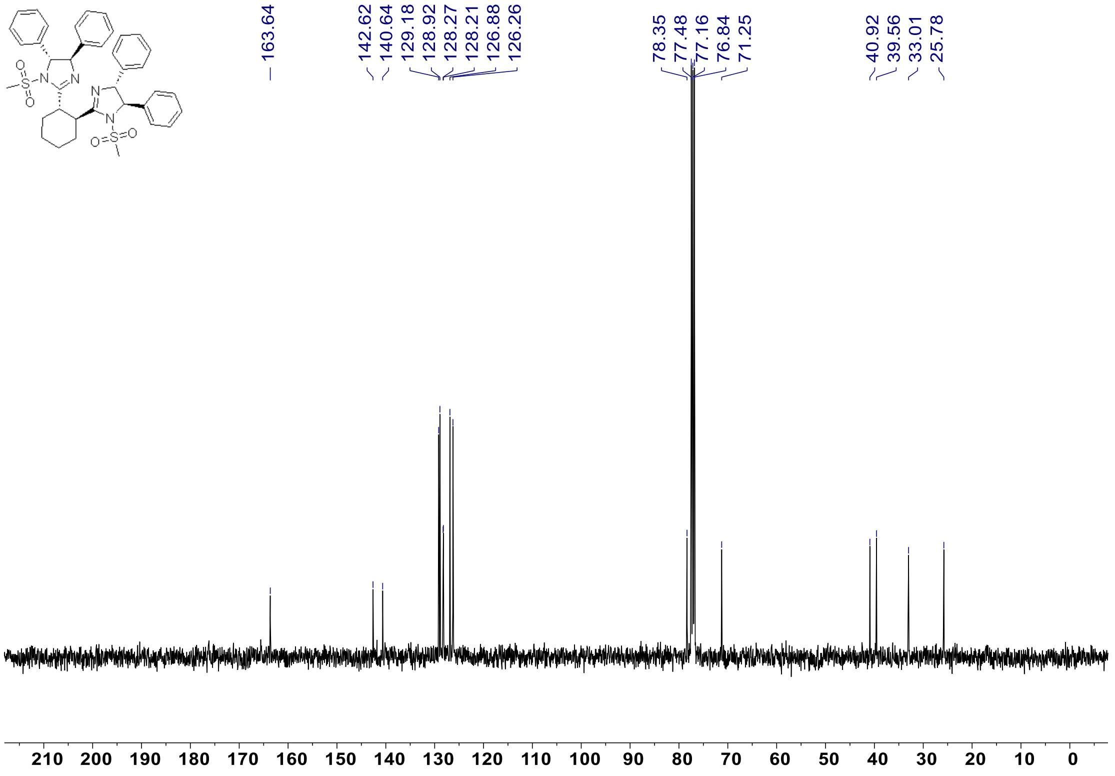

**HRMS (ESI) of (1*S*,2*S*)-1,2-bis((4*R*,5*R*)-1-(methylsulfonyl)-4,5-diphenyl-4,5-dihydro-1*H*-imidazol-2-yl)cyclohexane (5a)**

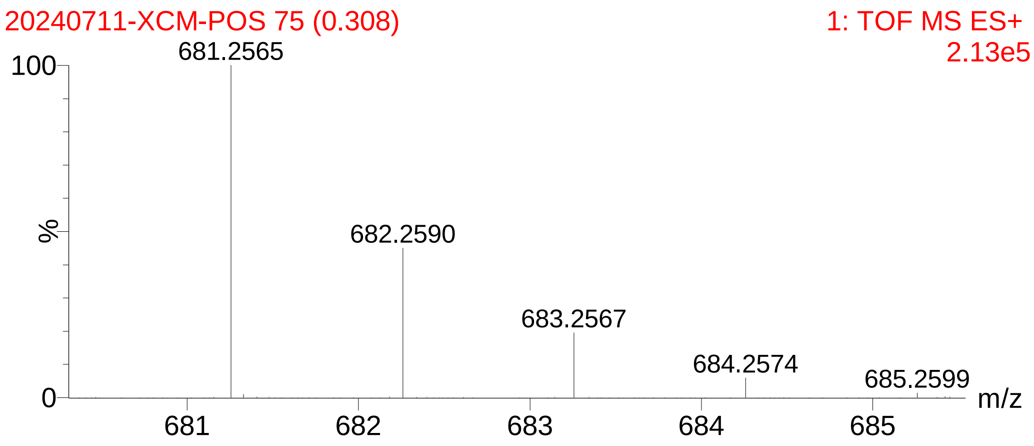

$^{1}$H NMR of (1*S*,2*S*)-1,2-bis((4*R*,5*R*)-4,5-diphenyl-1-(phenylsulfonyl)-4,5-dihydro-1*H*-imidazol-2-yl)cyclohexane (5b) (400 MHz, $CDCl_3$)

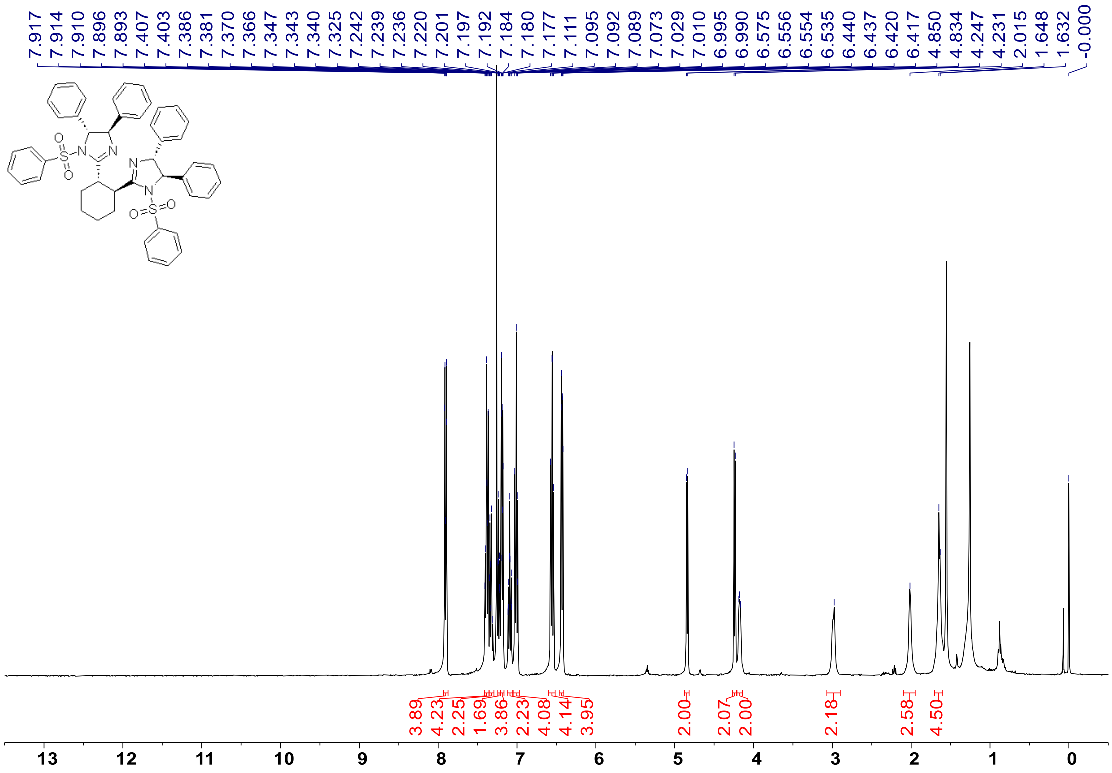


$^{13}$C NMR of (1*S*,2*S*)-1,2-bis((4*R*,5*R*)-4,5-diphenyl-1-(phenylsulfonyl)-4,5-dihydro-1*H*-imidazol-2-yl)cyclohexane (5b) (101 MHz, $CDCl_3$)

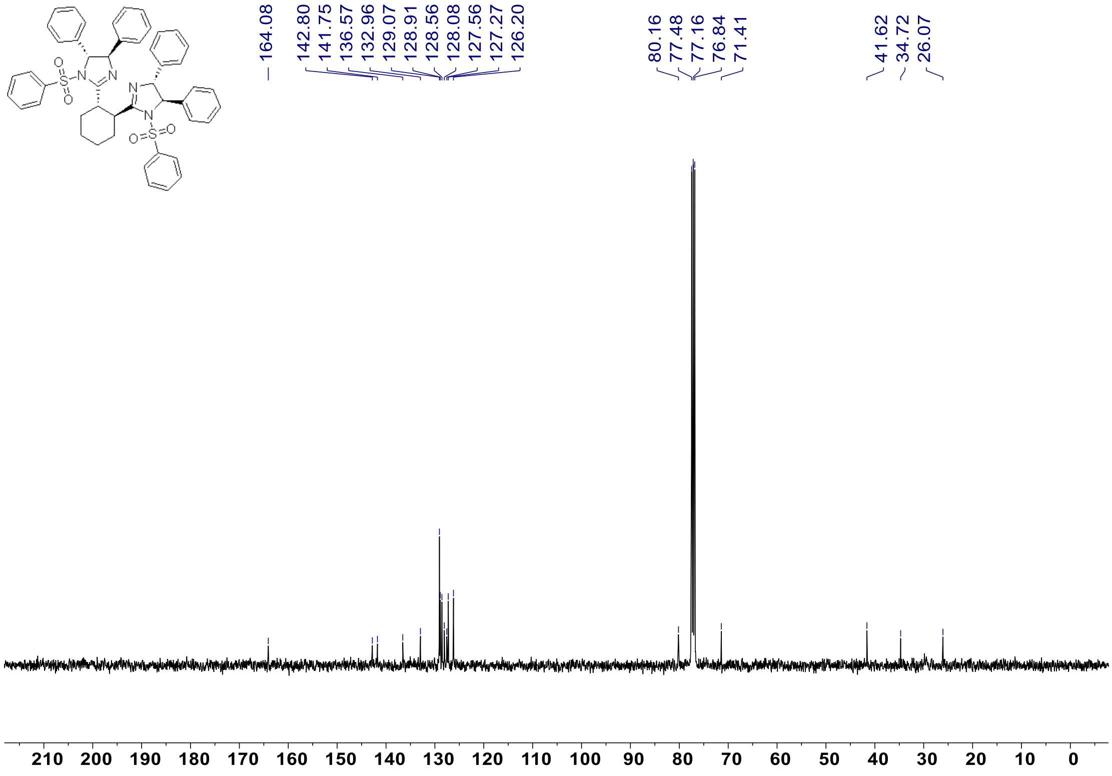

**HRMS (ESI) of (1*S*,2*S*)-1,2-bis((4*R*,5*R*)-4,5-diphenyl-1-(phenylsulfonyl)-4,5-dihydro-1*H*-imidazol-2-yl)cyclohexane (5b)**

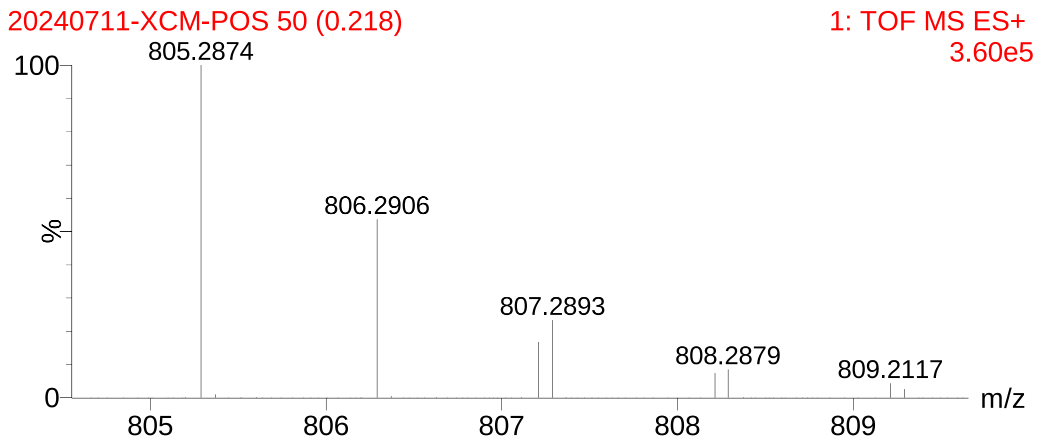

**$^{1}$H NMR of (1*S*,2*S*)-1,2-bis((4*R*,5*R*)-4,5-diphenyl-1-tosyl-4,5-dihydro-1*H*-imidazol-2-yl)cyclohexane (5c) (400 MHz, $CDCl_3$)**

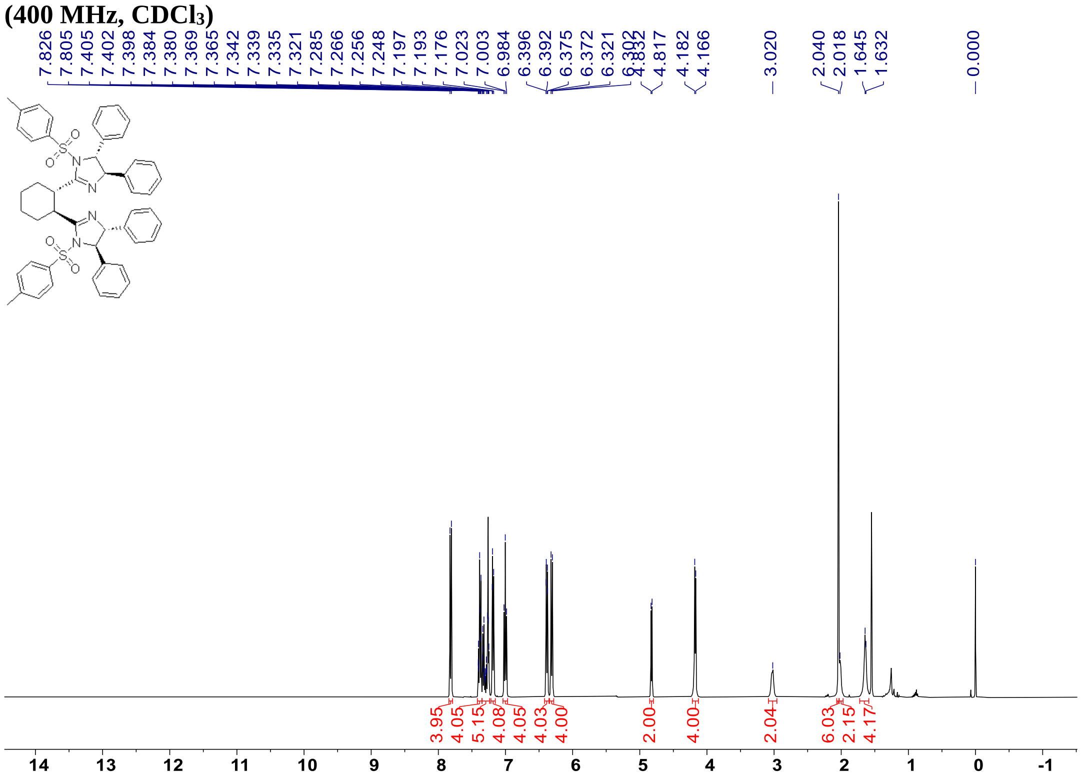


**$^{13}$C NMR of (1*S*,2*S*)-1,2-bis((4*R*,5*R*)-4,5-diphenyl-1-tosyl-4,5-dihydro-1*H*-imidazol-2-yl)cyclohexane (5c) (101 MHz, $CDCl_3$)**

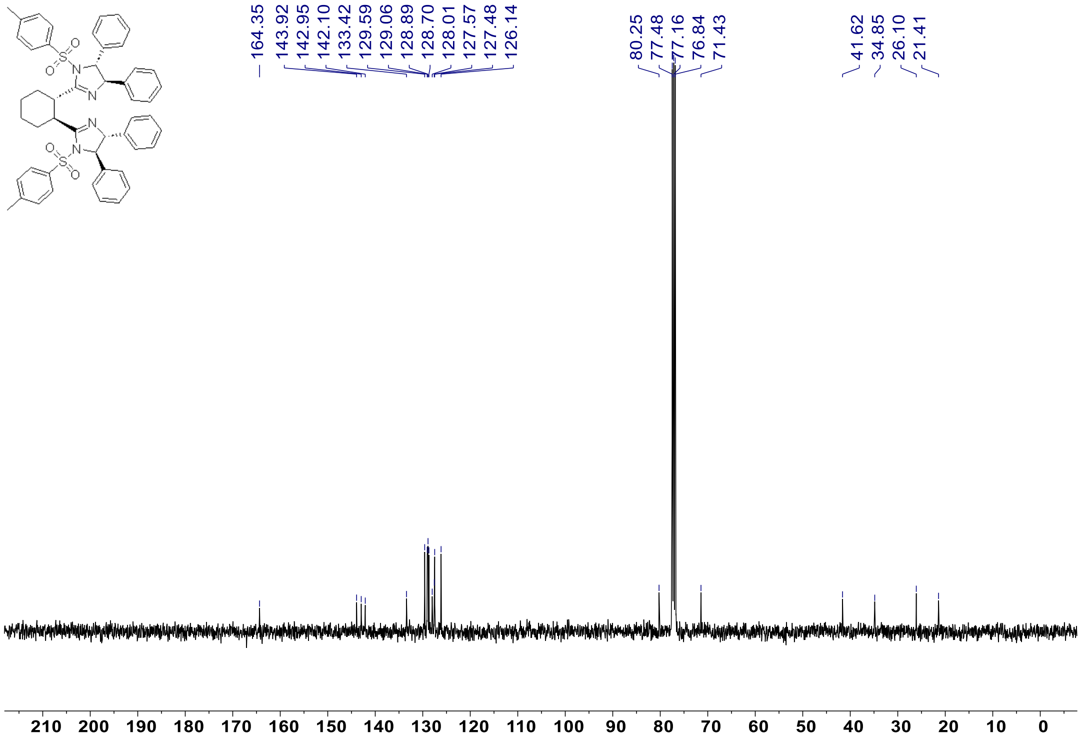

**HRMS (ESI) of (1*S*,2*S*)-1,2-bis((4*R*,5*R*)-4,5-diphenyl-1-tosyl-4,5-dihydro-1*H*-imidazol-2-yl)cyclohexane (5c)**

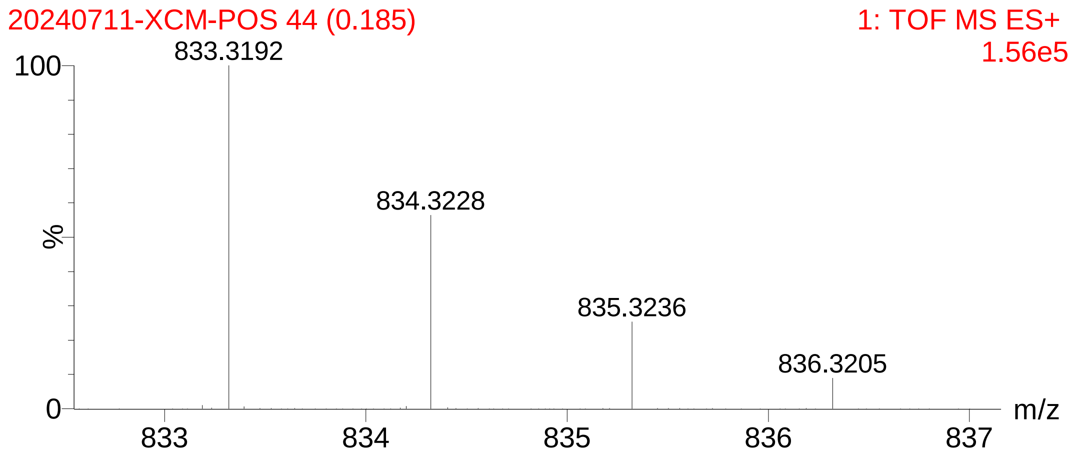

**$^{1}$H NMR of (1*S*,2*S*)-1,2-bis((4*R*,5*R*)-1-((4-methoxyphenyl)sulfonyl)-4,5-diphenyl-4,5-dihydro-1*H*-imidazol-2-yl)cyclohexane (5d) (400 MHz, CDCl$_3$)**

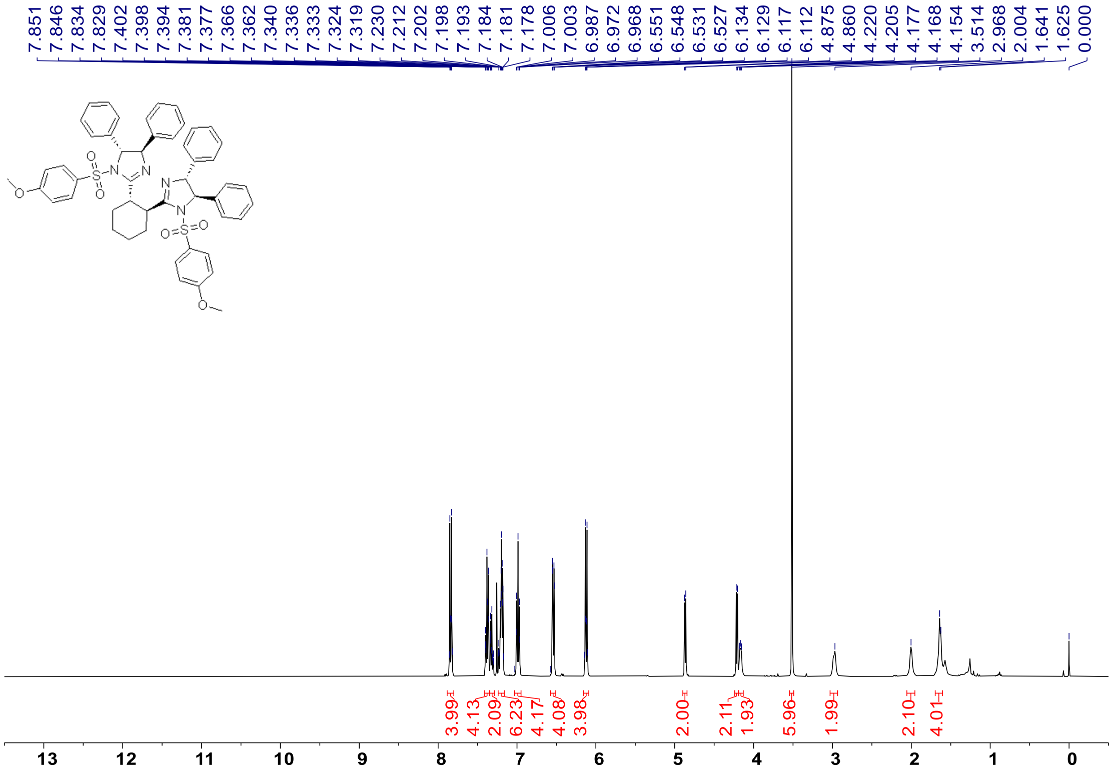


**$^{13}$C NMR of (1*S*,2*S*)-1,2-bis((4*R*,5*R*)-1-((4-methoxyphenyl)sulfonyl)-4,5-diphenyl-4,5-dihydro-1*H*-imidazol-2-yl)cyclohexane (5d) (101 MHz, CDCl$_3$)**

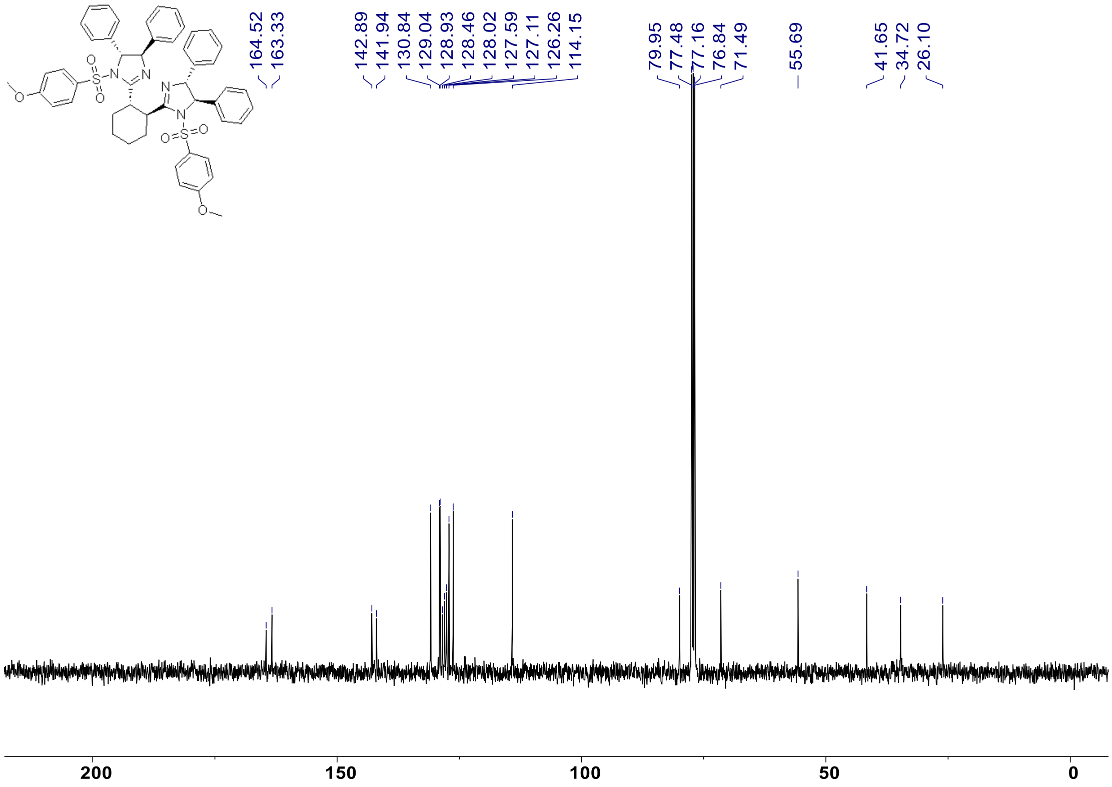

**HRMS (ESI) of (1*S*,2*S*)-1,2-bis((4*R*,5*R*)-1-((4-methoxyphenyl)sulfonyl)-4,5-diphenyl-4,5-dihydro-1*H*-imidazol-2-yl)cyclohexane (5d)**

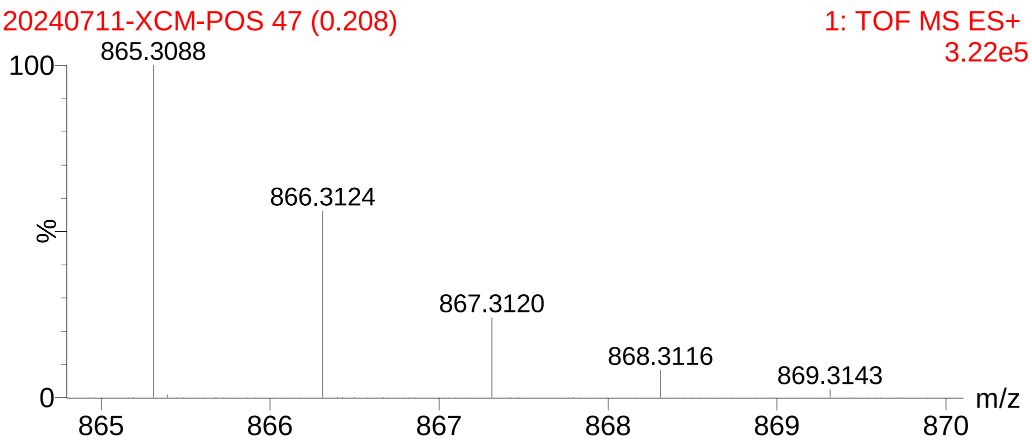

$^{1}$H NMR of (1*S*,2*S*)-1,2-bis((4*R*,5*R*)-1-((4-nitrophenyl)sulfonyl)-4,5-diphenyl-4,5-dihydro-1*H*-imidazol-2-yl)cyclohexane (5e) (400 MHz, $CDCl_3$)

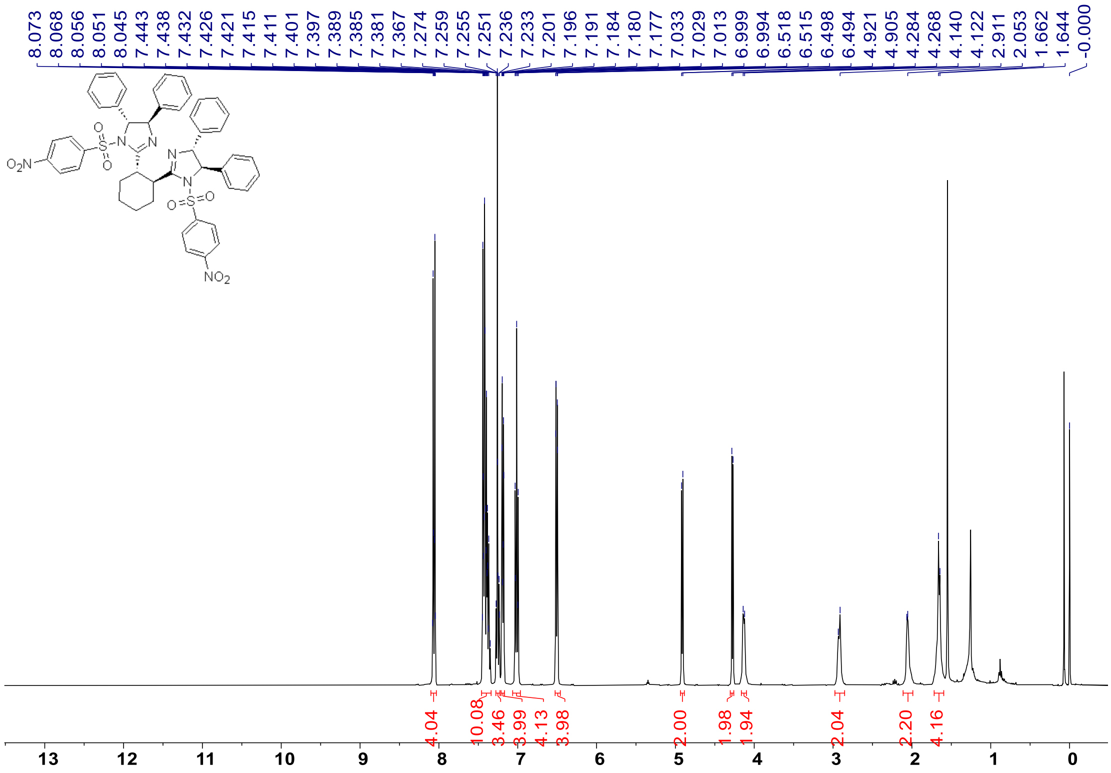

$^{13}$C NMR of (1*S*,2*S*)-1,2-bis((4*R*,5*R*)-1-((4-nitrophenyl)sulfonyl)-4,5-diphenyl-4,5-dihydro-1*H*-imidazol-2-yl)cyclohexane (5e) (101 MHz, $CDCl_3$)

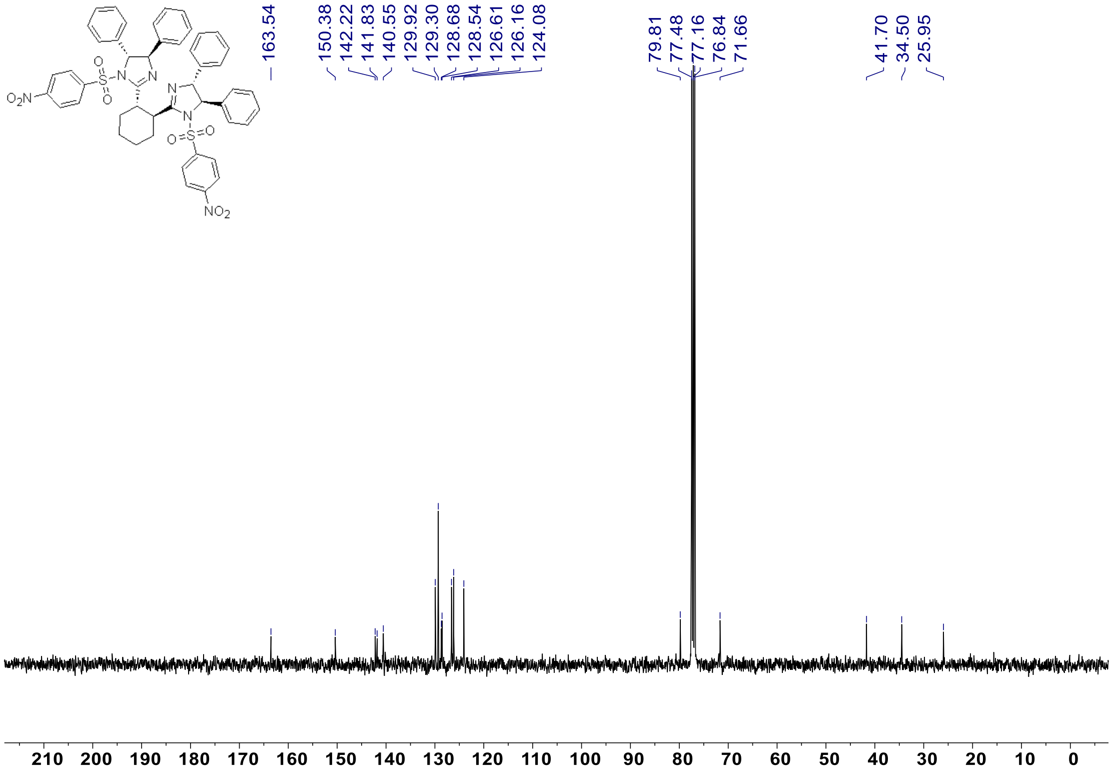

**HRMS (ESI) of (1*S*,2*S*)-1,2-bis((4*R*,5*R*)-1-((4-nitrophenyl)sulfonyl)-4,5-diphenyl-4,5-dihydro-1*H*-imidazol-2-yl)cyclohexane (5e)**

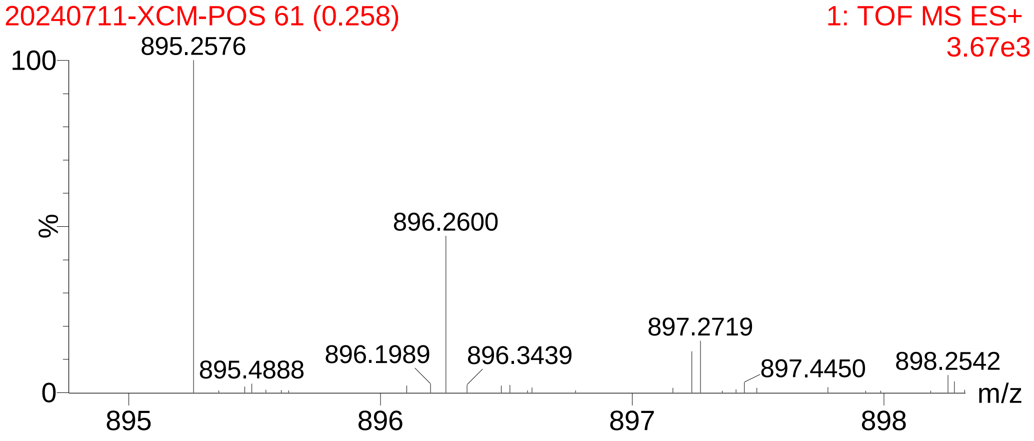

**$^{1}$H NMR of (1*S*,2*S*)-1,2-bis((4*S*,5*S*)-4,5-diphenyl-1-tosyl-4,5-dihydro-1*H*-imidazol-2-yl)cyclohexane (5g) (400 MHz, $CDCl_3$)**

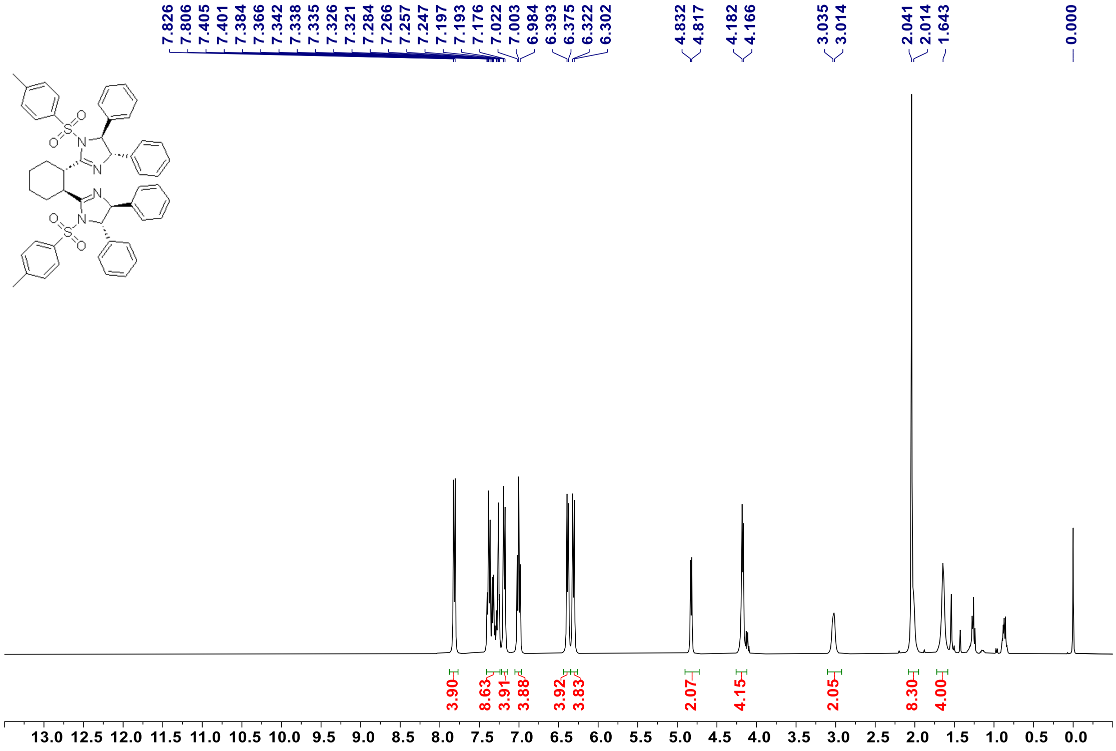


**$^{13}$C NMR of (1*S*,2*S*)-1,2-bis((4*S*,5*S*)-4,5-diphenyl-1-tosyl-4,5-dihydro-1*H*-imidazol-2-yl)cyclohexane (5g) (101 MHz, $CDCl_3$)**

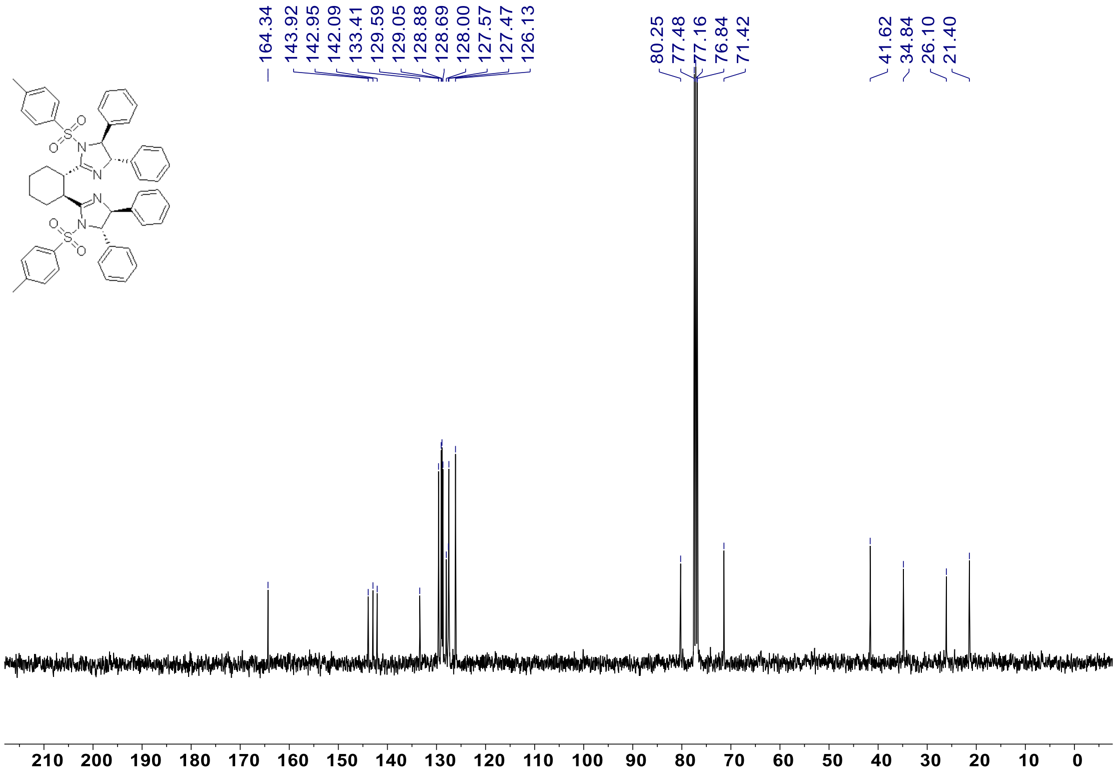

**HRMS (ESI) of (1*S*,2*S*)-1,2-bis((4*S*,5*S*)-4,5-diphenyl-1-tosyl-4,5-dihydro-1*H*-imidazol-2-yl)cyclohexane (5g)**

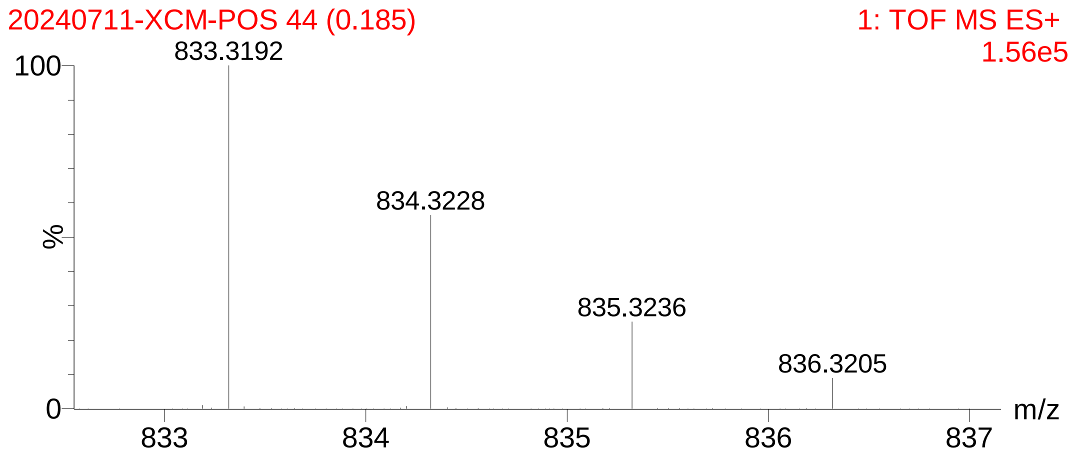

Supplement: File 1 — Analytical data and copies of 1H and 13C NMR spectra of compounds 2 and 4, copies of HRMS spectra of unknown compounds 4 and 5. [file Beilstein_J_Org_Chem-21-1786-s001.pdf]
